# Untargeted Metabolomics Reveals Major Differences in the Plasma Metabolome between Colorectal Cancer and Colorectal Adenomas

**DOI:** 10.3390/metabo11020119

**Published:** 2021-02-19

**Authors:** Tanja Gumpenberger, Stefanie Brezina, Pekka Keski-Rahkonen, Andreas Baierl, Nivonirina Robinot, Gernot Leeb, Nina Habermann, Dieuwertje E G Kok, Augustin Scalbert, Per-Magne Ueland, Cornelia M Ulrich, Andrea Gsur

**Affiliations:** 1 Institute of Cancer Research, Department of Medicine I, Medical University of Vienna, 1090 Vienna, Austria; tanja.gumpenberger@meduniwien.ac.at (T.G.); stefanie.brezina@meduniwien.ac.at (S.B.); 2 International Agency for Research on Cancer, 69372 Lyon, France; keskip@iarc.fr (P.K.-R.); robinot@iarc.fr (N.R.); ScalbertA@iarc.fr (A.S.); 3 Department of Statistics and Operations Research, University of Vienna, 1090 Vienna, Austria; andreas.baierl@univie.ac.at; 4 Department of Internal Medicine, Hospital Oberpullendorf, 7350 Oberpullendorf, Austria; gernot.leeb@krages.at; 5 Division of Preventive Oncology, National Center for Tumor Diseases (NCT) and German Cancer Research Center (DKFZ), 69120 Heidelberg, Germany; nina.habermann@embl.de; 6 Genome Biology, European Molecular Biology Laboratory (EMBL), 69117 Heidelberg, Germany; 7 Division of Human Nutrition and Health, Wageningen University & Research, 6708 Wageningen, The Netherlands; dieuwertje.kok@wur.nl; 8 BEVITAL, A/S, 5021 Bergen, Norway; per.ueland@ikb.uib.no; 9 Population Sciences, Huntsman Cancer Institute, Salt Lake City, UT 84112, USA; Neli.ulrich@hci.utah.edu; 10 Department of Population Health Sciences, University of Utah, Salt Lake City, UT 84108, USA

**Keywords:** colorectal cancer, adenoma, untargeted metabolomics, metabolite profiling

## Abstract

Sporadic colorectal cancer is characterized by a multistep progression from normal epithelium to precancerous low-risk and high-risk adenomas to invasive cancer. Yet, the underlying molecular mechanisms of colorectal carcinogenesis are not completely understood. Within the “Metabolomic profiles throughout the continuum of colorectal cancer” (MetaboCCC) consortium we analyzed data generated by untargeted, mass spectrometry-based metabolomics using plasma from 88 colorectal cancer patients, 200 patients with high-risk adenomas and 200 patients with low-risk adenomas recruited within the “Colorectal Cancer Study of Austria” (CORSA). Univariate logistic regression models comparing colorectal cancer to adenomas resulted in 442 statistically significant molecular features. Metabolites discriminating colorectal cancer patients from those with adenomas in our dataset included acylcarnitines, caffeine, amino acids, glycerophospholipids, fatty acids, bilirubin, bile acids and bacterial metabolites of tryptophan. The data obtained discovers metabolite profiles reflecting metabolic differences between colorectal cancer and colorectal adenomas and delineates a potentially underlying biological interpretation.

## 1. Introduction

Colorectal cancer (CRC) is an acknowledged public health problem representing the third most common cancer-related cause of death and the fourth most commonly diagnosed cancer in the world. In 2018, more than 1.8 million new CRC cases and over 800,000 deaths were reported worldwide [1]. The majority of CRCs are sporadic and usually develop in a slow progression from normal epithelium to precancerous low-risk (LR) and high-risk (HR) adenomas to invasive cancer. This offers significant opportunities for preventive screening and early intervention by effective removal of precancerous adenomas to reduce mortality [2,3].

It is widely accepted that lifestyle and environmental factors like obesity, alcohol consumption, diet and smoking contribute to colorectal carcinogenesis. CRC incidence is further associated with advancing age and gender, underscored by higher age-adjusted rates in males [4]. The most significant protective factors for CRC include plant-based diets, fiber intake and physical activity [5]. Coffee intake, which is metabolically reflected by caffeine, theophylline, or paraxanthine, represents a widely proposed protective factor for CRC, but current evidence remains inconclusive [6,7]. Increasing evidence attributes CRC development to alterations in the intestinal microbiota. Microbial metabolites such as secondary bile acids have been shown to promote carcinogenesis [8]. Cancer-specific scavenging pathways driving unremitting cell proliferation involve nucleic acid, fatty acid and amino acid metabolism [9].

Metabolomics, the study of changes of the metabolome composition upon a particular stimulus or condition, is used to identify metabolites that reflect the influences from environmental, lifestyle, and endogenous factors [8,10,11]. The metabolome, the sum of all metabolites in a particular biological sample, is a close molecular representation of the phenotype reflecting physiological or pathological state [12]. Untargeted metabolomics is suitable to detect hundreds of metabolites in biological samples and to provide insights into metabolic changes [13,14]. In contrast to targeted metabolomics, untargeted metabolomics analyses follow a hypothesis-free approach to discover metabolites that can be mapped to networks and pathways [13]. Liquid chromatography-mass spectrometry (LC-MS) has become invaluable for analyzing polar and nonpolar metabolites. Increasing both selectivity and data content, LC-MS has emerged as a leading technology for complex metabolomics samples such as human blood [13]. Differences in metabolite profiles along colorectal carcinogenesis have been reported using serum, plasma, tissue, or fecal samples [15,16,17,18]. These previous studies often yield inconsistent findings or small sample sizes, indicating the necessity to extend or confirm current hypotheses.

The overall aim of this study was to perform metabolite profiling applying an untargeted metabolomics approach using plasma samples from 88 patients with incident CRC, 200 patients with HR adenomas and 200 patients with LR adenomas selected from the “Colorectal Cancer Study of Austria” (CORSA) biobank.

## 2. Results

### 2.1. Study Population

Demographics and clinical characteristics of the study population are shown in Table 1. The study cohort consisted of 68.2% men in the CRC group and 66.0% men in the HR as well as the LR adenoma groups. CRC participants were on average slightly older (70.0 years) compared to HR (65.4 years) or LR adenoma patients (66.0 years). The LR adenoma group had the highest proportion of never smokers. In general, the distribution of covariates was balanced between the three participant groups.

### 2.2. Metabolic Features Derived from Untargeted Metabolomics Analysis

Alignment of two analytical batches yielded 4595 detected features, of which 983 were carried forward after data preprocessing. The complete list of statistically significant features associated with respective case-control status is presented in Supplementary Table S1. The numbers of features with FDR-corrected, statistically significant *p*-values (herein referred to as *q*-value) are shown in Table 2 for the three compared groups. We detected in total 409 significant features in the CRC versus (vs.) the HR and LR adenomas group, 367 when comparing CRC vs. HR adenomas, and 384 in the CRC vs. LR adenomas comparison group. A sensitivity analysis excluding data from CRC stage III-IV did not reveal any significant influence by advanced stage CRC into the main analysis (data not shown).

### 2.3. Metabolic Differences in CRC Compared to Colorectal Adenomas

**Figure 1:**
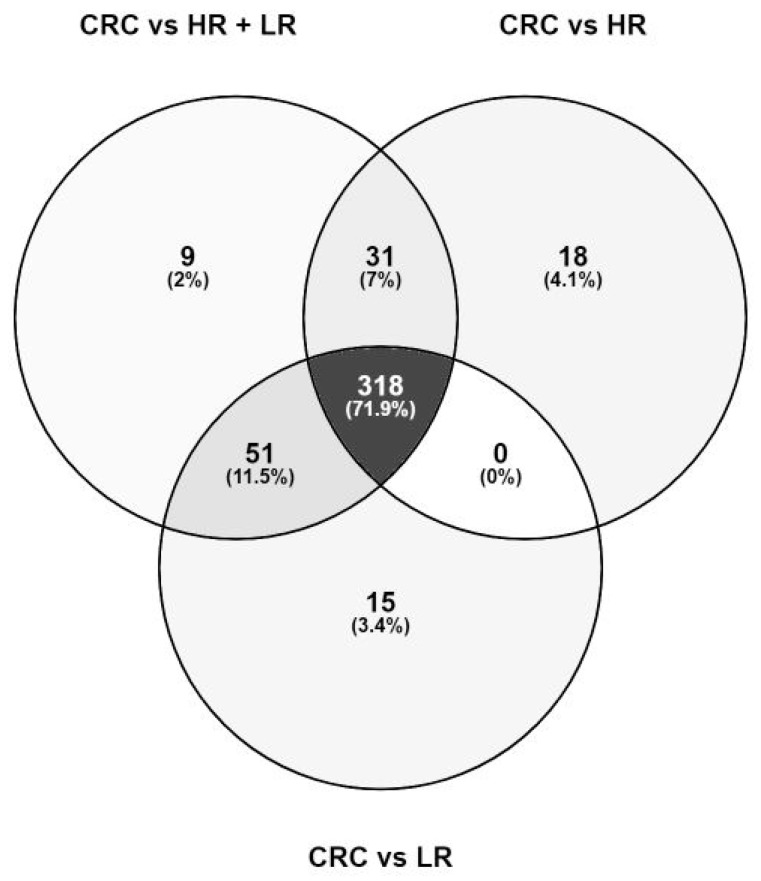
Venn diagram showing the overlap of 442 significant features detected in the three comparison groups. Nearly all metabolites were shared across the three comparison groups. A subset of metabolites was specifically detected within the CRC vs. HR and LR adenoma group (2%), CRC vs. HR adenomas (4.1%), and CRC vs. LR adenomas (3.4%).

In total we detected 442 distinct significant metabolite features, of which 71.9% (318 features) overlap across all three case-control comparison groups (Figure 1). The list of significant metabolic features specific for each comparison group, respectively, is given in Supplementary Table S2. No significant difference between the HR group and LR group could be detected via univariate logistic regression (data not shown). These results prompted us to combine the HR and LR adenomas into one adenoma group for further analyses. Noteworthy, subsequent presented data focuses on results generated solely from the CRC vs. HR and LR comparison.

Out of the statistically significant molecular features we could identify 48 metabolites comparing CRC with HR and LR adenomas. These identified metabolites combined according to metabolic pathways are listed in Table 3. Positive or inverse associations with CRC are reflected by ORs above (red font) or below 1 (green font), respectively. In concordance with the Metabolomics Standards Initiative (MSI), 24 metabolites reached identification levels 1 and 24 resulted in level 2. Retention times and the fragmentation (MS/MS) spectra of the identified metabolites compared to an authentic chemical standard can be taken from Supplementary File S4.

### 2.4. Metabolic Enrichment and Pathway Analysis

When subjecting the 48 identified metabolites comparing CRC with HR and LR adenomas to metabolite sets enrichment and pathway analysis, the major relevant pathways were the caffeine metabolism, glycerophospholipid metabolism, taurine and hypotaurine metabolism, and pathways involving amino acid metabolism. (Supplementary Figure S3).

## 3. Discussion

Untargeted metabolomics data from CORSA comprising 88 participants diagnosed with CRC, 200 patients with HR, and 200 patients with LR adenomas were used to investigate potential metabolic profiles and pathways in relation to colorectal carcinogenesis. We detected in total 442 statistically significant metabolic features in this untargeted metabolomics profiling. The large number of statistically significant features detected during this study suggests major differences in the plasma metabolome between the patients diagnosed with stage I-IV CRC at the time of diagnosis and colorectal adenomas. The majority of statistically significant metabolic features were commonly shared across all three compared groups. Furthermore, we did not reveal any significant difference in the metabolic profiles between HR and LR adenomas. Consequently, we combined HR and LR adenomas into one adenoma group to be compared against CRC. The applied untargeted metabolomics approach allowed a non-hypothesis-driven analysis to identify metabolites and pathways linked to the progression from adenomas to CRC.

Within the MetaboCCC consortium, Geijsen and Brezina et al. previously performed an untargeted metabolomics screening on plasma samples from patients diagnosed with CRC and controls of the CORSA and the ColoCare study [19]. ColoCare is an ongoing multicenter prospective cohort study initiated in Heidelberg in 2010 [20]. In this preceding study, multiple logistic regression models were used to test the association between metabolic features and disease state. In total, 15 metabolites were identified to exhibit significant differences between CRC patients and controls [19].

Geijsen and Brezina et al. reported circulating plasma levels of 1-methylnicotinamide to be notably decreased in CRC patients compared to controls [19]. In the present study, 1-methylnicotinamide was inversely associated with CRC. 1-Methylnicotinamide can be biosynthesized in humans through the catalytic action of the enzyme nicotinamide N-methyltransferase. Nicotinamides serve as precursors for nicotinamide adenine dinucleotide (NAD^+^), a key molecule involved in energy metabolism [21].

Metabolites of the carnitine cycle play a vital role in fatty acid metabolism and mitochondrial fatty acid transport, but can also impact the composition of gut microbiota [22]. We identified many acyl carnitines as associated with CRC, confirming their possible role along colorectal carcinogenesis.

Bilirubin has been reported to possess important antioxidant and anticancer functions and was considered as an efficient prognostic biomarker for overall survival in advanced CRC [23,24]. Lower plasma bilirubin levels were found in CRC compared to healthy controls [19]. Circulating plasma bilirubin levels and CRC risk were reported to differ by sex, reflected by a positive association with CRC risk among men [25]. Our findings on lower levels of plasma bilirubin in CRC compared to adenomas further emphasize the involvement of bilirubin in colorectal carcinogenesis.

Geijsen and Brezina et al. reported higher plasma concentrations of taurine, a member of the bile acid metabolism, in CRC patients compared to controls [19]. Other studies found taurine increased in serum and tissue of CRC patients [18,26]. Taurine has further been shown to possess apoptotic effects in human CRC cells [27]. There is evidence linking colonic microbiota composition and dietary taurine intake with an elevated CRC risk [28]. In our study, CRC patients were mostly overweight. However, detailed dietary information is not available within CORSA. Taurine was detected with higher levels in CRC patients compared to adenomas and the taurine and hypotaurine metabolism was one of the most relevant pathways in our enrichment and pathway analyses.

Coffee ranges among the most consumed beverages worldwide and its consumption has been associated with a lower risk of CRC, which might be explained by the many phytochemicals contained in coffee [29,30]. Caffeine and several compounds of the caffeine metabolism have been found to be significantly altered in controls compared to CRC and colorectal adenomas [31]. Previous studies have shown the inhibitory effect of caffeine on colon cancer cell proliferation in vitro [32]. Contrary, coffee intake was not associated with colon cancer risk in other studies [33]. We found several metabolites involved in caffeine metabolism (caffeine, theobromine, and theophylline) at higher levels in CRC cases than in adenomas. Further epidemiologic studies are needed to determine the role of caffeine and other coffee phytochemicals on CRC risk.

Hypoxanthine, a naturally occurring purine derivative involved in nucleotide metabolism, has been detected at higher plasma levels in CRC patients and as well as in CRC tumor tissue compared to normal plasma and tissue [19,34]. However, a study published by Long et al. reported lower hypoxanthine serum levels in patients diagnosed with CRC or adenomas compared to healthy controls [31]. In our study, hypoxanthine was elevated in CRC patients compared to adenomas, suggesting a possible role in CRC development.

Interestingly, we observed higher concentrations of several metabolites from the tryptophan pathway in CRC cases when compared with adenomas. Those include several bacterial metabolites of tryptophan such as indole acetic acid, indole propionic acid, and indole lactic acid, which points towards a contribution of the gut microbiota in CRC development [35,36]. Further we also detected isatin, also known as 1H-indole-2,3-dione, an endogenous metabolite of tryptophan belonging to the class of organic compounds known as indolines. Isatin is an oxidation product of indole that originates from tryptophan being associated with the gut microbial metabolism [37,38]. In our study, isatin was significantly higher in the CRC patients than adenomas, which maybe be associated to its cytotoxic effect. To our knowledge there are no previous metabolomics studies that have found isatin associated with colorectal carcinogenesis.

Several lysophosphatidylcholines (LysoPCs) have been shown to be significantly decreased in CRC cases compared to controls [19,39,40]. In our study, several lipids from the LysoPCs, PCs, and diacylglycerol classes were detected at lower levels in plasma from CRC patients compared to adenomas. We identified five LysoPCs to be specific for the CRC against LR adenomas comparison group. Moreover, the linoleic acid and glycerophospholipid metabolism ranged among the major metabolic pathways resulting from our pathway analysis, supporting the hypothesis of a derailed lipid metabolism in cancer [9]. Further, we detected choline at decreased levels in CRC samples, which is in line with previous investigations showing that plasma choline tends to be positively associated with rectal cancer risk [41]. However, it still remains unclear whether dietary intake of choline is associated with CRC risk [42].

Polyunsaturated fatty acids including docosahexaenoic acid (DHA) have been linked with decreased CRC risk before [43]. Evidence is accumulating that polyunsaturated fatty acids may have preventive properties for CRC [44]. Within the presented study we have found decreased levels of DHA in CRC patients compared to LR and HR adenoma patients.

We detected proline at higher levels in plasma from CRC patients compared to adenomas, whereas levels of valine were decreased in CRC samples. A previous study reported higher levels of proline and valine in CRC compared with adenomas [45], and decreased levels were detected in CRC compared to compared to controls [46]. Valine has repeatedly been reported to be decreased in CRC [16,19,46,47]. Amino acids are the essential building blocks required for protein synthesis and have been repeatedly studied and reported to be up- and downregulated in CRC biospecimen. This fact reflects the excessive protein demand for continuous cancer cell growth and proliferation and a derailed protein turnover within the tumor microenvironment [48].

One strength of this study is that the recruitment of CORSA is in close cooperation with the CRC screening program “Burgenland Prevention Trial of Colorectal Cancer Disease with Immunological Testing” (B-PREDICT). In course of this two-stage screening program, all Fecal Immunochemical Test (FIT)-positive participants receive a colonoscopy. Within B-PREDICT we have recruited participants along the colorectal carcinoma sequence comprising CRC, HR and LR adenomas. Standard operating procedures ensure consistent sample collection and processing within CORSA, providing a high-quality biorepository and clinical database. The herein selected study population was balanced regarding age, gender, and smoking status to reduce non-biological effects during data analysis.

Despite the fact that major molecular events underlie CRC progression, we did not reveal any significant metabolic difference between histologically confirmed HR and LR adenomas. Our data suggests that HR and LR adenomas might display similar metabolic patterns. To our knowledge, no previous plasma metabolomics studies have reported on significant variations to discern histologically different adenomas on the metabolome level. Hence, changes in metabolite levels at the premalignant adenomas stage compared to CRC should be subject of further investigations.

A limitation of this study is the lack of detailed dietary information. Diet, amongst other factors, plays a role in some of the pathways such as the tryptophan or caffeine metabolism. Metabolite annotation in untargeted metabolomics is still challenging and many signals associated with CRC remain unidentified. Of note, pathway analyses are usually not fully comprehensive and complete per se, but assist in forming new hypotheses and estimating pathway level differences based on generated metabolomics data. Conflicting results in metabolite levels reported from various studies might arise from different study populations, sample collection and preparation, analytical platforms and statistical approaches applied [15]. To date, no clear recommendation on standardizing metabolomics analyses has been released. Despite the large number of metabolites detected, we must acknowledge that a single analytical method cannot measure the entire plasma metabolome and potential drivers of CRC may have been missed. Of note, LC-MS, as used here, was previously described as one of the leading analytical methods and well suited to study complex human blood samples [13]. A limiting factor in metabolomics is feature identification. The conception of untargeted metabolomics involves a comprehensive, hypothesis generating study acquiring data for as many species as possible, annotating metabolites, and reviewing both known and unknown metabolic changes. In contrast, targeted metabolomics focuses on quantification of a limited number of known metabolites. The metabolites identified in this study have previously yet inconsistently been identified as potential biomarkers of interest in association with CRC. Independent validation using predictive models in other cohorts and confirmation of identity by targeted metabolomics would be needed to evaluate the detected associations and to verify the biomarker potential of the herein described metabolites.

Our untargeted metabolomics approach reveals major differences in plasma metabolic features in patients with CRC compared to HR and LR adenomas and might provide substantial information towards a more detailed picture of CRC metabolic pathway networks.

## 4. Materials and Methods

### 4.1. Study Population

Within the MetaboCCC consortium, a large consortium of four independent European CRC cohorts, we analyzed untargeted metabolomics data from the Austrian CORSA. CORSA is an ongoing multicenter study recruiting participants in cooperation with the province-wide CRC screening program B-PREDICT using a FIT as an initial screening. FIT-positive tested participants received a complete colonoscopy and were invited to take part in CORSA. Additional participants were recruited at four hospitals in Vienna. CORSA includes men and women aged between 30 and 90, and excludes patients diagnosed with hereditary CRC syndromes, with any previous cancer history or with inflammatory bowel diseases, such as Crohn’s disease, ulcerative colitis or diverticulitis. EDTA plasma samples and written informed consent were obtained from all study participants. Information on demographic (e.g., age at diagnosis, weight, height) and lifestyle factors (including diabetes status, alcohol consumption, and smoking status) was obtained through self-assessment using the basic CORSA questionnaire. Clinical data were abstracted from medical records, and adenoma samples were categorized according to their size and histopathological finding. All CRC patients were diagnosed as histologically confirmed, sporadic CRC, stage I-IV. Adenomatous tubular polyps > 1 cm, adenomatous tubulo-villous polyps and adenomatous villous polyps were considered HR adenomas. LR adenomas were defined as adenomatous tubular polyps < 1 cm or hyperplastic polyps. The presented analysis was performed with data from patients diagnosed with CRC (*n* = 88), LR adenomas (*n* = 200), and HR adenomas (*n* = 200) selected from the CORSA biobank. CRC and adenoma samples were matched for age, sex, and smoking status. Plasma samples were obtained from participants prior to surgery or any radio- or chemotherapy.

### 4.2. Biospecimen Handling, Metabolomics Analysis, and Data Pre-Processing

Blood samples were processed within 4 h following standardized protocols and stored at −80 °C before shipping on dry ice to the International Agency for Research on Cancer (IARC) in Lyon, France, for analysis. Untargeted metabolomics analyses were performed using ultra-high performance liquid chromatography-quadrupole time-of-flight mass spectrometry (UHPLC-qTOF-MS). Details on the sample preparation and analysis have been previously described by Geijsen and Brezina et al. [19]. Briefly, samples derived from the CORSA cohort were blinded, and randomly distributed into two analytical batches. CRC, HR as well as LR adenoma samples were equally distributed across the two batches. In this study, we define chromatographic peaks derived from specific ions as “features”, whereas “metabolites” or “compounds” are defined as confirmed molecules consisting of one or more features. Feature finding was performed with the MassHunter software (Agilent Technologies, Santa Clara, CA, USA) using a recursive feature finding workflow as described earlier [19], with the exception that features in the samples used for the present study were aligned by Agilent Mass Profiler Pro 12.5, using retention time and mass windows of 0.07 min and 15 ppm + 2 mDa, respectively. Features present in every blank sample within at least one batch were excluded, unless they were more than 5-fold greater in intensity in samples than in blanks within the same batch (based on fold-change analysis in Mass Profiler Pro). Chromatographic peak areas were used as a measurement of feature intensity.

### 4.3. Feature Identification

Features were clustered according to retention time, mass, and intensity correlation across samples to facilitate finding those originating from the same compound. *m*/*z* values were searched against the human metabolite database (HMDB, https://hmdb.ca/, accessed on 21 July 2020) [49] using [M + H]^+^, [M − H_2_O + H]^+^, and [M + Na]^+^ ions, with 15 ppm molecular weight tolerance. In addition, an in-house compound and feature database at IARC was searched for features with matching retention time and mass. Identification was confirmed by reanalysis of representative study samples and authentic chemical standards when available, and comparison of the retention times and the fragmentation (MS/MS) spectra. Supplementary File S4 is given a detailed overview of the retention times MS/MS spectra of all identified metabolites and the corresponding authentic chemical standards. When standards were not available, MS/MS spectra were acquired when possible and compared against those in mzCloud (www.mzcloud.org, last access: 13 December 2020) or Metlin (https://metlin.scripps.edu, accessed between 14 January and 4 February 2021 [50]. The level of identification was as proposed by Sumner et al. [51]. If two or more metabolic features were assigned to the same metabolite identification, we selected the ion [M + H]^+^ or [M]^+^ as the a representative feature with the highest intensity.

### 4.4. Metabolic Enrichment and Pathway Analysis

Enrichment and pathway analyses were performed via the web server of MetaboAnalyst (version 5.0, www.metaboanalyst.ca/, last access: 8 February 2021)) to depict the most relevant metabolic pathways involving the identified features of the untargeted metabolomics dataset [52]. The summary plot of the metabolite set enrichment analysis was implemented using hypergeometric testing to evaluate whether a particular metabolite set was represented more than expected by chance within the provided compound list. One-tailored *p*-values were provided after adjusting for multiple testing (Holm-Bonferroni method). The pathway analysis module offers two different parameters to determine relevant pathways within the comparison groups: the statistical *p*-values derived from the quantitative enrichment analysis, and the pathway impact value calculated by the topological analysis with the relative-betweenness centrality. Here, we calculated metabolic pathways using Fisher’s Exact Test and relative-betweenness centrality based on the KEGG pathway library.

### 4.5. Statistical Analysis

Features with >50% missing values in all study groups were excluded from further analysis. Separate multivariable logistic regression models were estimated to test the association between the intensity of each feature and disease state of three case-control comparison groups (CRC against HR and LR, CRC vs. HR, and CRC vs. LR adenomas) adjusted for age at diagnosis, sex, BMI, and smoking status. Disease state was treated as dependent variable. Feature intensity was entered as main explanatory variable into the model after log-transformation and adjustment for batch. To evaluate any potential influence of advanced stage CRC on the main analysis, a sensitivity analysis was performed. BMI is defined as weight in kilograms divided per the square of height in meters (kg/m^2^). Smoking status was categorized as current, former, and never smoker. Standardized ORs (OR.std) and corresponding 95% CIs were derived for estimated coefficients of log-intensities. OR.std represents the change in CRC occurrence when there is a one standard deviation (SD) change in log metabolite intensity, allowing comparisons of effect sizes between different features. SDs for standardization were derived from control groups. Features showing FDR-adjusted *p*-values < 0.05 were carried forward for identification. Statistical analyses were performed using R (version 4.0.2, R Foundation for Statistical Computing, Vienna, Austria, URL https://www.R-project.org/, accessed on 14 December 2020).

## 5. Conclusions

Data obtained from this study was generated through an untargeted metabolomics approach using plasma samples from the CORSA biobank comprising CRC, HR and LR adenomas. The observed metabolic variations might be reflected by major differences in plasma metabolomes between patients diagnosed with CRC and precancerous adenomas. In general, the herein identified metabolites could be assigned to metabolic pathways essentially involved in sustaining and driving cellular energy. The nicotinate and nicotinamide pathway plays vital roles in energy metabolism of eukaryotic cells by serving as a precursor of NAD^+^ synthesis. Lipid metabolism involves the carnitine, linoleic acid, and glycerophospholipid pathways. Peptide and protein synthesis is driven by amino acids like proline and valine. Bile acids such as taurine as well as bacterial tryptophan metabolites point towards a role of the gut microbiome in CRC. Our study provides potential towards studying the metabolic puzzle of CRC and offers yet unidentified metabolites for future investigations.

## Figures and Tables

**Table 1:** Demographics and clinical characteristics of the study population (*n* = 488).

	CRC	HR ^a^	LR ^b^
Number of Participants	88	200	200
**Gender**			
Male *n(%)*	60 (68.2)	132 (66.0)	132 (66.0)
**Age (years)**			
Median *(IQR)*	70.0 (60.0–76.0)	65.4 (56.4–72.6)	66.0 (55.3–72.9)
**Body Mass Index (kg/m^2^)**			
Median *(IQR)*	26.1 (23.8–29.4)	27.3 (24.3–30.0)	27.2 (24.6–30.9)
Underweight < 18.5 *n(%)*	0 (0)	3 (1.5)	1 (0.5)
Normal weight 18.5–24.9 *n(%)*	26 (29.5)	57 (28.5)	51 (25.5)
Overweight 25–29.9 *n(%)*	38 (43.2)	82 (41.0)	81 (40.5)
Obese ≥ 30 *n(%)*	15 (17.0)	48 (24.0)	61 (30.5)
Missing	9 (10.2)	10 (5.0)	6 (3.0)
**Smoking status *n(%)***			
Current	20 (22.7)	50 (25.0)	35 (17.5)
Former	30 (34.2)	55 (27.5)	60 (30.0)
Never	35 (39.7)	91 (45.5)	97 (48.5)
Missing	3 (3.4)	4 (2.0)	8 (4.0)
**Site *n(%)*^c^**			
Colon - distal	21 (23.9)	-	-
Colon - proximal	33 (37.5)	-	-
Rectum	34 (38.6)	-	-
**CRC stage *n(%)*^d^**			
I	30 (34.1)	-	-
II	17 (19.3)	-	-
III	18 (20.5)	-	-
IV	12 (13.6)	-	-
Unspecified	3 (3.4)	-	-
Missing	8 (9.1)	-	-
**Histopathology of polyps *n(%)*^e^**			
Hyperplastic	-	-	11 (5.5)
Tubular < 1 cm	-	-	189 (94.5)
Tubular > 1 cm	-	64 (32.0)	-
Tubulo-villous	-	128 (64.0)	-
Villous	-	8 (4.0)	-

**^a^** HR: high-risk adenomas. **^b^** LR: low-risk adenomas (adenomas are classified according to the most severe finding). **^c^** Localization of CRC provided from clinical records. Distal colon: sigmoid colon, descending colon, splenic flexure. Proximal colon: transverse colon, hepatic flexure, ascending colon, cecum, appendix. Rectum: rectum, rectosigmoid junction. **^d^** UICC stage based on the TNM Classification of Malignant Tumors. **^e^** Histopathology of polyps. HR adenomas: adenomatous tubular polyps > 1 cm, tubulo-villous polyps, and villous polyps. LR adenomas: adenomatous tubular polyps < 1 cm or hyperplastic polyps.

**Table 2:** Quantification of FDR-adjusted *p*-values of statistically significant metabolic features within the three comparison groups.

	CRC vs. HR + LR	CRC vs. HR	CRC vs. LR
Sum of Statistically Significant Features	409	367	384
***q*-value ^a^**			
5.0 × 10^−2^–1.0 × 10^−2^	103	101	85
1.0 × 10^−2^–1.0 × 10^−3^	78	86	97
1.0 × 10^−3^–1.0 × 10^−5^	122	131	99
1.0 × 10^−5^–1.0 × 10^−10^	92	43	88
1.0 × 10^−10^–1.0 × 10^−20^	11	6	15
<1.0 × 10^−20^	3	0	0

**^a^***q*-value: FDR-corrected *p*-value.

**Table 3:** List of the identified metabolites and respective pathways when comparing CRC against HR and LR adenomas.

Pathway and Metabolite Name	RT ^a^	*m*/*z*^b^	ID Level ^c^	*q*-Value ^d^	OR [CI.Low; CI.Up] ^e^
**Nicotinate and nicotinamide metabolism**					
1-methylnicotinamide	0.59	137.0711	1	9.46 × 10^−9^	0.20 [0.12; 0.34]
**Carnitine pathway**					
Carnitine	0.59	162.1132	1	1.15 × 10^−2^	0.22 [0.08; 0.61]
Tetradecanoylcarnitine (C14:0)	5.99	372.3109	1	1.16 × 10^−4^	0.25 [0.13; 0.48]
Tetradecenoylcarnitine (C14:1)	5.83	370.2959	2	1.46 × 10^−5^	0.38 [0.25; 0.57]
Tetradecadiencarnitine (C14:2)	5.62	368.2799	2	5.02 × 10^−5^	0.39 [0.25; 0.59]
Hexanoylcarnitine (C6:0)	3.33	260.1855	2	1.86 × 10^−3^	0.42 [0.25; 0.68]
Hexadecenoylcarnitine (C16:1)	6.10	398.3263	2	9.27 × 10^−7^	0.22 [0.12; 0.39]
Hexadecadienoylcarnitine (C16:2)	5.93	396.3105	2	7.41 × 10^−5^	0.29 [0.16; 0.51]
Octanoylcarnitine (C8:0)	4.42	288.2177	2	9.86 × 10^−4^	0.46 [0.3; 0.7]
Decanoylcarnitine (C10:0)	5.13	316.2495	1	7.29 × 10^−3^	0.53 [0.35; 0.8]
Decenoylcarnitine (C10:1) isomer 2	4.96	314.2327	2	7.58 × 10^−4^	0.35 [0.2; 0.61]
Decenoylcarnitine (C10:1) isomer 1	4.87	314.2328	2	1.61 × 10^−4^	0.44 [0.27; 0.7]
Dodecanoylcarnitine (C12:0)	5.64	344.2804	1	9.34 × 10^−5^	0.37 [0.23; 0.58]
Dodecenoylcarnitine (C12:1)	5.50	342.2638	2	4.40 × 10^−4^	0.33 [0.19; 0.58]
Propionylcarnitine (C3:0)	1.32	218.1382	1	2.15 × 10^−5^	5.14 [2.56; 10.68]
**Bilirubin pathway**					
Bilirubin	7.93	583.2554	1	2.53 × 10^−4^	0.46 [0.31; 0.67]
Bilirubin isomer 2	5.11	585.2696	2	4.90 × 10^−7^	0.33 [0.21; 0.5]
Bilirubin isomer 1	4.31	585.2685	2	8.43 × 10^−3^	0.46 [0.27; 0.77]
**Bile acid metabolism**					
Taurine	0.63	126.0219	1	6.10 × 10^−13^	16.17 [7.81; 35.24]
Glycochenodeoxycholic acid	6.44	450.3216	1	1.37 × 10^−2^	1.46 [1.13; 1.9]
**Caffeine pathway**					
Caffeine	3.19	195.0884	1	2.14 × 10^−3^	1.28 [1.11; 1.49]
Theobromine	2.38	181.0721	1	8.42 × 10^−3^	1.46 [1.14; 1.89]
Theophylline	2.81	181.0723	1	4.20 × 10^−2^	1.33 [1.05; 1.71]
**Phenolic acid metabolism**					
Hippuric acid	3.07	180.0657	1	6.52 × 10^−21^	3.15 [2.46; 4.13]
**Nucleotide metabolism**					
Hypoxanthine	1.16	137.0456	1	7.60 × 10^−3^	2.14 [1.3; 3.59]
**Tryptophan pathway**					
Indoleacetic acid	4.13	176.0716	1	1.17 × 10^−10^	4.23 [2.77; 6.68]
Indole-3-propionic acid	4.56	190.0870	1	1.19 × 10^−12^	2.57 [1.99; 3.37]
Indolelactic acid	3.83	206.0823	1	2.70 × 10^−3^	3.06 [1.59; 5.98]
**Indole**					
Isatin	3.31	148.0394	1	7.34 × 10^−12^	5.01 [3.2; 8.09]
**Linoleic acid and glycerophospholipid metabolism**					
LysoPC (14:0) isomer 2	6.73	468.3076	2	3.02 × 10^−2^	0.55 [0.34; 0.88]
LysoPC (15:0)	6.88	482.3230	2	1.98 × 10^−2^	0.47 [0.27; 0.82]
LysoPC (16:0)	7.00	496.3400	2	1.07 × 10^−7^	0.04 [0.01; 0.12]
LysoPC (16:1)	6.82	494.3243	2	3.06 × 10^−5^	0.32 [0.19; 0.52]
LysoPC (17:0)	7.13	510.3539	2	2.64 × 10^−3^	0.4 [0.23; 0.68]
LysoPC (18:0)	7.24	524.3713	2	2.89 × 10^−7^	0.15 [0.07; 0.29]
LysoPC (18:1)	7.06	522.3557	2	1.62 × 10^−2^	0.34 [0.16; 0.73]
LysoPC (20:4)	6.90	544.3402	2	1.09 × 10^−4^	0.22 [0.11; 0.44]
LysoPC (22:5)	6.97	570.3538	2	1.15 × 10^−2^	0.35 [0.17; 0.71]
LysoPC (22:6)	6.89	568.3390	2	4.92 × 10^−2^	0.48 [0.25; 0.89]
LysoPC (P-16:0)	7.11	480.3475	2	6.50 × 10^−5^	0.23 [0.12; 0.45]
PC (36:4)	8.65	782.5728	2	5.87 × 10^−3^	0.3 [0.14; 0.64]
PC (38:4)	9.21	810.6029	2	1.40 × 10^−3^	0.16 [0.05; 0.44]
**Fatty acid metabolism**					
Docosahexaenoic acid (DHA)	7.23	329.2475	1	7.37 × 10^−3^	0.45 [0.27; 0.75]
**Choline metabolism**					
Choline	0.58	104.108	1	4.67 × 10^−2^	0.26 [0.09; 0.8]
**Valine, leucine and isoleucine biosynthesis**					
Proline	0.69	116.0712	1	5.02 × 10^−3^	3.87 [1.69; 9.12]
Valine	0.80	118.0866	1	2.89 × 10^−2^	0.21 [0.06; 0.69]
**Vitamin E pathway**					
γ-carboxyethyl hydroxychroman	5.27	265.1430	1	3.25 × 10^−2^	2.49 [1.22; 5.1]
**Phenylacetate metabolism**					
Phenylacetylglutamine	3.11	265.1190	1	3.15 × 10^−24^	3.51 [2.71; 4.67]

^a^ RT: retention time in minutes. ^b^ m/z: mass-to-charge ratio. ^c^ ID level: according to the Metabolomics Standards Initiative (MSI). ^d^ *q*-value: FDR-corrected *p*-value. ^e^ OR: Odds ratio, with a one standard deviation change in metabolite intensity representing relative changes in CRC risk. Positive or inverse associations with CRC are characterized by an OR > 1 (red font) or <1 (green font), respectively.

## Data Availability

The data presented in this study are available on request from the corresponding author. As the data analyzed within the present study have been generated within a multi-centre consortium any data sharing have to be approved by all study PIs.

## Supplementary Materials

The following are available online at https://www.mdpi.com/2218-1989/11/2/119/s1. Table S1: Complete list of 442 statistically significant features, Table S2: List of outcome-specific metabolic features, Figure S3: Pathway analysis of the identified metabolites comparing CRC against HR and LR adenomas. File S4: Identification of metabolites. Chromatograms and spectra of study samples and pure chemical standards.
