# Supplementary material for: Untargeted Metabolomics Reveals Major Differences in the Plasma Metabolome between Colorectal Cancer and Colorectal Adenomas
Source: Metabolites. 2021 Feb 19;11(2):119. doi: 10.3390/metabo11020119 (PMC7922413; doi:10.3390/metabo11020119)
Supplement: Supplementary file 1 [file metabolites-11-00119-s001.zip › metabolites-1056311-supple-for conversion/Supplementary Figure S3 revised_proof.docx]

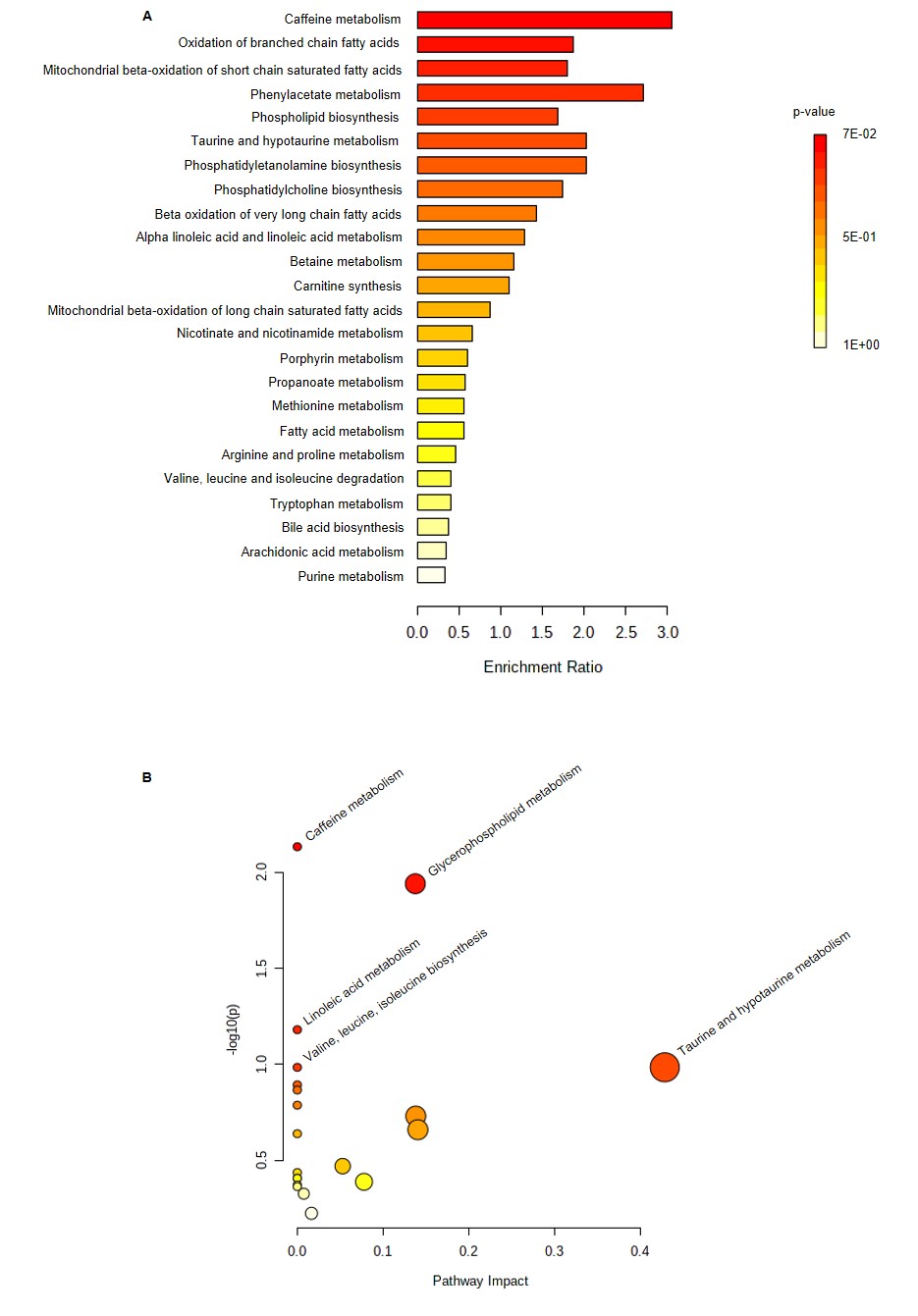


**Supplementary Figure S3.** Pathway analysis of the identified metabolites comparing CRC against HR and LR adenomas. **(A)** Summary plot for over representation analysis. The metabolite set enrichment analysis indicates the most perturbed metabolite patterns. The colors of the bars varying from yellow to red refer to the different p-values calculated by the Holm-Bonferroni method. **(B)** The overview shows all matched pathways according to the p-values from the pathway enrichment analysis and pathway impact values from the pathway topology analysis, with each node representing a specific pathway. Compound colors varying from yellow to red indicate the metabolites are in the data with different levels of significance. All analyses were performed using MetaboAnalyst version 5.0.
