# Supplementary material for: Untargeted Metabolomics Reveals Major Differences in the Plasma Metabolome between Colorectal Cancer and Colorectal Adenomas
Source: Metabolites. 2021 Feb 19;11(2):119. doi: 10.3390/metabo11020119 (PMC7922413; doi:10.3390/metabo11020119)

Supplementary File S4. Identification of metabolites. Chromatograms and spectra of study samples and pure chemical standards.

General notes:

Chromatograms and spectra are from representative study samples and pure chemical standards when available.

All chemical standards were from IROA Technologies' Mass Spectrometry Metabolite Library, except Phenylacetylglutamine (MetaSci), Decanoylcarnitine (Sigma), Glycochenodeoxycholic acid (Sigma), Indole-3-propionic acid (MetaSci), Indoleacetic acid (Sigma), Isatin (Sigma), Propionylcarnitine (Sigma), Tetradecanoylcarnitine (Sigma), and γ-CEHC (Chem Cruz)

- Chromatograms and isotope patterns: Find Compounds by Formula searches in Agilent MassHunter Qualitative Analysis B.06.00 SP1
- Isotope patterns: Red rectangles represent an isotope pattern calculated from the elemental composition (shown in the title of each spectra) , bars inside the rectangles show the observed isotope peaks
- MS/MS spectra: Precursor is indicated with a blue dot above the ion. Collision energy is on top of the spectra, e.g. CID@20.0.

1-Methylnicotinamide

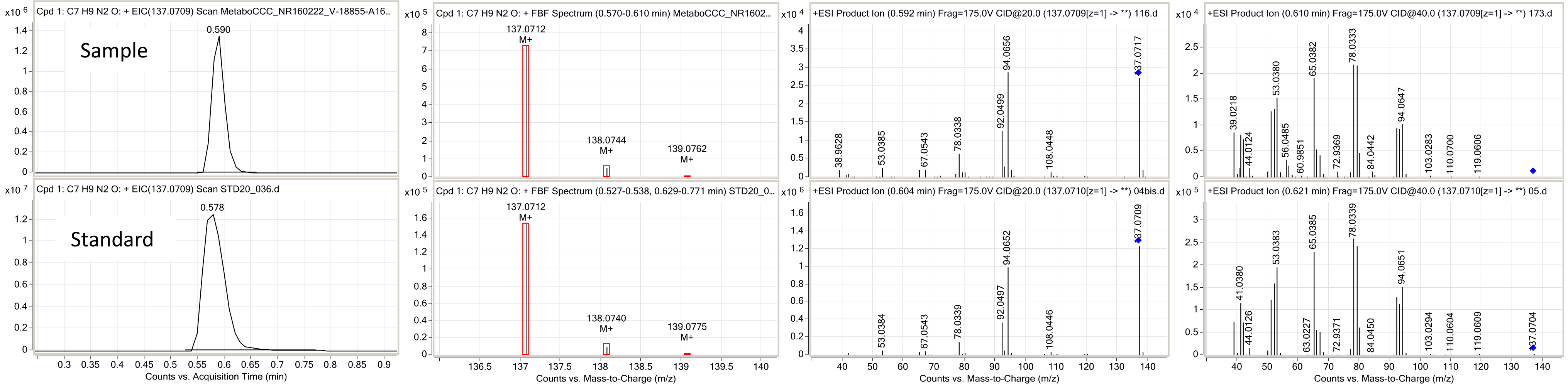

Taurine

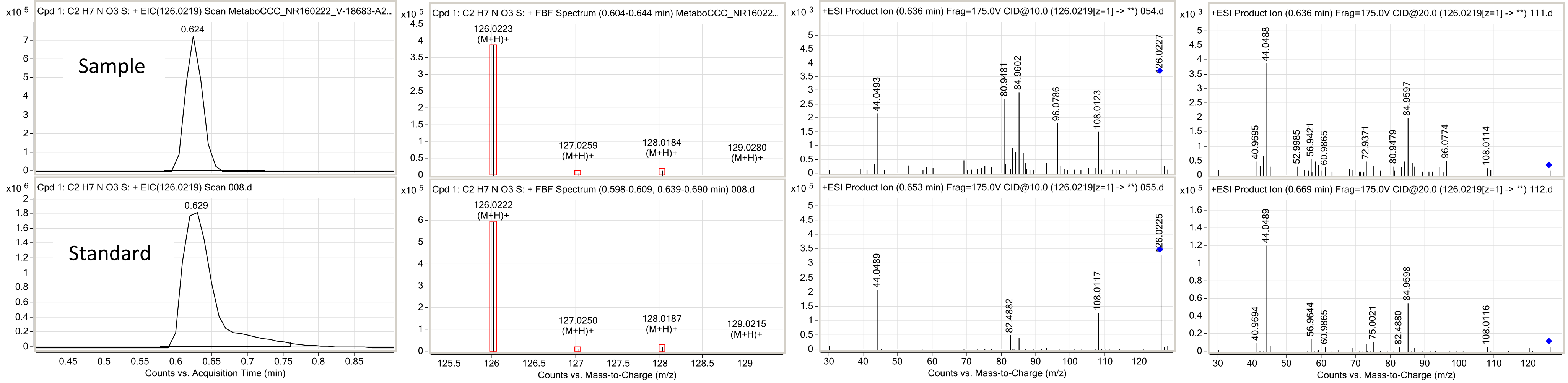

Valine

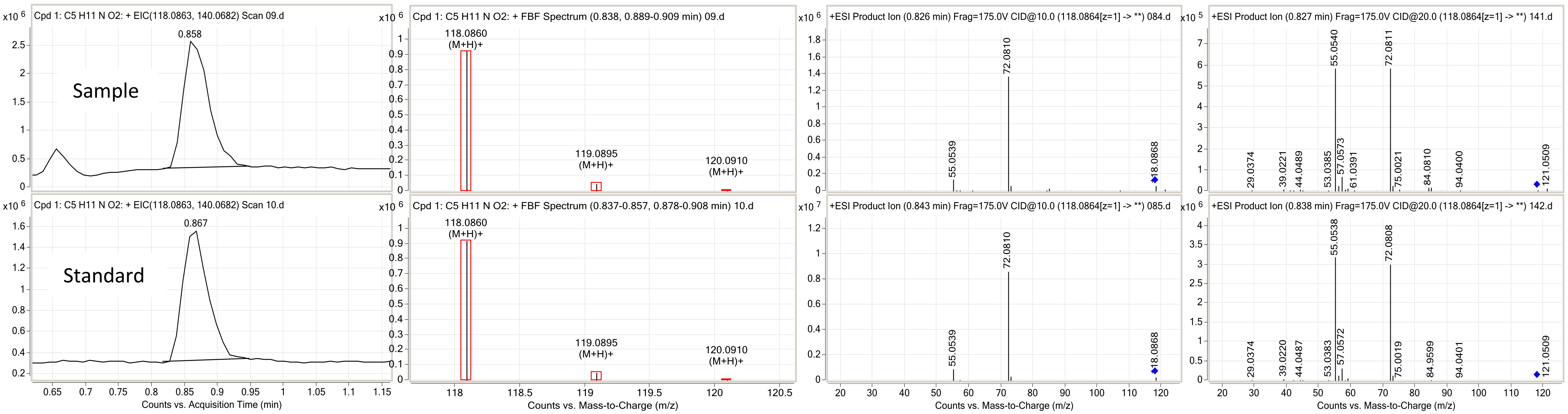

Hypoxanthine

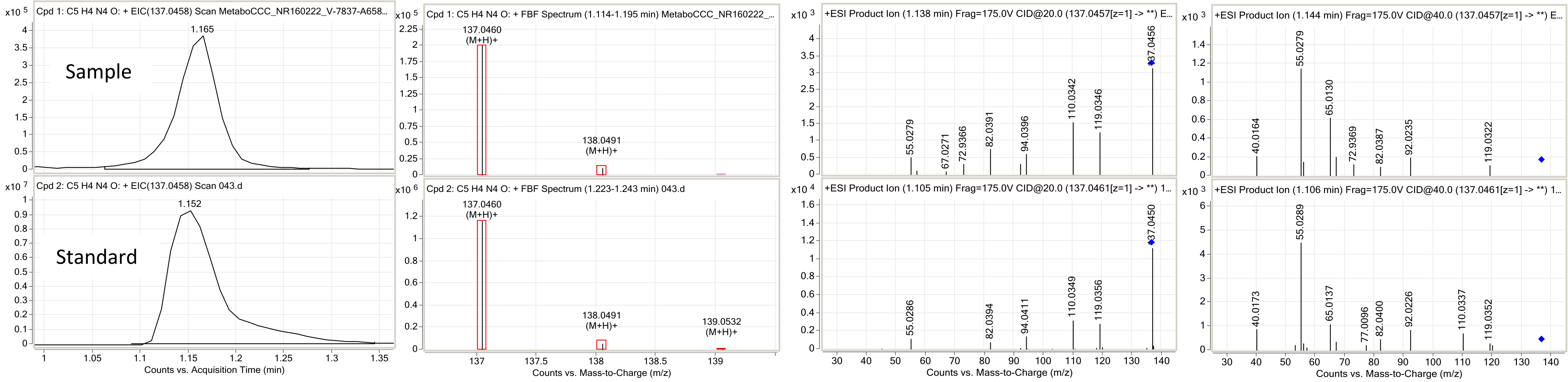

Caffeine

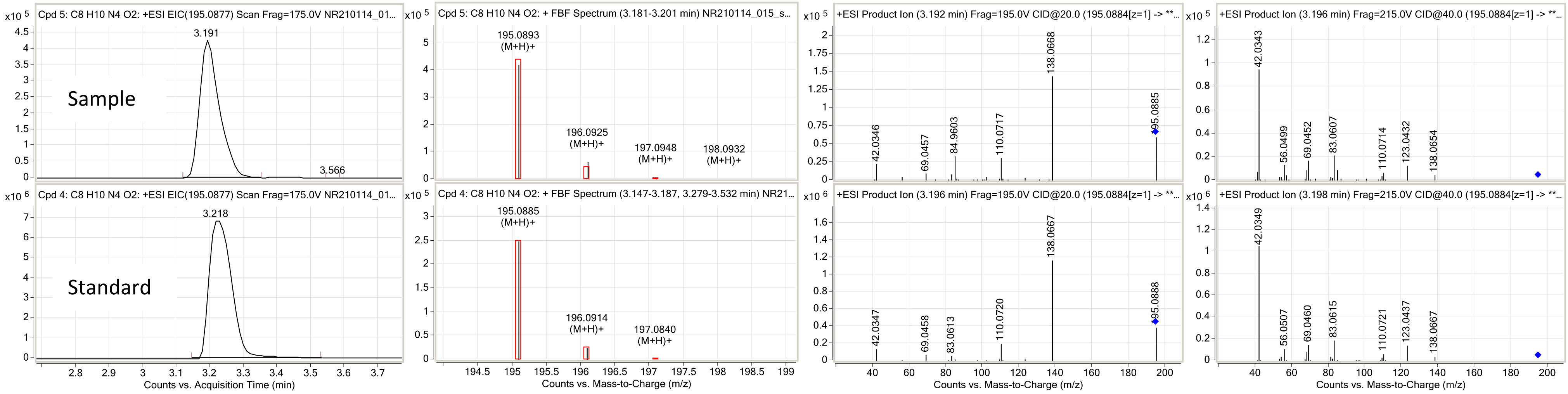

LysoPC (16:1)

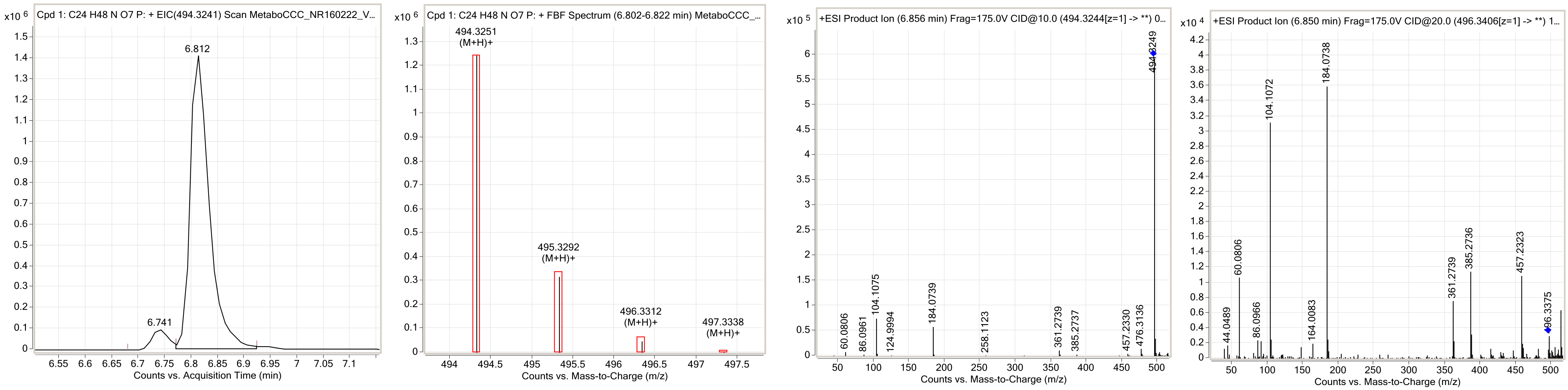

LysoPC (15:0)

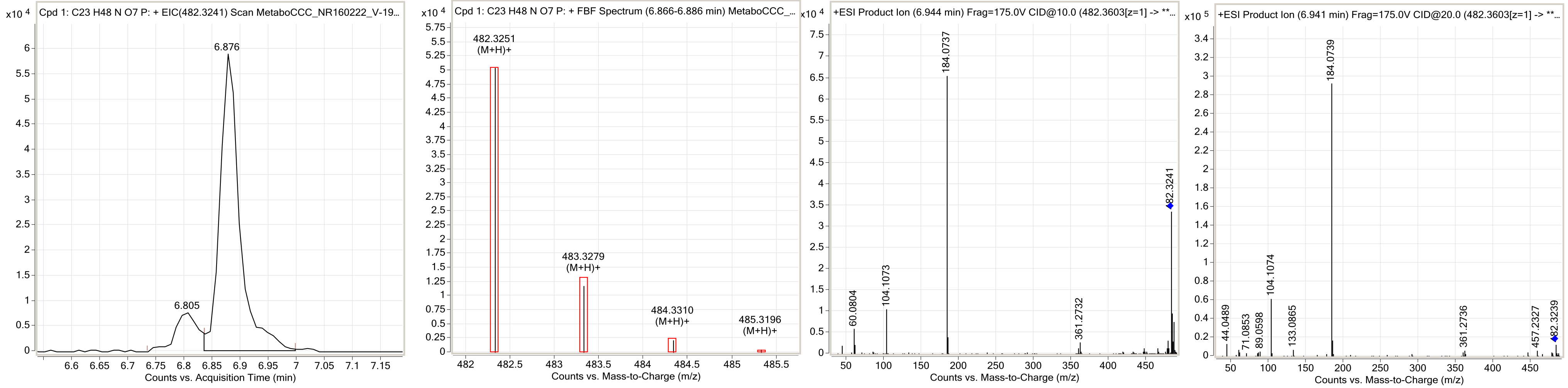

Decanoylcarnitine (C10:0)

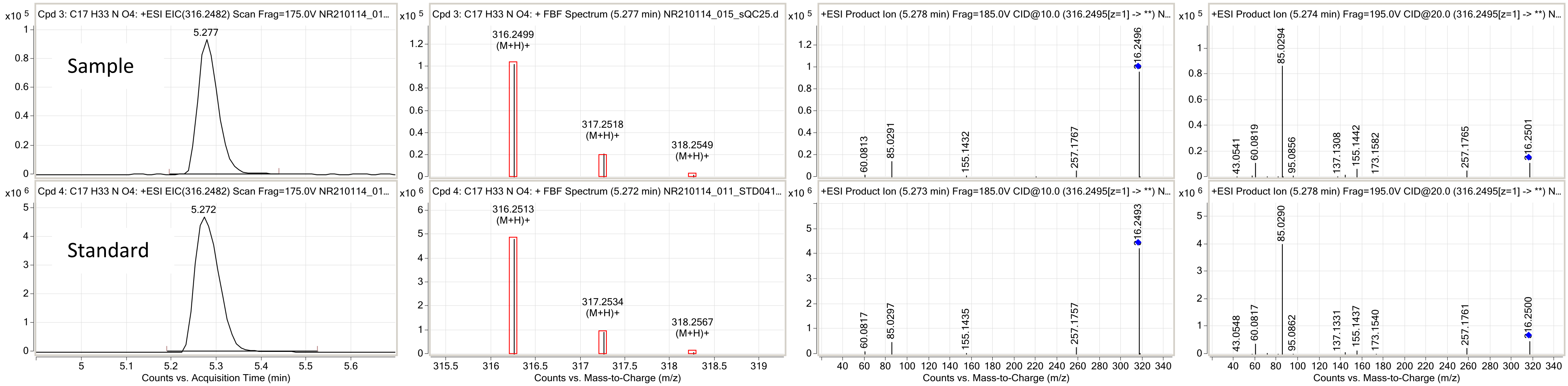

Dodecanoylcarnitine (C12:0)

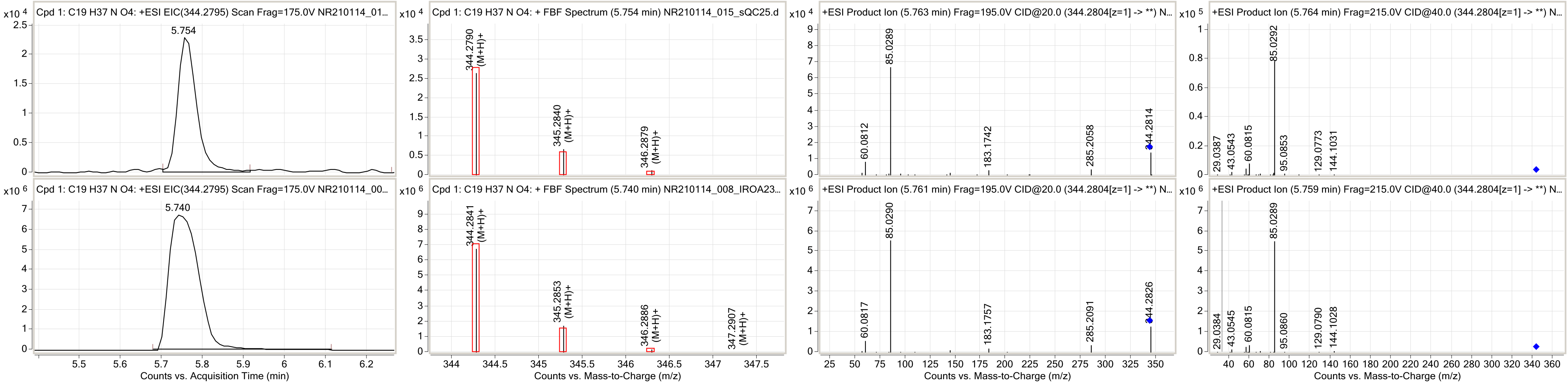

Glycochenodeoxycholic acid

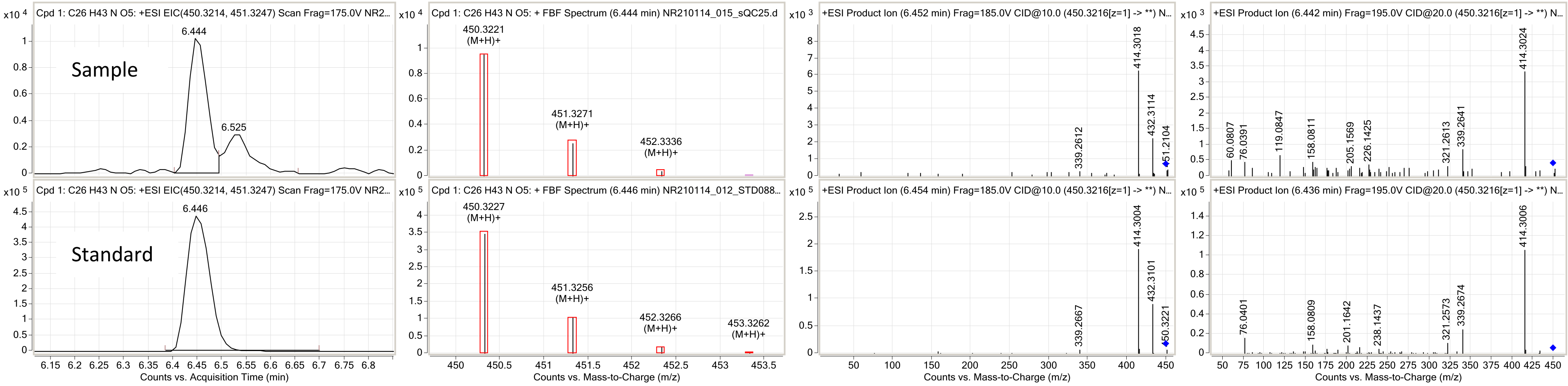

Hippuric acid

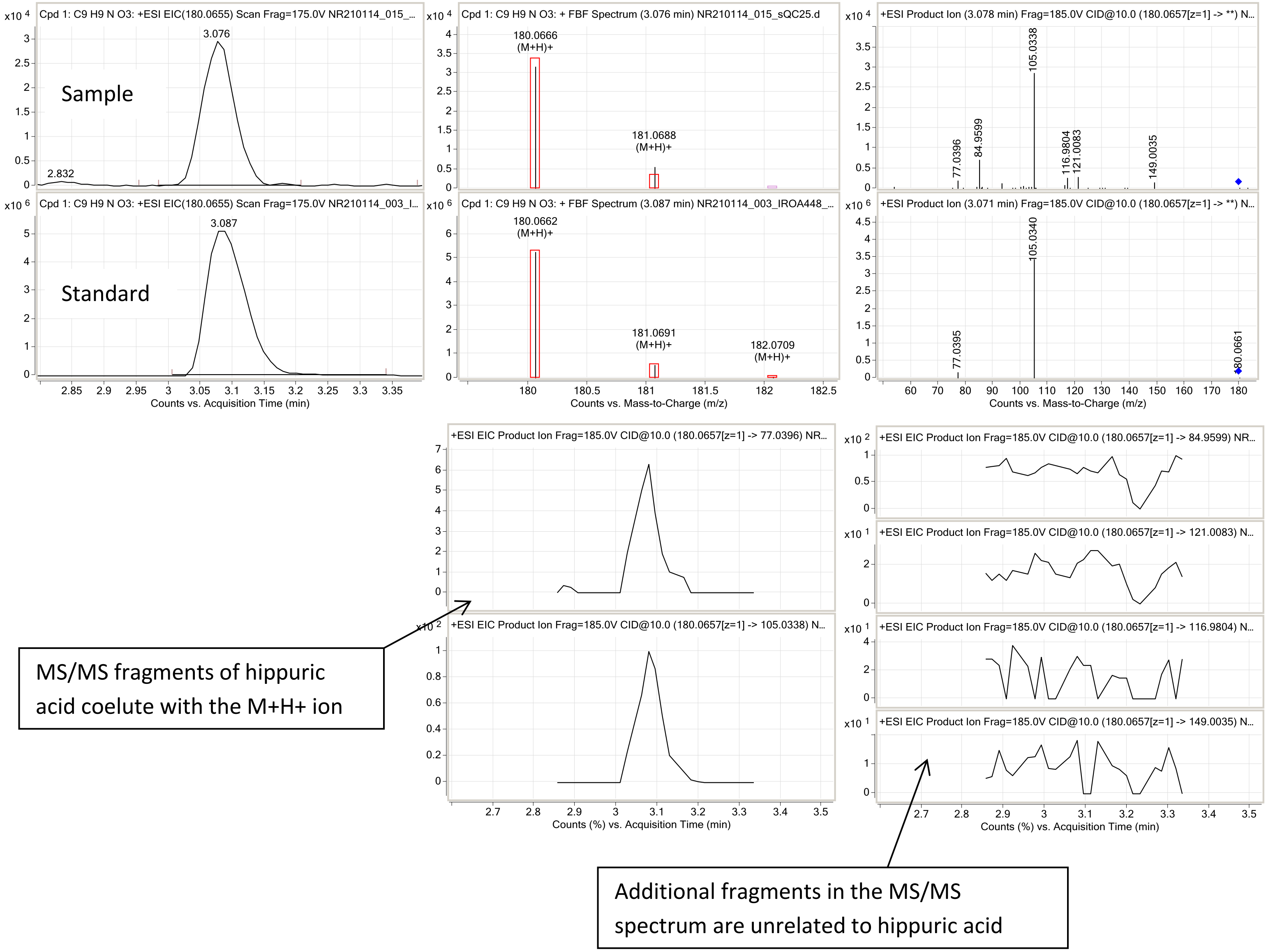

Indoleacetic acid

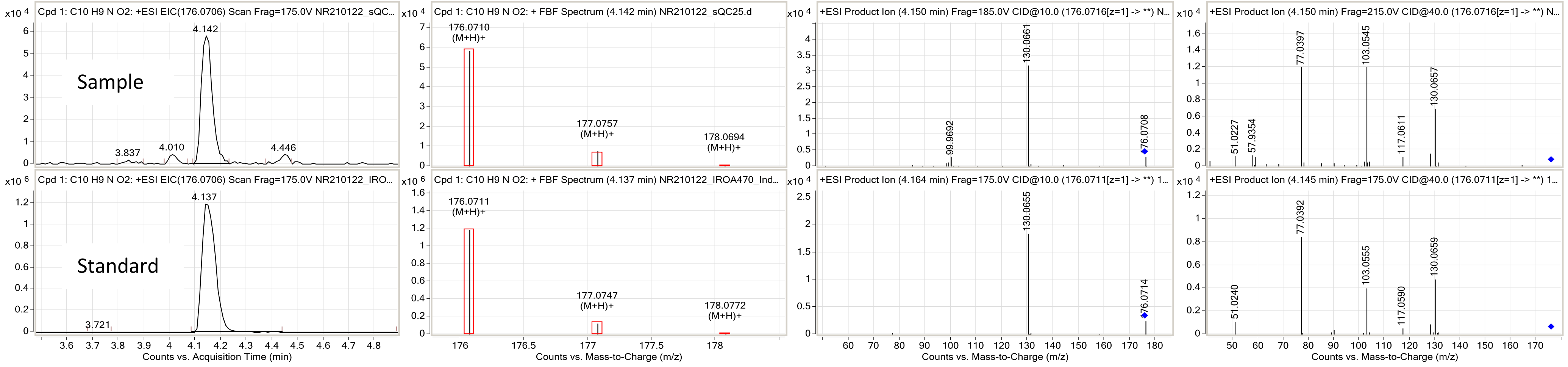

Indole-3-propionic acid

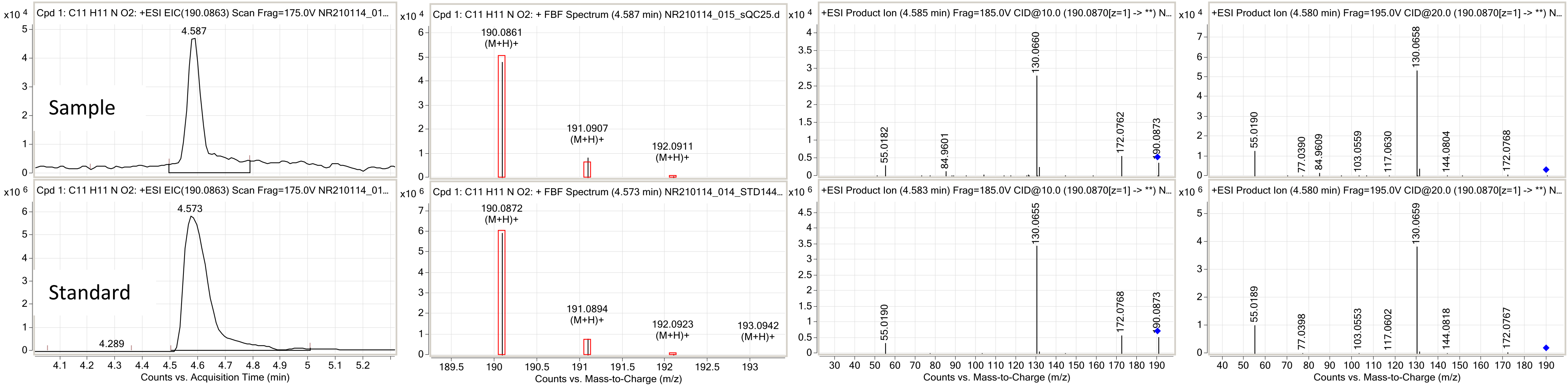

Indolelactic acid

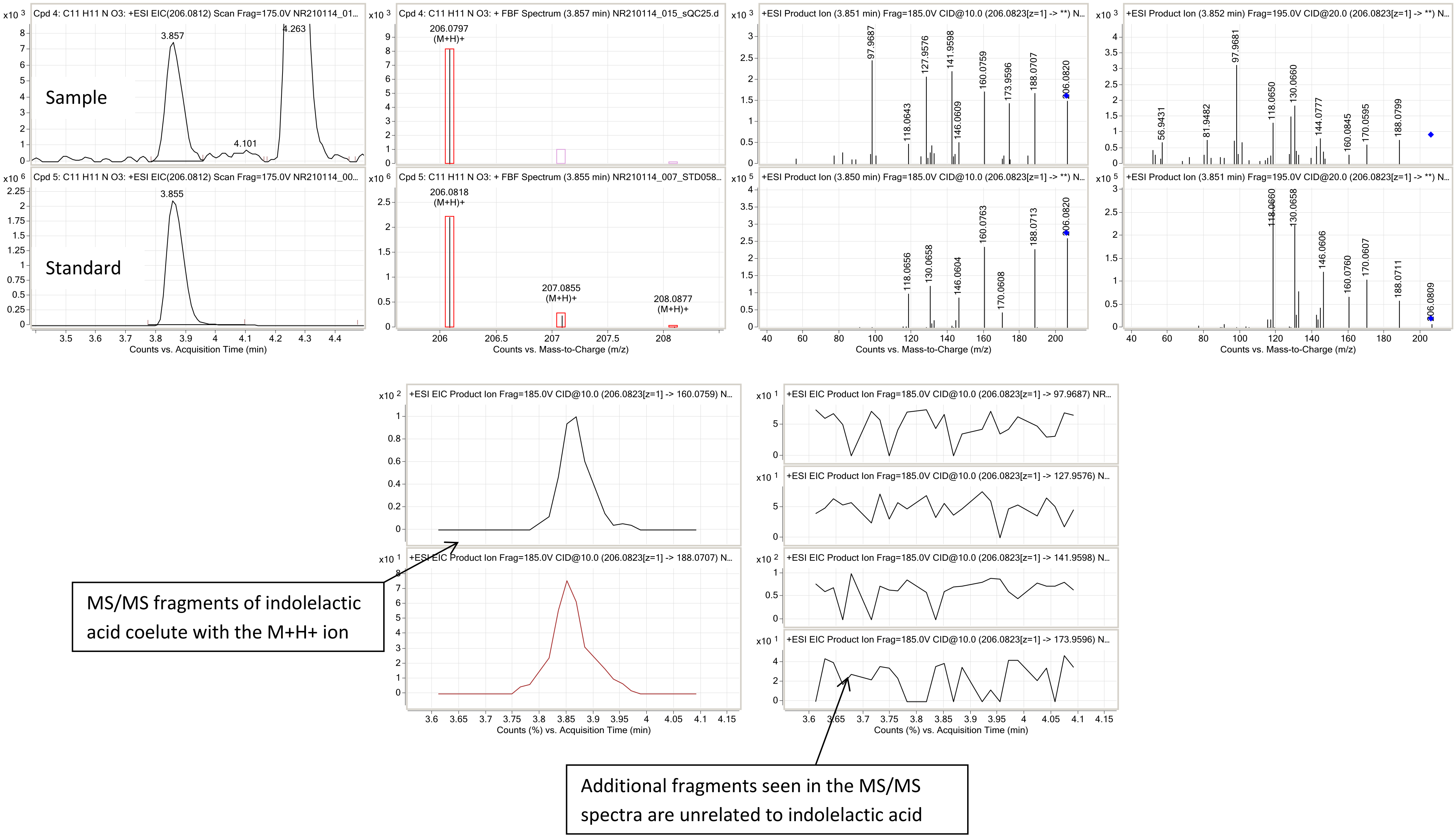

Isatin

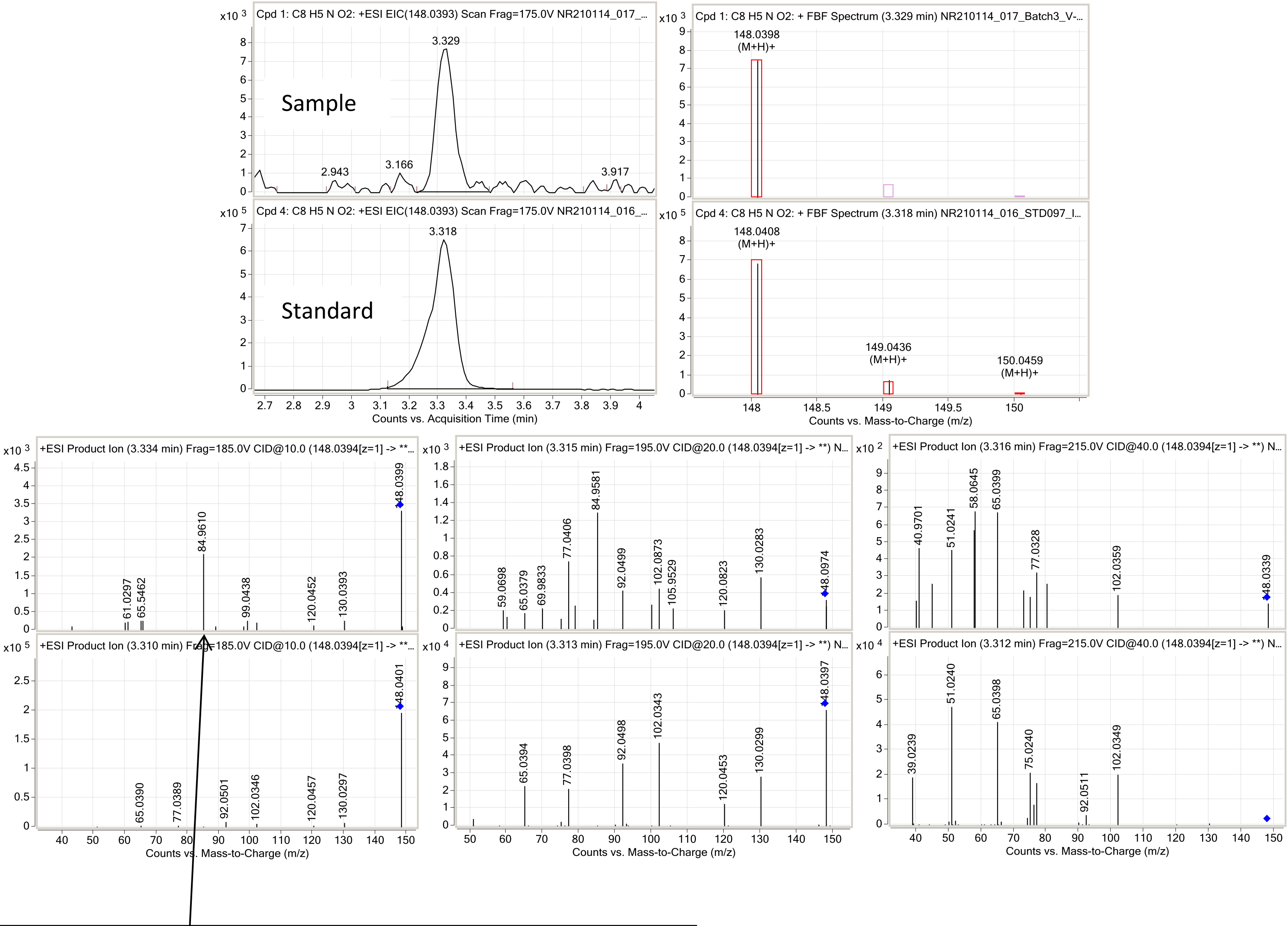

Due to low precursor ion intensity, some unrelated background ions (e.g. *m/z* 84.9610 can be seen (see hippuric acid for details)

Proline

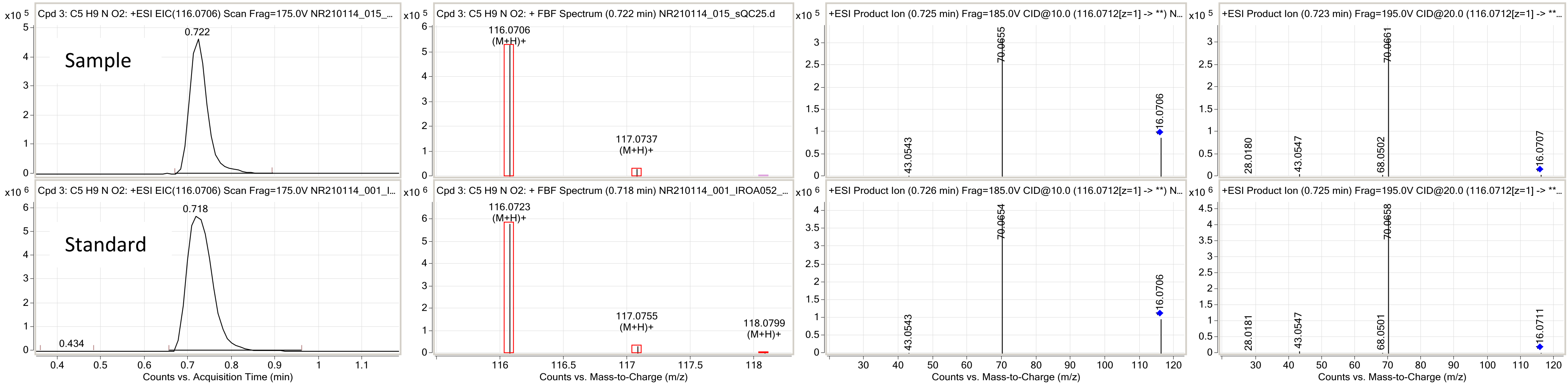

Propionylcarnitine (C3:0)

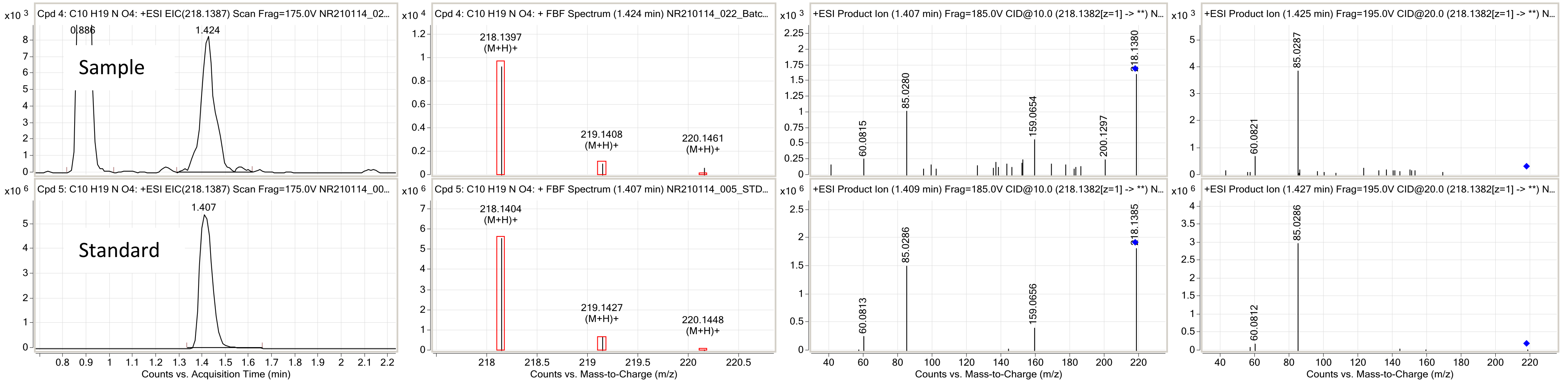

Tetradecanoylcarnitine (C14:0)

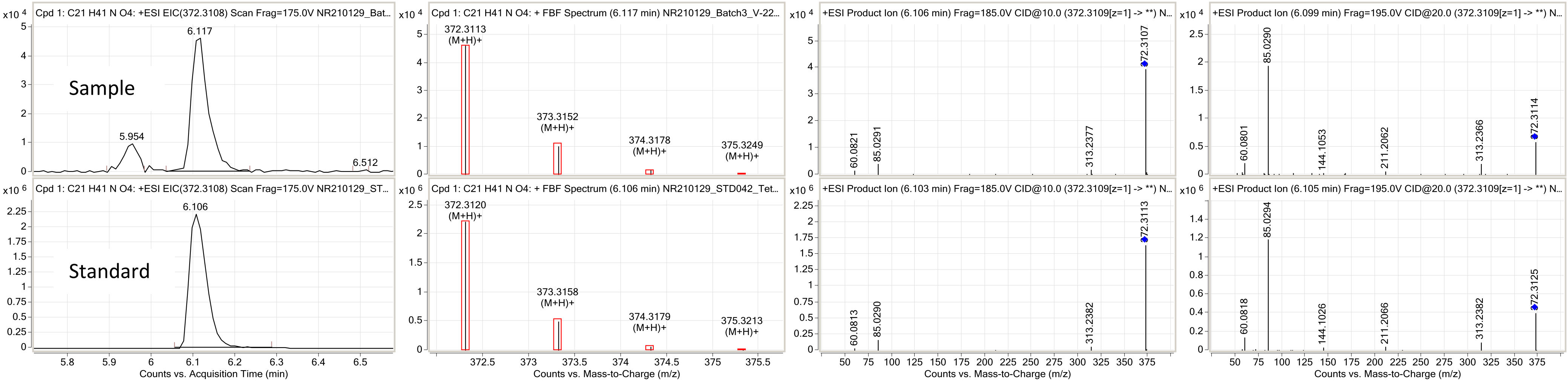

Theobromine

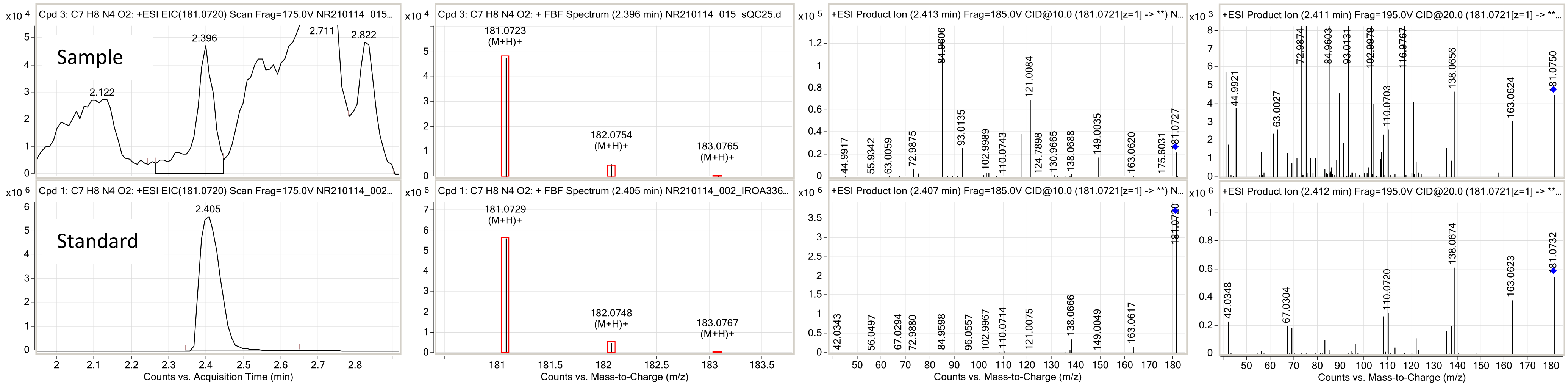

Theophylline

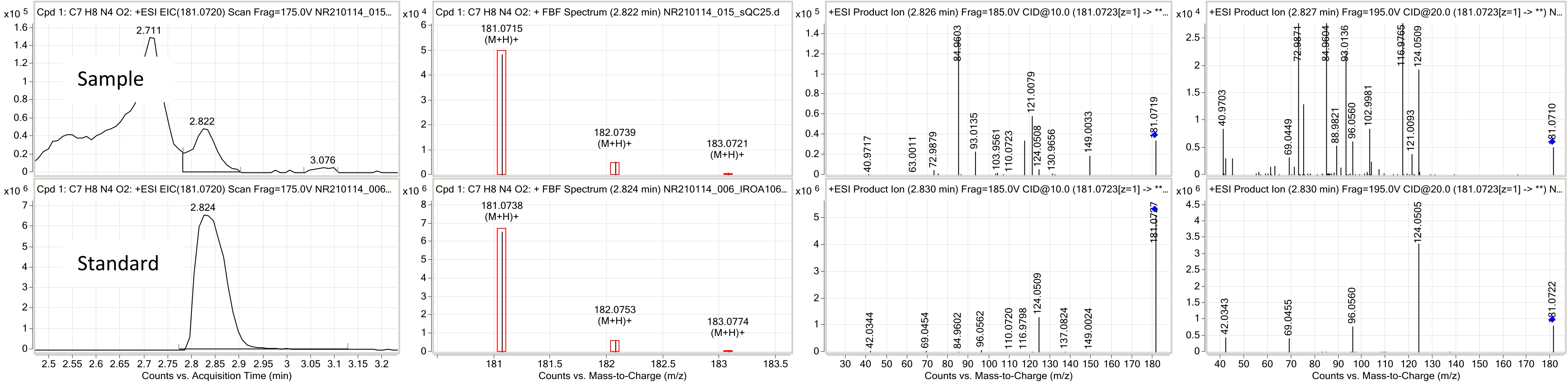

γ-CEHC

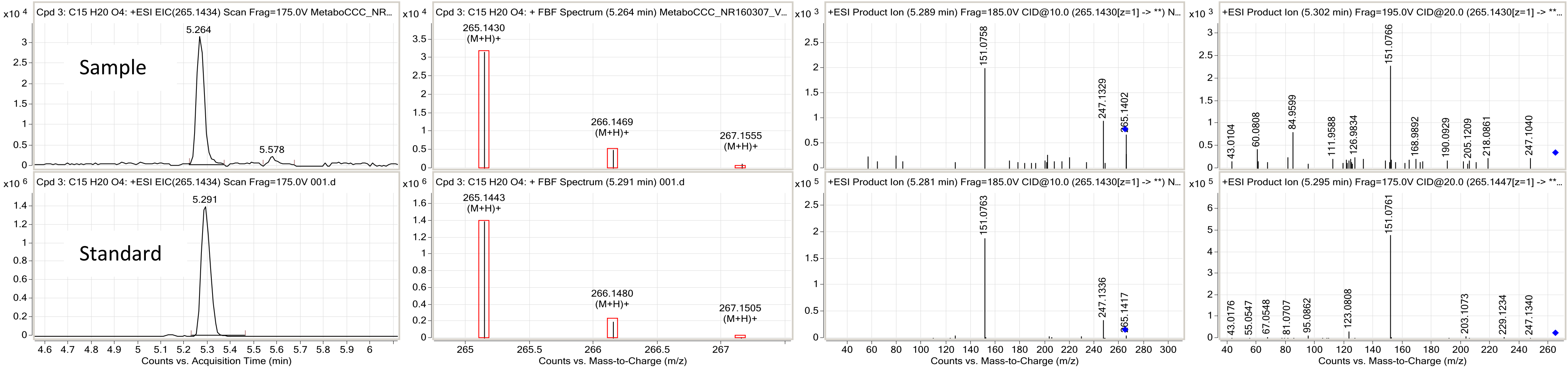

LysoPC (16:0)

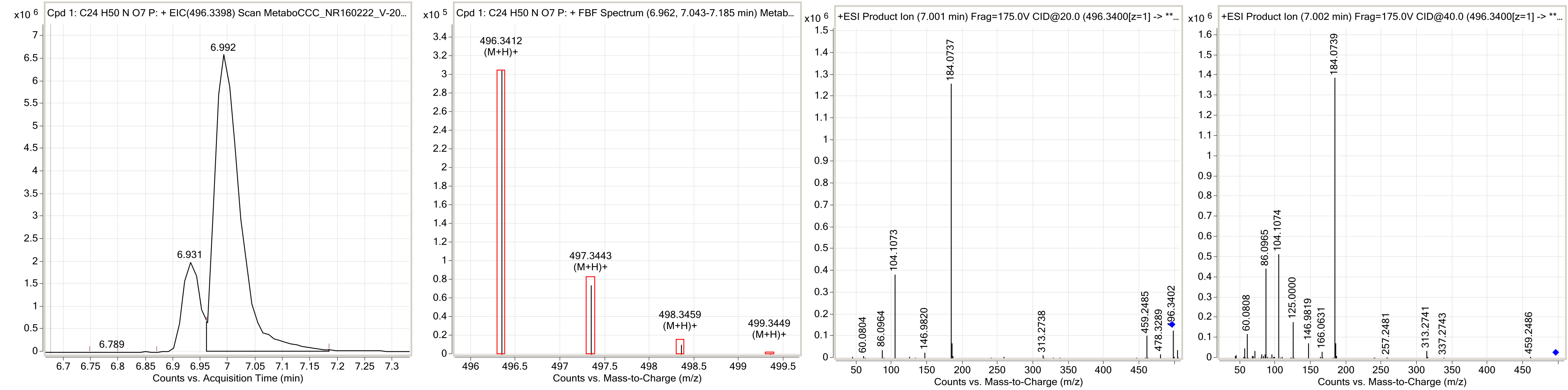

LysoPC (P-16:0)

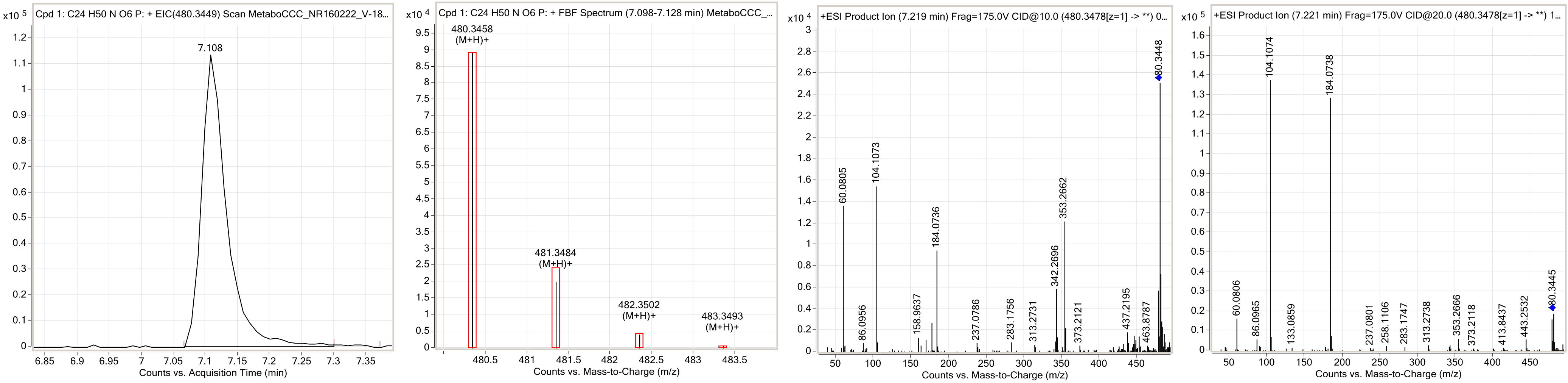

LysoPC (17:0)

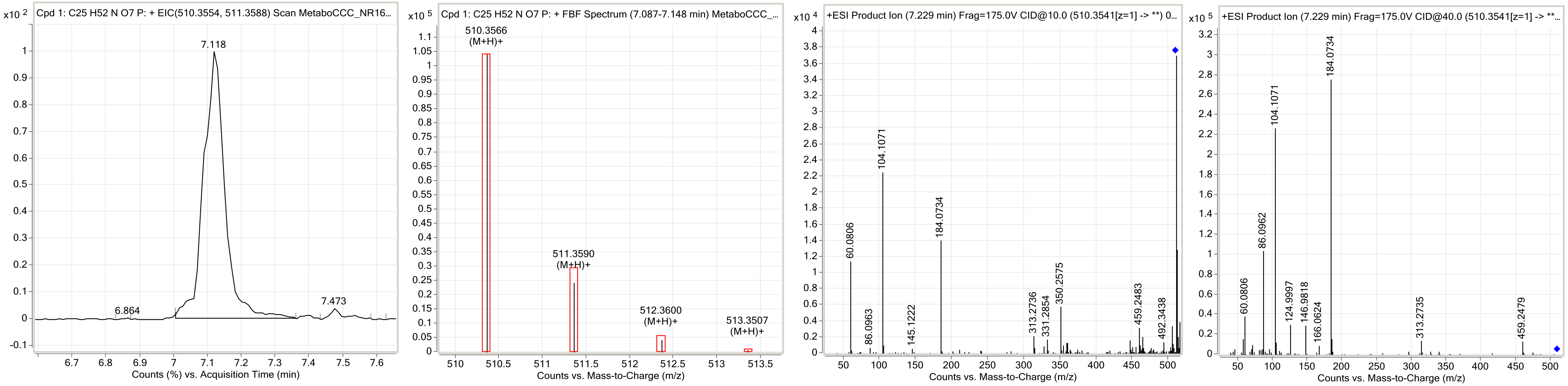

LysoPC (18:0)

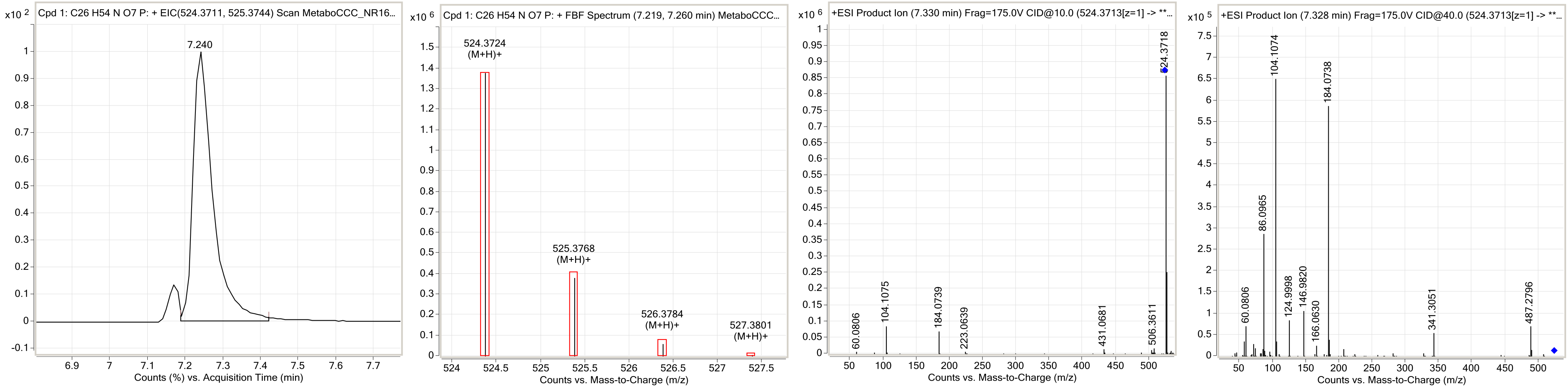

Bilirubin

Bilirubin typically exhibits high number of ions in electrospray (see the spectra below). MS/MS was acquired from $[M+H]^+$

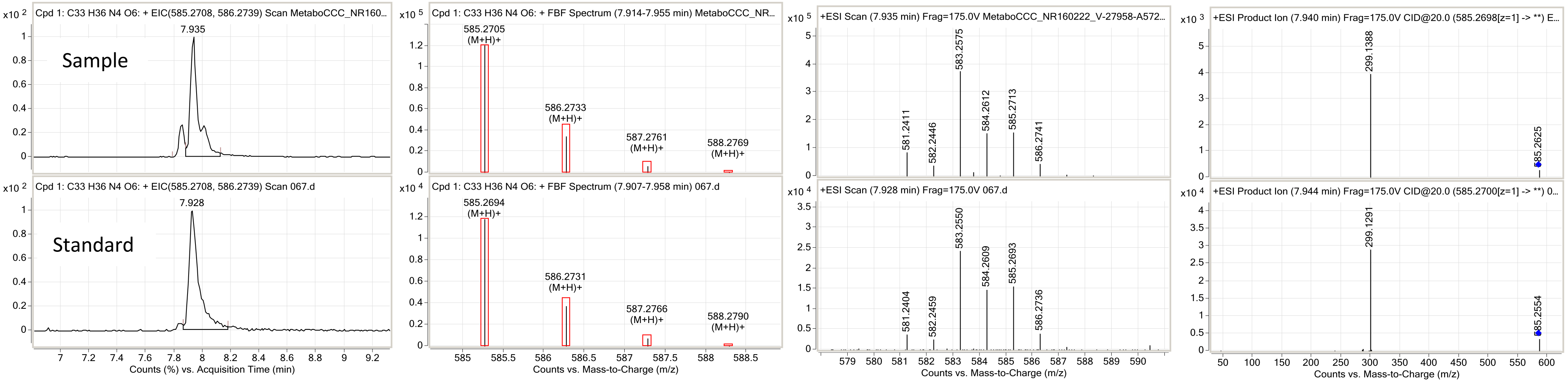

Bilirubin isomers 1 and 2:

Two bilirubin isomers, possible photodegradation products (for details, see Ennever *et al.* J Clin Invest. 1987;79:1674 and Pediatr Res. 1985;19:205)

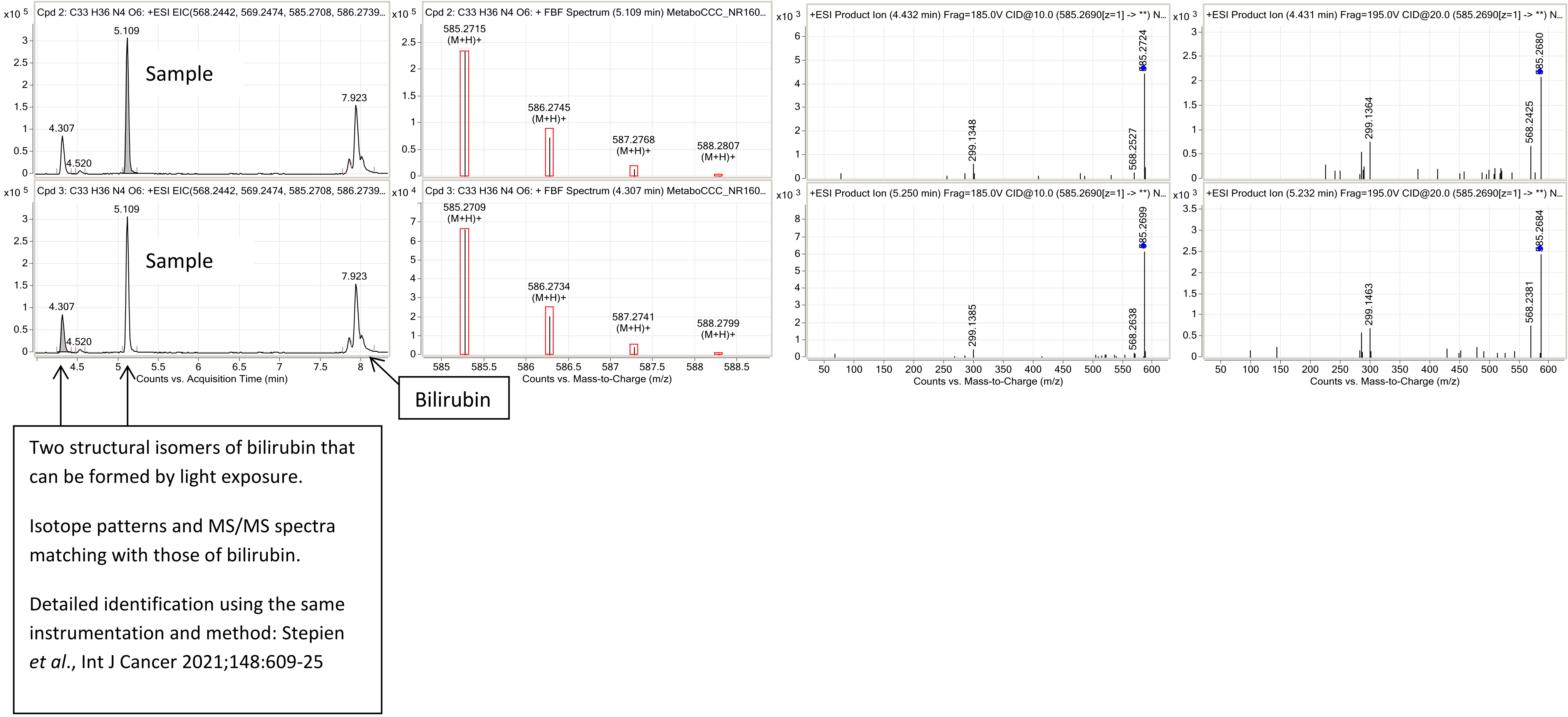

Decenoylcarnitine (C10:1), isomer 1 and 2

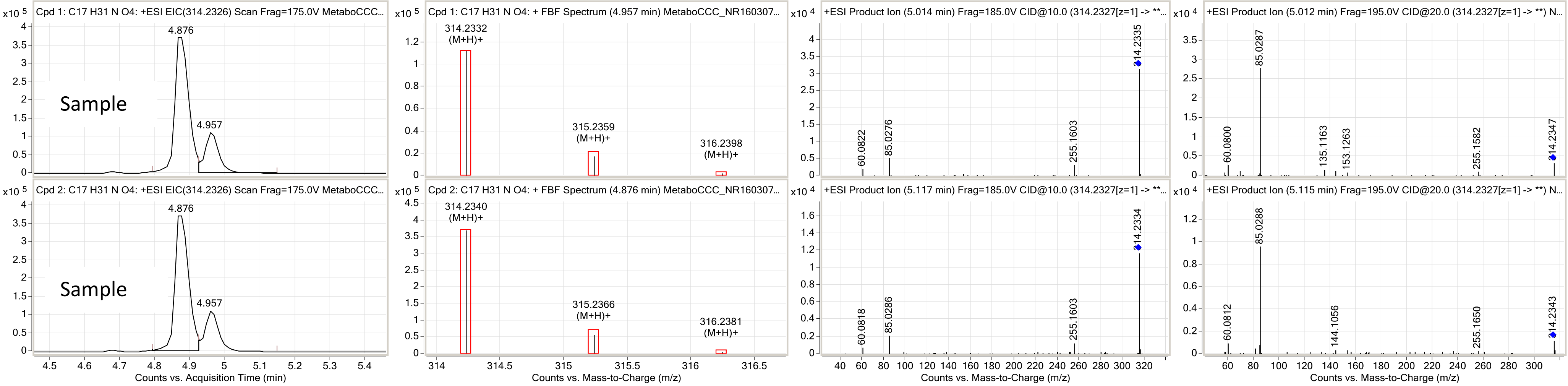

Dodecenoylcarnitine (C12:1)

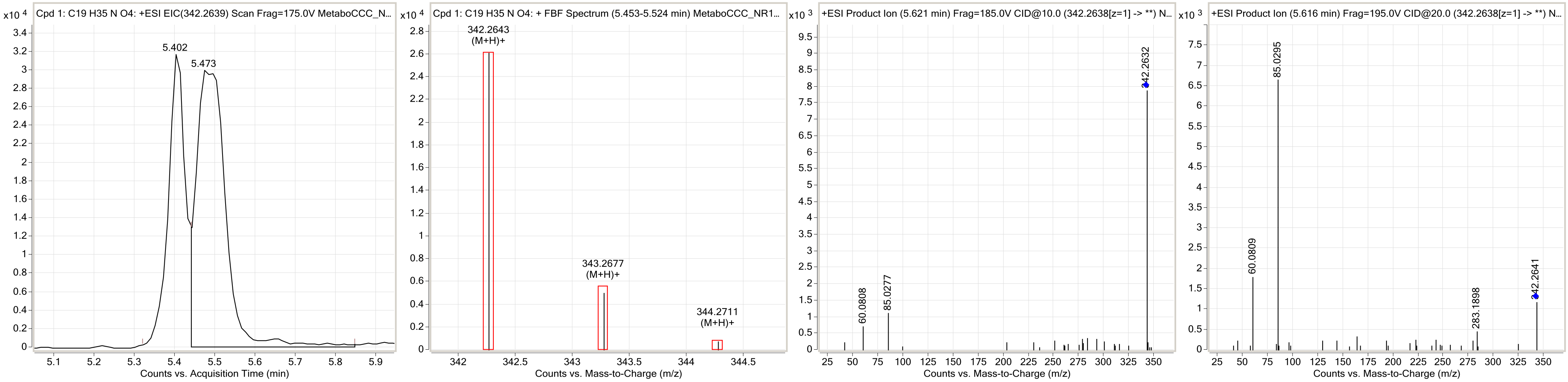

Hexadecadienoylcarnitine (C16:2)

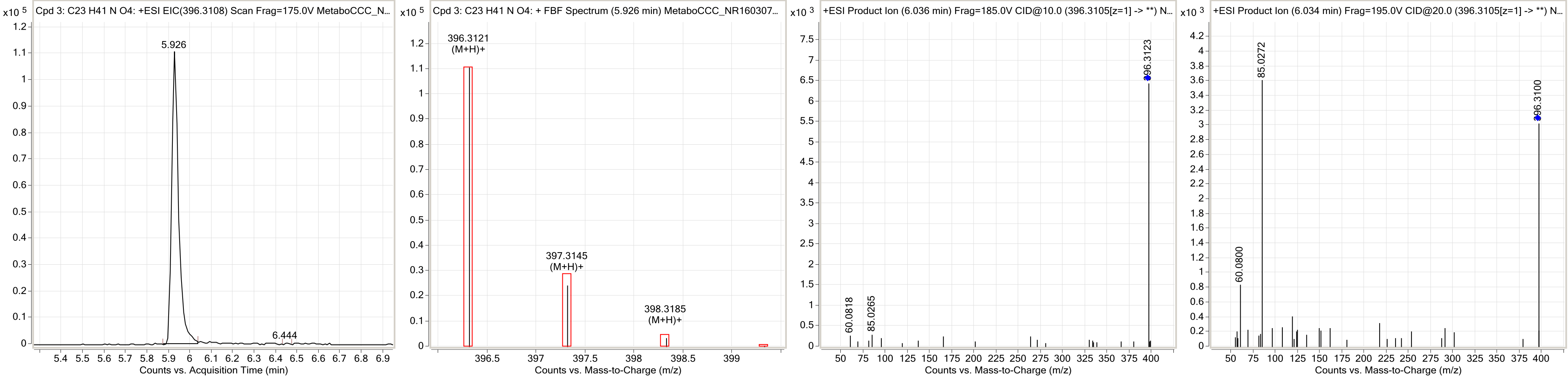

Hexadecenoylcarnitine (C16:1)

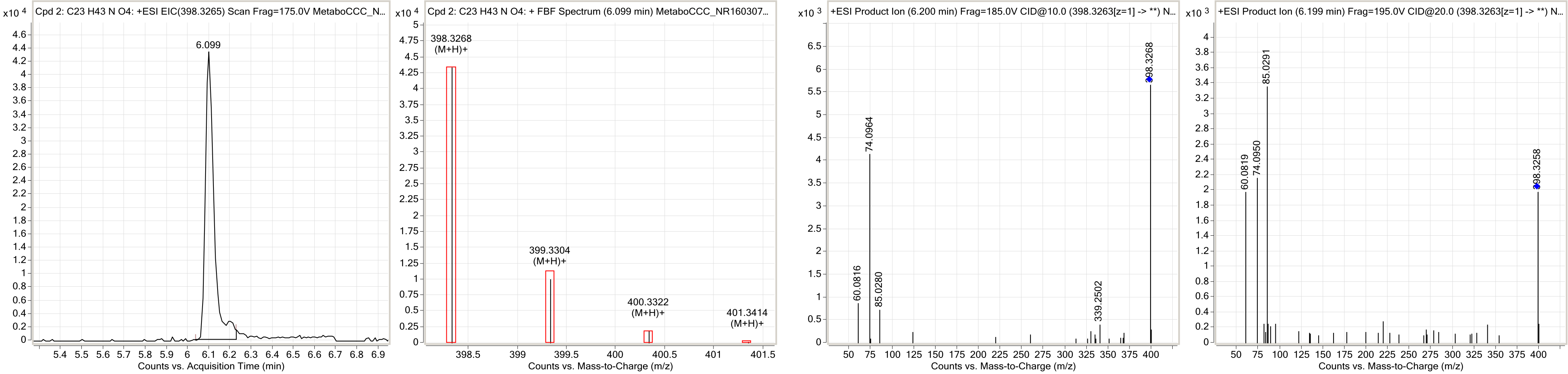

Hexanoylcarnitine (C6:0)

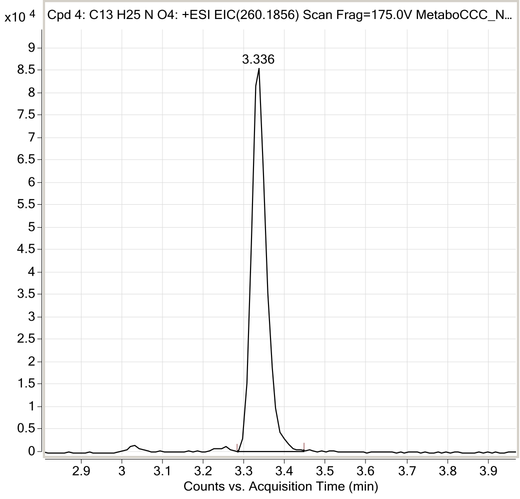

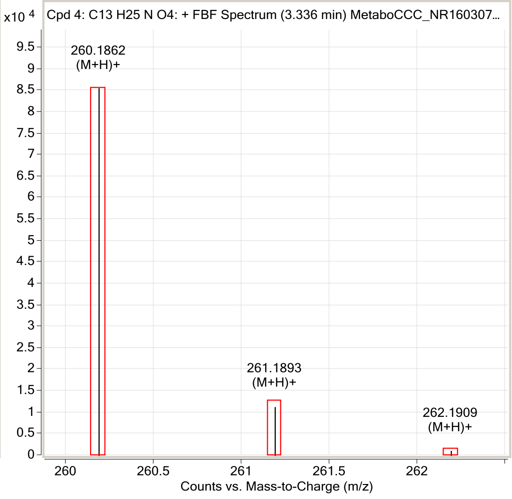

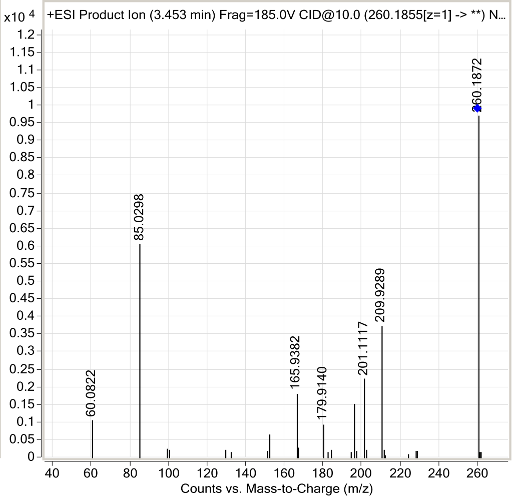

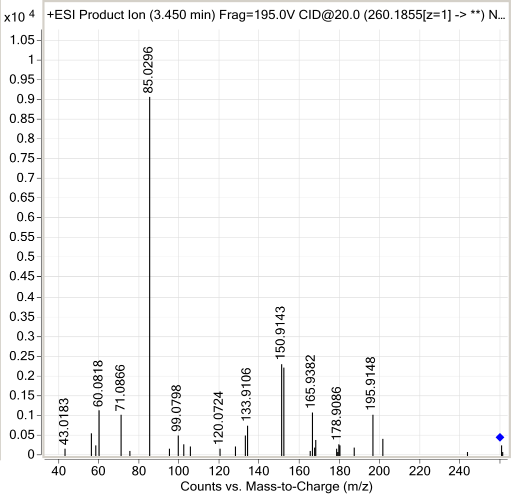

Octanoylcarnitine (C8:0)

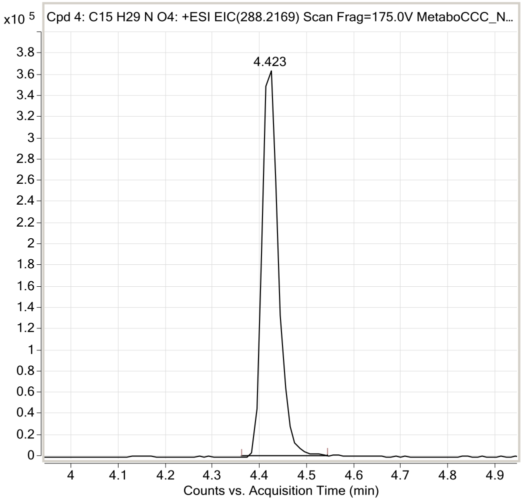

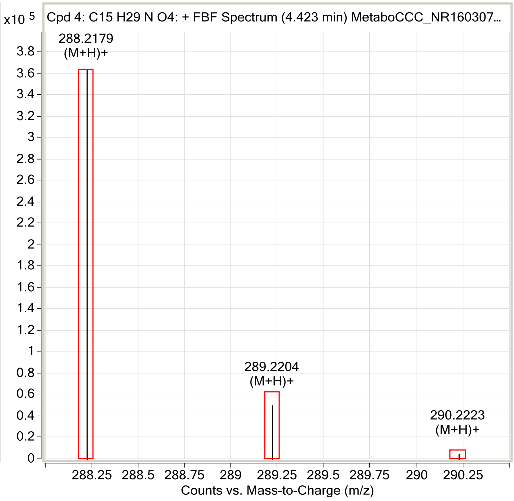

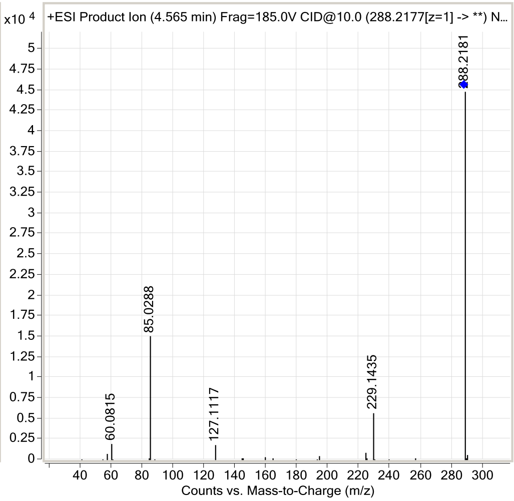

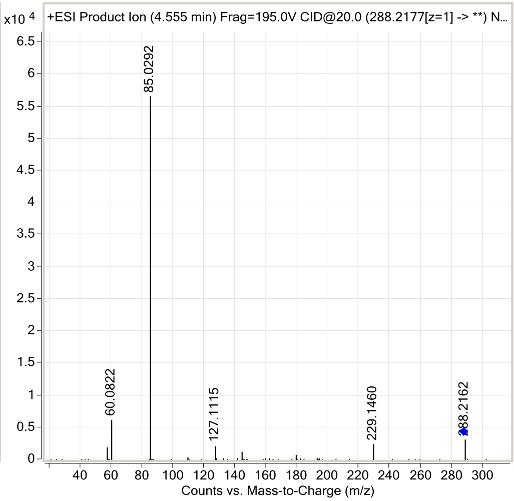

LysoPC (14:0) (isomer 1)

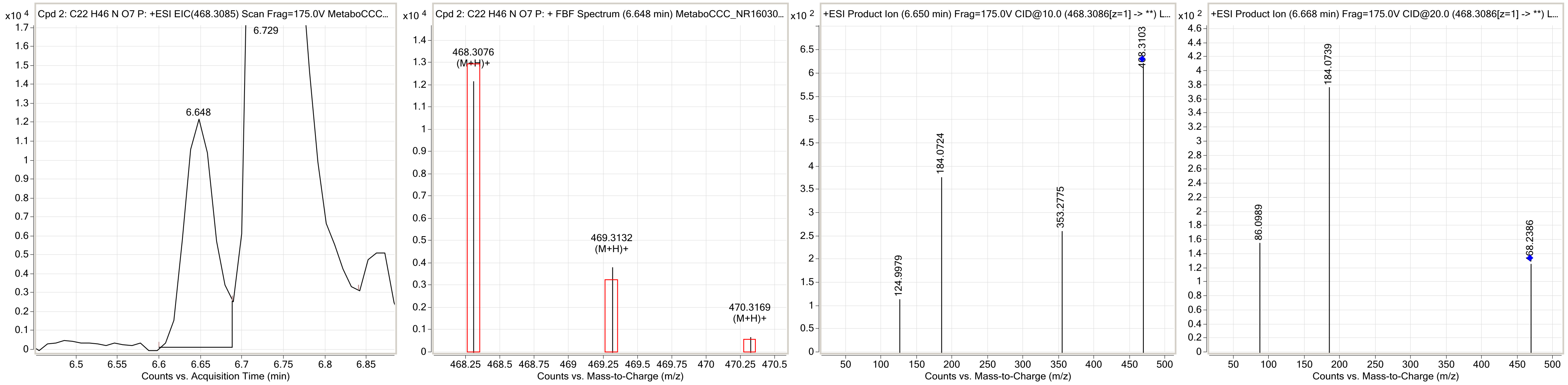

LysoPC (18:1)

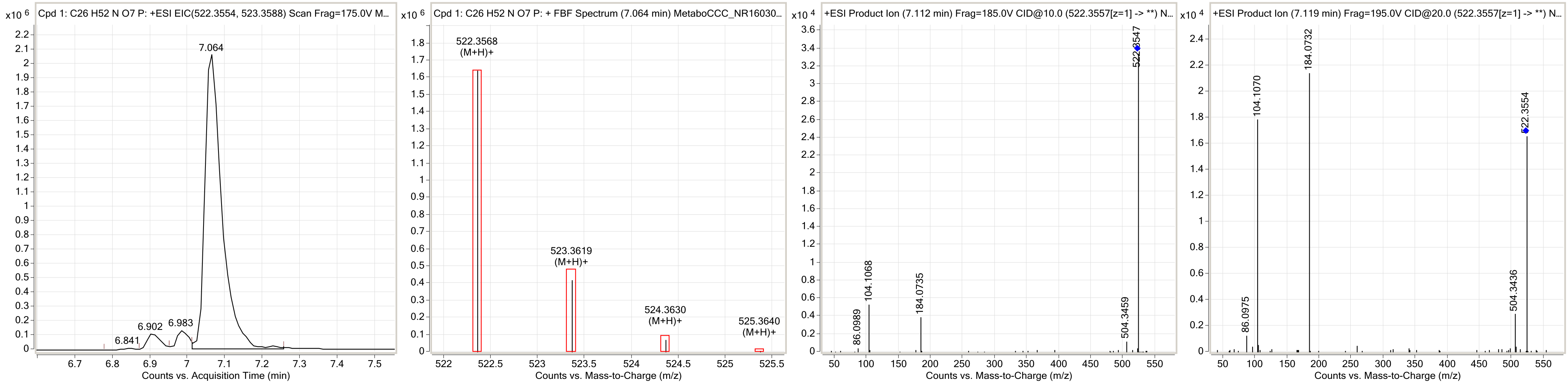

LysoPC (20:4)

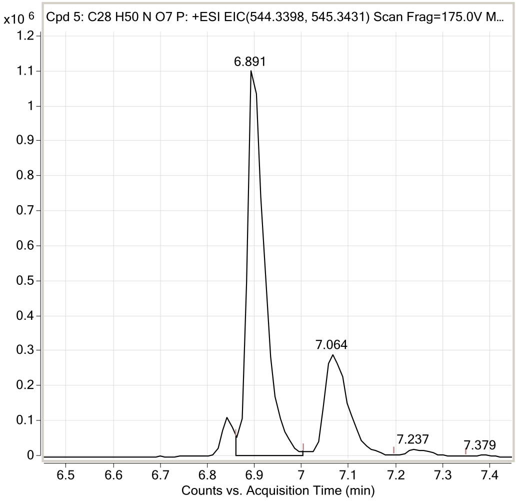

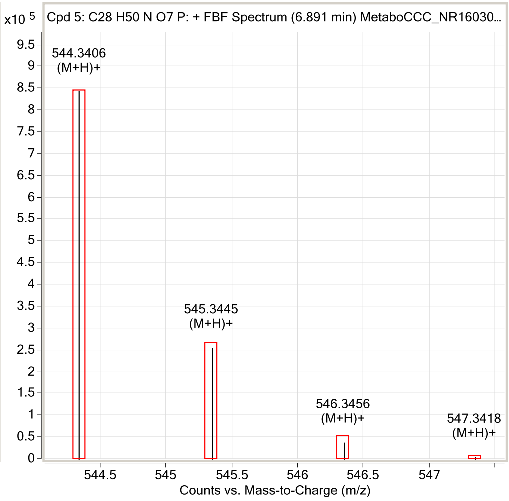

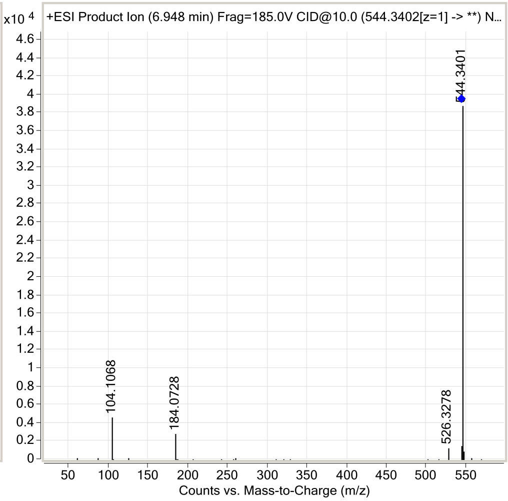

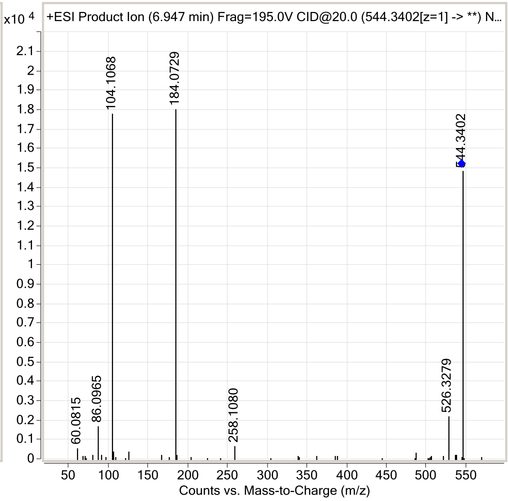

LysoPC (20:5)

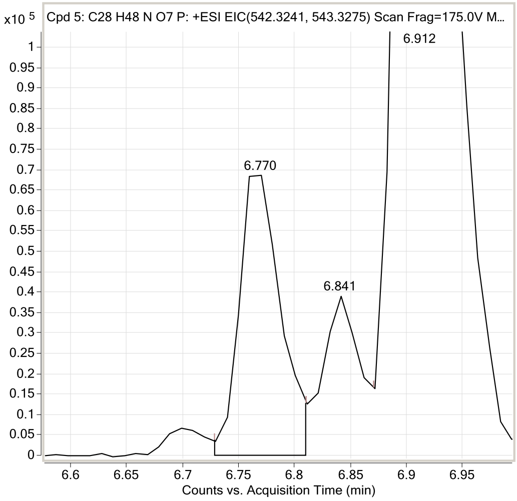

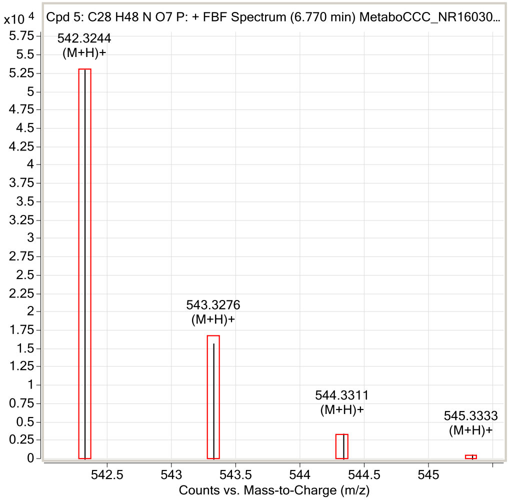

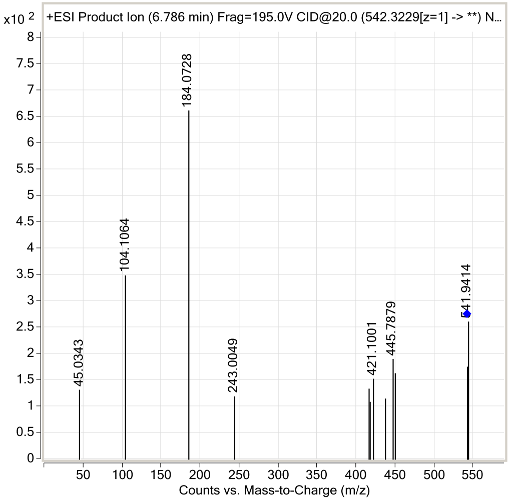

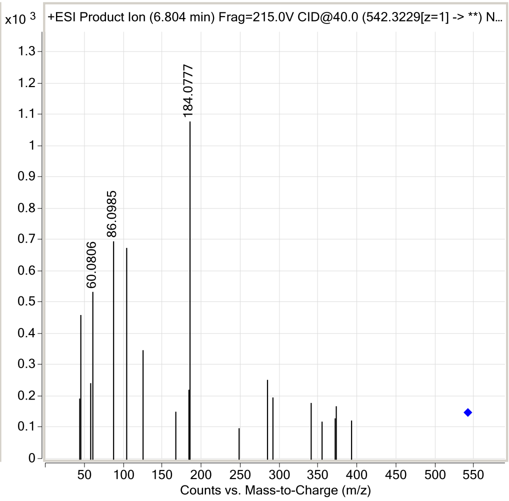

LysoPC (22:5)

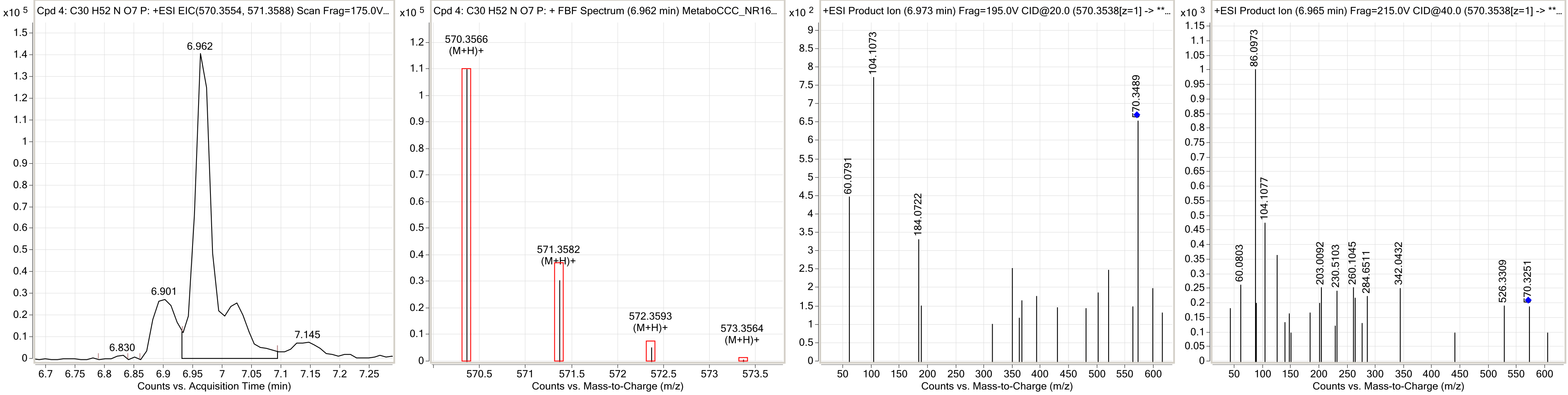

LysoPC (22:6)

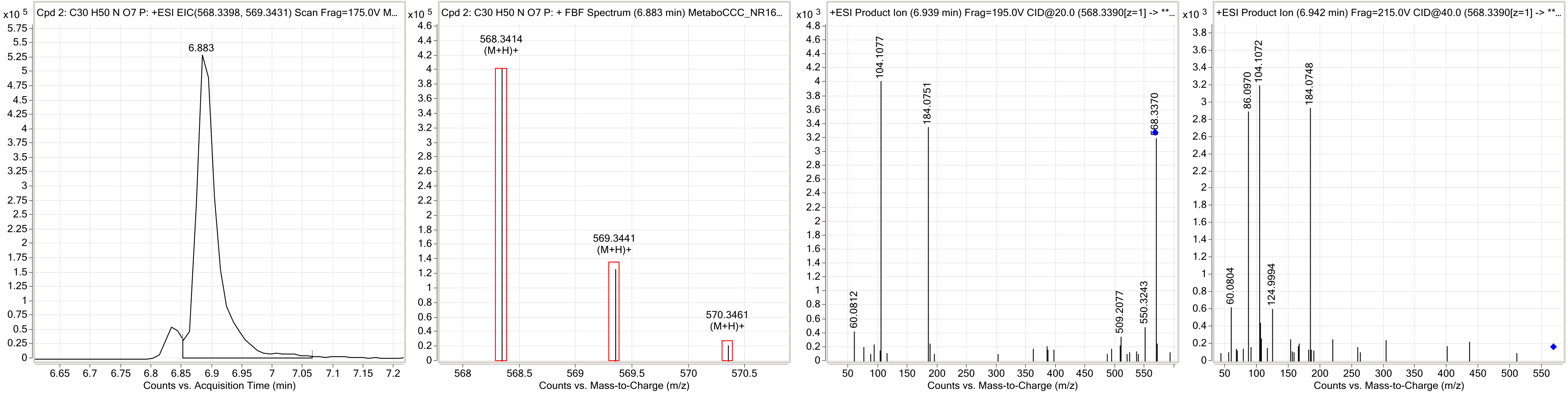

Nonanoylcarnitine (C9:0)

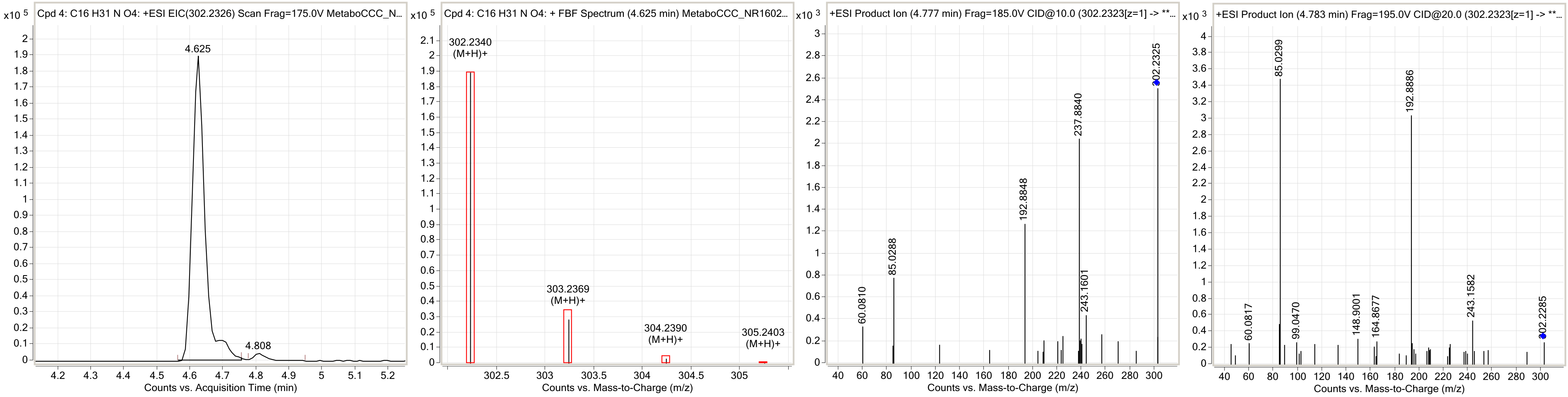

PC (36:4)

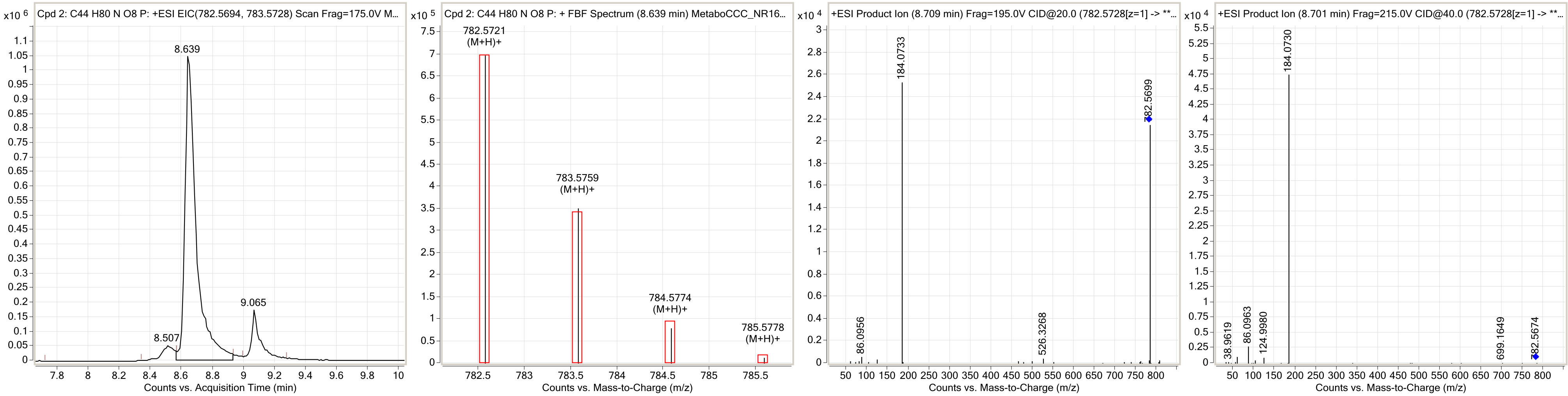

PC (38:4)

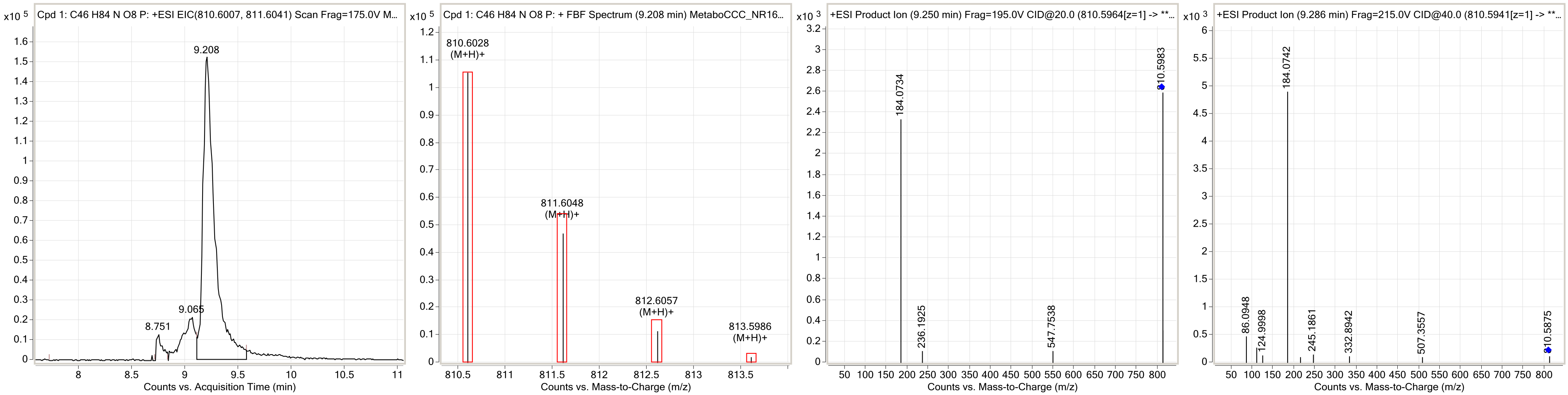

Tetradecadienoylcarnitine (C14:2)

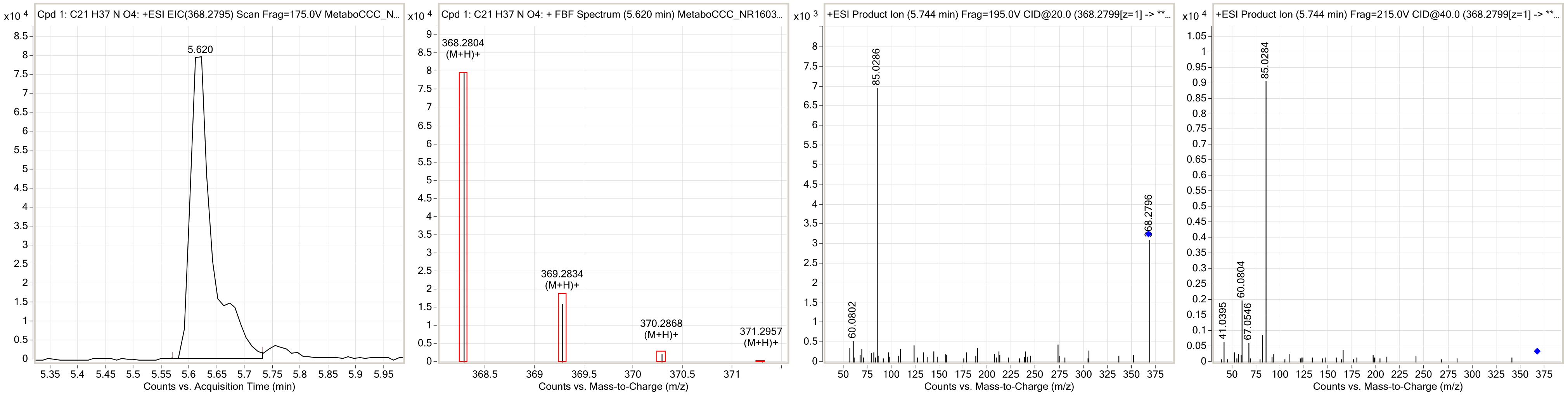

Tetradecenoylcarnitine (C14:1)

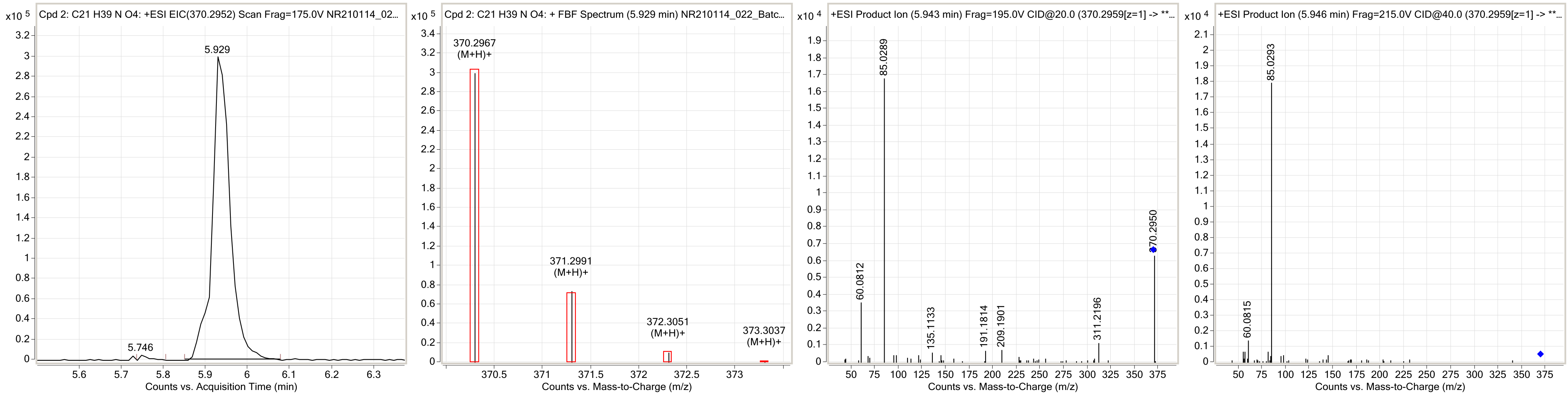

LysoPC (14:0) (isomer 2)

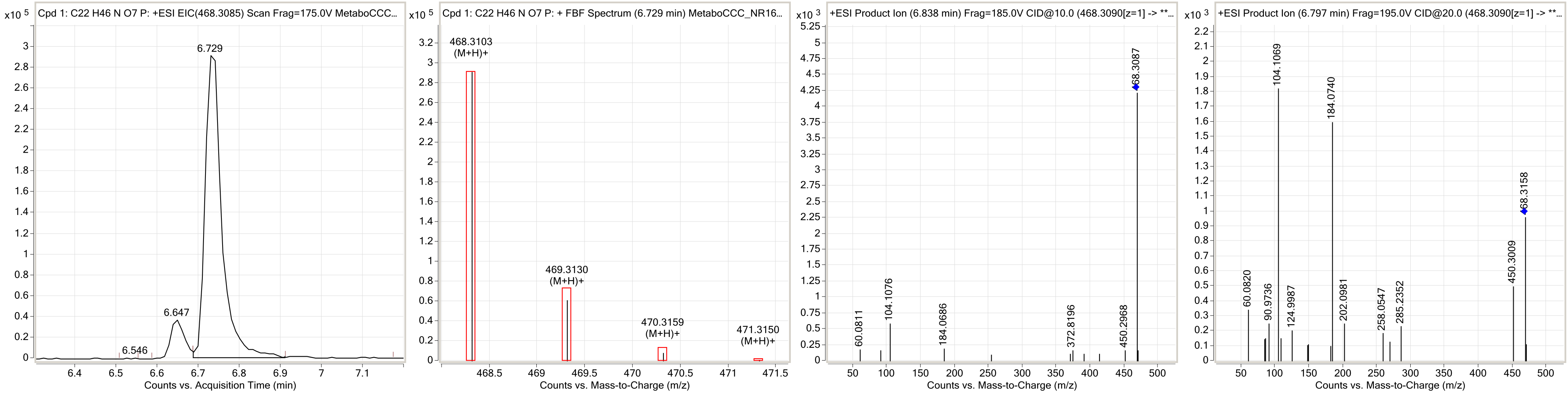

Choline

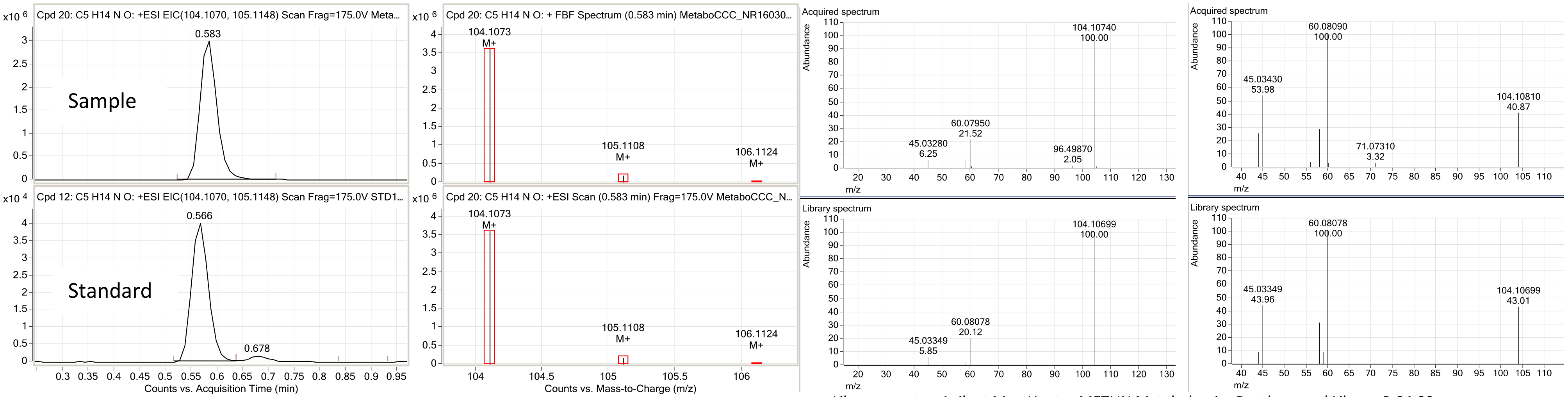

Library spectra: Agilent MassHunter METLIN Metabolomics Database and Library B.04.00

Phenylacetylglutamine

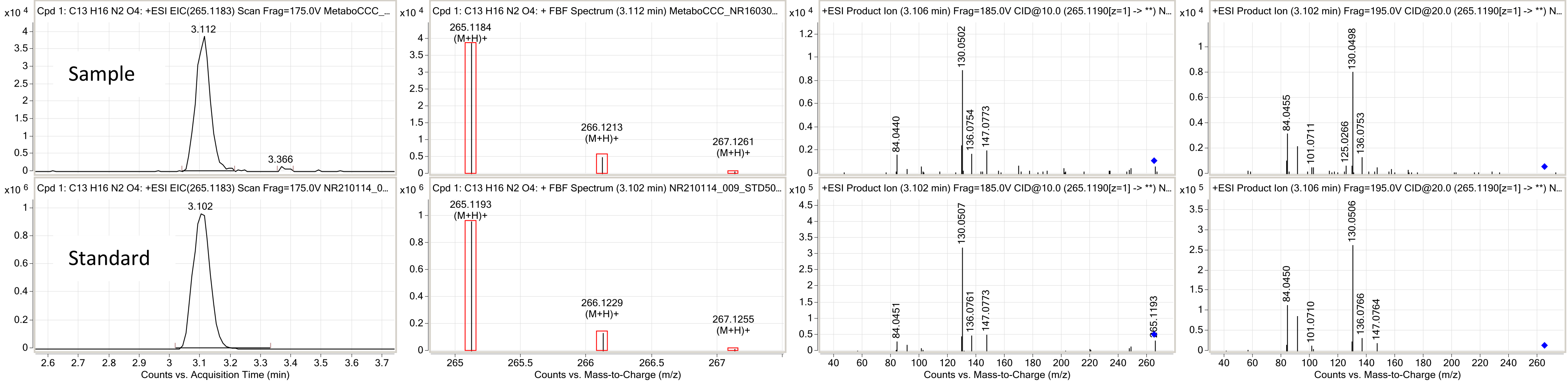

Carnitine

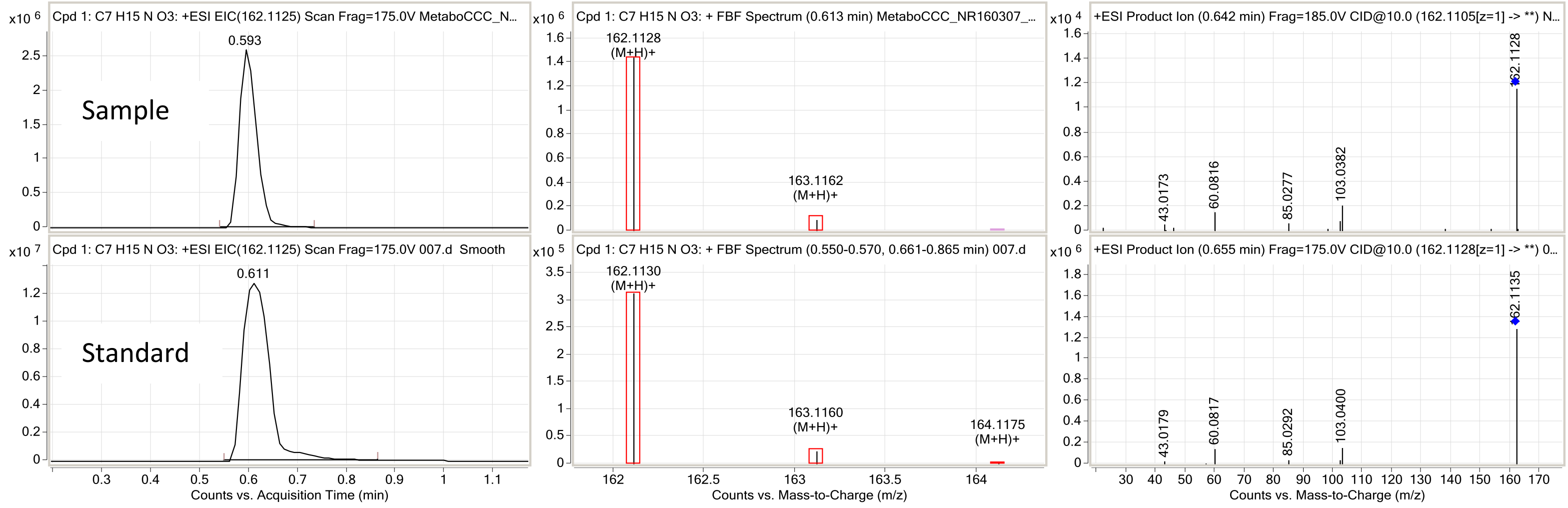

Docosahexaenoic acid

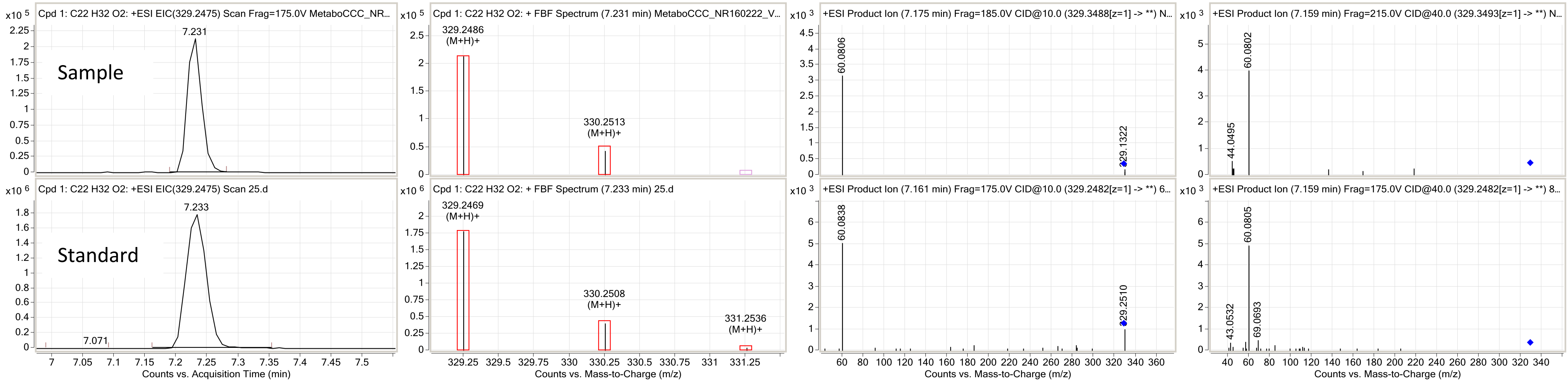

Supplement: Supplementary file 1 [file metabolites-11-00119-s001.zip › metabolites-1056311-supple-for conversion/Supplementary File S4 revised_proof.pdf]
