# Supplementary material for: Untargeted Metabolomics Reveals Major Differences in the Plasma Metabolome between Colorectal Cancer and Colorectal Adenomas
Source: Metabolites. 2021 Feb 19;11(2):119. doi: 10.3390/metabo11020119 (PMC7922413; doi:10.3390/metabo11020119)
Supplement: Supplementary file 1 [file metabolites-11-00119-s001.zip › metabolites-1056311-supple-for conversion/Supplementary Table S1 revised_proof.docx]

**Supplementary Table S1.** Complete list of 442 statistically significant features. Univariate logistic regression models analyzing three case-control comparison groups resulted in 442 significant (FDR adjusted p < 0.05) metabolic features after adjustment for age at diagnosis, sex, Body Mass Index (BMI) and smoking status. Marked in red are q-values, which did not reach statistical significance. If two or more metabolic features were assigned to the same metabolite identification, a representative feature with the highest intensity was selected (in bold).

|  | | | | **CRC vs. HR+LR** | | **CRC vs. HR** | | **CRC vs. LR** | |
| --- | --- | --- | --- | --- | --- | --- | --- | --- | --- |
| **Tentative identity** | **Metabolite name** | **RT (min) ^a^** | **m/z ^b^** | **q value ^c^** | **OR [CI.low; CI.up] ^d^** | **q value ^c^** | **OR [CI.low; CI.up] ^d^** | **q value ^c^** | **OR [CI.low; CI.up] ^d^** |
| 136.0638@0.5890086 | **1-methylnicotinamide** | 0.59 | 137.0711 | 9.46 × 10^-9^ | 0.2 [0.12; 0.34] | 3.44 × 10^-7^ | 0.2 [0.11; 0.35] | 1.00 × 10^-7^ | 0.19 [0.1; 0.34] |
| 644.2829@7.9354935 | Bilirubin | 7.94 | 645.2902 | 1.93 × 10^-5^ | 0.38 [0.25; 0.58] | 1.30 × 10^-4^ | 0.39 [0.24; 0.6] | 2.53 × 10^-4^ | 0.41 [0.26; 0.63] |
| 283.1076@7.9350643 | Bilirubin | 7.94 | 284.1149 | 2.57 × 10^-2^ | 0.49 [0.28; 0.85] | 4.23 × 10^-2^ | 0.51 [0.28; 0.88] | 5.49 × 10^-2^ | 0.5 [0.27; 0.91] |
| 612.2573@7.935085 | Bilirubin | 7.94 | 613.2646 | 1.06 × 10^-5^ | 0.32 [0.2; 0.52] | 1.42 × 10^-4^ | 0.35 [0.21; 0.57] | 1.11 × 10^-4^ | 0.34 [0.2; 0.56] |
| 580.232@7.93404 | Bilirubin | 7.93 | 581.2393 | 9.79 × 10^-5^ | 0.39 [0.25; 0.6] | 6.31 × 10^-4^ | 0.41 [0.25; 0.65] | 5.23 × 10^-4^ | 0.38 [0.23; 0.63] |
| 284.1159@7.93434 | Bilirubin | 7.93 | 285.1232 | 3.88 × 10^-4^ | 0.35 [0.2; 0.6] | 2.19 × 10^-3^ | 0.39 [0.22; 0.67] | 1.49 × 10^-3^ | 0.35 [0.19; 0.63] |
| 582.2481@7.933631 | **Bilirubin** | 7.93 | 583.2554 | 2.53 × 10^-4^ | 0.46 [0.31; 0.67] | 8.11 × 10^-4^ | 0.45 [0.29; 0.69] | 1.73 × 10^-3^ | 0.48 [0.31; 0.72] |
| 298.1318@7.934238 | Bilirubin | 7.93 | 299.1391 | 8.39 × 10^-5^ | 0.4 [0.25; 0.61] | 6.52 × 10^-4^ | 0.42 [0.26; 0.65] | 3.71 × 10^-4^ | 0.39 [0.23; 0.62] |
| 314.1272@7.9359527 | Bilirubin | 7.94 | 315.1345 | 6.87 × 10^-6^ | 0.37 [0.24; 0.55] | 1.31 × 10^-4^ | 0.4 [0.25; 0.61] | 5.46 × 10^-5^ | 0.37 [0.23; 0.58] |
| 602.2135@7.93412 | Bilirubin | 7.93 | 603.2208 | 1.10 × 10^-4^ | 0.33 [0.19; 0.55] | 4.20 × 10^-4^ | 0.32 [0.17; 0.57] | 1.32 × 10^-3^ | 0.36 [0.19; 0.63] |
| 328.1427@7.9361415 | Bilirubin | 7.94 | 329.1500 | 1.26 × 10^-3^ | 0.42 [0.25; 0.67] | 4.13 × 10^-3^ | 0.43 [0.25; 0.71] | 3.62 × 10^-3^ | 0.41 [0.24; 0.7] |
| 584.2612@4.311188 | **Bilirubin isomer (1)** | 4.31 | 585.2685 | 8.43 × 10^-3^ | 0.46 [0.27; 0.77] | 1.91 × 10^-2^ | 0.48 [0.27; 0.82] | 2.55 × 10^-2^ | 0.48 [0.26; 0.84] |
| 615.2449@5.115586 | Bilirubin isomer (2) | 5.12 | 616.2522 | 4.30 × 10^-2^ | 0.44 [0.21; 0.87] | 8.66 × 10^-2^ | 0.47 [0.22; 0.96] | 5.40 × 10^-2^ | 0.42 [0.19; 0.88] |
| 582.247@5.1140504 | Bilirubin isomer (2) | 5.11 | 583.2543 | 4.95 × 10^-8^ | 0.32 [0.21; 0.47] | 1.43 × 10^-5^ | 0.38 [0.25; 0.57] | 1.00 × 10^-7^ | 0.29 [0.18; 0.45] |
| 584.2623@5.113845 | **Bilirubin isomer (2)** | 5.11 | 585.2696 | 4.90 × 10^-7^ | 0.33 [0.21; 0.5] | 7.06 × 10^-5^ | 0.39 [0.25; 0.59] | 7.60 × 10^-7^ | 0.29 [0.17; 0.46] |
| 194.0811@3.1936665 | **Caffeine** | 3.19 | 195.0884 | 2.14 × 10^-3^ | 1.28 [1.11; 1.49] | 3.19 × 10^-2^ | 1.22 [1.05; 1.42] | 8.29 × 10^-4^ | 1.35 [1.15; 1.6] |
| 161.1059@0.59178096 | **Carnitine** | 0.59 | 162.1132 | 1.15 × 10^-2^ | 0.22 [0.08; 0.61] | 4.63 × 10^-2^ | 0.26 [0.08; 0.78] | 1.55 × 10^-2^ | 0.21 [0.07; 0.63] |
| 296.1461@0.6034809 | Carnitine | 0.60 | 297.1534 | 3.02 × 10^-3^ | 0.24 [0.1; 0.55] | 2.19 × 10^-2^ | 0.29 [0.12; 0.72] | 3.58 × 10^-3^ | 0.21 [0.08; 0.53] |
| 103.1004@0.58044595 | **Choline** | 0.58 | 104.1077 | 4.67 × 10^-2^ | 0.26 [0.09; 0.8] | 7.77 × 10^-2^ | 0.28 [0.08; 0.9] | 6.12 × 10^-2^ | 0.23 [0.06; 0.83] |
| 315.2422@5.1313124 | **Decanoylcarnitine (C10:0)** | 5.13 | 316.2495 | 7.29 × 10^-3^ | 0.53 [0.35; 0.8] | 1.89 × 10^-2^ | 0.54 [0.34; 0.84] | 1.48 × 10^-2^ | 0.53 [0.33; 0.82] |
| 313.2255@4.8713107 | **Decenoylcarnitine (C10:1) (isomer 1)** | 4.87 | 314.2328 | 1.61 × 10^-3^ | 0.44 [0.27; 0.7] | 5.43 × 10^-3^ | 0.46 [0.28; 0.75] | 4.26 × 10^-3^ | 0.43 [0.25; 0.72] |
| 313.2254@4.958586 | **Decenoylcarnitine (C10:1) (isomer 2)** | 4.96 | 314.2327 | 7.58 × 10^-4^ | 0.35 [0.2; 0.61] | 1.23 × 10^-3^ | 0.32 [0.17; 0.59] | 4.38 × 10^-3^ | 0.37 [0.2; 0.68] |
| 328.2402@7.2288437 | **Docosahe×aenoic acid** | 7.23 | 329.2475 | 7.37 × 10^-3^ | 0.45 [0.27; 0.75] | 9.72 × 10^-3^ | 0.44 [0.25; 0.75] | 4.20 × 10^-2^ | 0.49 [0.27; 0.88] |
| 343.2731@5.6354885 | **Dodecanoylcarnitine (C12:0)** | 5.64 | 344.2804 | 9.34 × 10^-5^ | 0.37 [0.23; 0.58] | 4.88 × 10^-4^ | 0.37 [0.22; 0.61] | 6.72 × 10^-4^ | 0.37 [0.22; 0.62] |
| 341.2565@5.4976096 | **Dodecenoylcarnitine (C12:1)** | 5.50 | 342.2638 | 4.40 × 10^-4^ | 0.33 [0.19; 0.58] | 1.50 × 10^-3^ | 0.32 [0.17; 0.6] | 1.39 × 10^-3^ | 0.33 [0.17; 0.61] |
| 431.3033@6.4378824 | Glycochenodeo×ycholic acid | 6.44 | 432.3106 | 2.79 × 10^-2^ | 1.45 [1.09; 1.91] | 3.58 × 10^-2^ | 1.48 [1.09; 2.02] | 4.40 × 10^-2^ | 1.49 [1.07; 2.09] |
| 449.3143@6.4378443 | **Glycochenodeo×ycholic acid** | 6.44 | 450.3216 | 1.37 × 10^-2^ | 1.46 [1.13; 1.9] | 3.58 × 10^-2^ | 1.45 [1.08; 1.95] | 1.93 × 10^-2^ | 1.52 [1.12; 2.08] |
| 395.3032@5.9289203 | **He×adecadienoylcarnitine (C16:2)** | 5.93 | 396.3105 | 7.41 × 10^-5^ | 0.29 [0.16; 0.51] | 1.12 × 10^-4^ | 0.27 [0.14; 0.49] | 2.27 × 10^-3^ | 0.33 [0.17; 0.63] |
| 397.319@6.09628 | **He×adecenoylcarnitine (C16:1)** | 6.10 | 398.3263 | 9.27 × 10^-7^ | 0.22 [0.12; 0.39] | 5.98 × 10^-6^ | 0.21 [0.11; 0.39] | 6.27 × 10^-5^ | 0.25 [0.13; 0.47] |
| 259.1782@3.3313515 | **He×anoylcarnitine (C6:0)** | 3.33 | 260.1855 | 1.86 × 10^-3^ | 0.42 [0.25; 0.68] | 2.66 × 10^-3^ | 0.39 [0.22; 0.68] | 1.16 × 10^-2^ | 0.45 [0.25; 0.77] |
| 179.0584@3.0740817 | **Hippuric acid** | 3.07 | 180.0657 | 6.52 × 10^-21^ | 3.15 [2.46; 4.13] | 1.04 × 10^-16^ | 3.31 [2.46; 4.61] | 2.03 × 10^-18^ | 3.66 [2.66; 5.24] |
| 104.027@3.0739033 | Hippuric acid | 3.07 | 105.0343 | 2.46 × 10^-19^ | 3.43 [2.6; 4.65] | 1.39 × 10^-15^ | 3.69 [2.62; 5.4] | 3.66 × 10^-17^ | 4.17 [2.89; 6.31] |
| 136.0384@0.85652673 | Hypo×anthine | 0.86 | 137.0457 | 8.42 × 10^-3^ | 2.11 [1.29; 3.57] | 1.23 × 10^-3^ | 2.66 [1.54; 4.87] | 1.20 × 10^-1^ | 1.72 [0.99; 3.06] |
| 136.0383@1.1590997 | **Hypo×anthine** | 1.16 | 137.0456 | 7.60 × 10^-3^ | 2.14 [1.3; 3.59] | 8.79 × 10^-4^ | 2.79 [1.61; 5.11] | 1.17 × 10^-1^ | 1.7 [0.99; 2.97] |
| 189.0797@4.563414 | **Indole-3-propionic acid** | 4.56 | 190.0870 | 1.19 × 10^-12^ | 2.57 [1.99; 3.37] | 9.34 × 10^-12^ | 2.79 [2.08; 3.86] | 9.52 × 10^-10^ | 2.6 [1.93; 3.6] |
| 174.056@4.1268005 | Indoleacetic acid | 4.13 | 175.0633 | 2.19 × 10^-10^ | 4.61 [2.93; 7.52] | 3.44 × 10^-7^ | 3.95 [2.42; 6.76] | 7.61 × 10^-11^ | 6.68 [3.72; 12.8] |
| 197.0453@4.1268587 | Indoleacetic acid | 4.13 | 198.0526 | 2.32 × 10^-10^ | 4.43 [2.84; 7.15] | 5.68 × 10^-7^ | 3.75 [2.32; 6.36] | 4.11 × 10^-11^ | 6.84 [3.81; 13.13] |
| 129.0574@4.127236 | Indoleacetic acid | 4.13 | 130.0647 | 4.68 × 10^-10^ | 4.83 [3; 8.08] | 6.93 × 10^-7^ | 4.07 [2.43; 7.15] | 1.03 × 10^-10^ | 7.31 [3.94; 14.56] |
| 175.0643@4.126671 | **Indoleacetic acid** | 4.13 | 176.0716 | 1.17 × 10^-10^ | 4.23 [2.77; 6.68] | 3.03 × 10^-7^ | 3.66 [2.31; 6.06] | 3.27 × 10^-11^ | 6.19 [3.57; 11.45] |
| 259.016@4.126407 | Indoleacetic acid | 4.13 | 260.0233 | 4.60 × 10^-8^ | 4.89 [2.85; 8.8] | 3.17 × 10^-5^ | 3.93 [2.17; 7.6] | 4.17 × 10^-9^ | 9.24 [4.3; 21.98] |
| 205.075@3.8313742 | **Indolelactic acid** | 3.83 | 206.0823 | 2.70 × 10^-3^ | 3.06 [1.59; 5.98] | 2.89 × 10^-2^ | 2.49 [1.25; 5.1] | 2.00 × 10^-3^ | 3.66 [1.75; 7.99] |
| 129.0578@3.8321202 | Indolelactic acid | 3.83 | 130.0651 | 2.87 × 10^-2^ | 2.38 [1.22; 4.72] | 9.79 × 10^-2^ | 2.06 [1.02; 4.26] | 2.93 × 10^-2^ | 2.62 [1.25; 5.68] |
| 147.0321@3.310721 | **Isatin** | 3.31 | 148.0394 | 7.34 × 10^-12^ | 5.01 [3.2; 8.09] | 1.84 × 10^-9^ | 4.99 [3.02; 8.66] | 5.49 × 10^-10^ | 5.17 [3.11; 8.98] |
| 541.3156@6.7672586 | **Lyso PC (20:5)** | 6.77 | 542.3229 | 1.47 × 10^-1^ | 0.7 [0.47; 1.04] | 6.21 × 10^-1^ | 0.86 [0.57; 1.32] | 2.05 × 10^-2^ | 0.55 [0.35; 0.85] |
| 467.3017@6.645774 | **LysoPC (14:0) (isomer 1)** | 6.65 | 468.3090 | 1.98 × 10^-1^ | 0.63 [0.35; 1.12] | 7.06 × 10^-1^ | 0.85 [0.46; 1.56] | 1.61 × 10^-2^ | 0.39 [0.2; 0.76] |
| 489.2833@6.7325006 | LysoPC (14:0) (isomer 2) | 6.73 | 490.2906 | 1.43 × 10^-1^ | 0.59 [0.33; 1.05] | 4.45 × 10^-1^ | 0.73 [0.4; 1.34] | 2.82 × 10^-2^ | 0.43 [0.22; 0.82] |
| 467.3003@6.731867 | **LysoPC (14:0) (isomer 2)** | 6.73 | 468.3076 | 3.02 × 10^-2^ | 0.55 [0.34; 0.88] | 1.98 × 10^-1^ | 0.67 [0.41; 1.09] | 4.95 × 10^-3^ | 0.43 [0.25; 0.73] |
| 481.3157@6.879825 | **LysoPC (15:0)** | 6.88 | 482.3230 | 1.98 × 10^-2^ | 0.47 [0.27; 0.82] | 2.68 × 10^-1^ | 0.65 [0.36; 1.18] | 1.84 × 10^-3^ | 0.33 [0.17; 0.62] |
| 503.2963@6.8837066 | LysoPC (15:0) | 6.88 | 504.3036 | 1.08 × 10^-3^ | 0.19 [0.08; 0.46] | 2.40 × 10^-2^ | 0.27 [0.1; 0.71] | 2.49 × 10^-4^ | 0.12 [0.04; 0.34] |
| 495.3327@6.995685 | **LysoPC (16:0)** | 7.00 | 496.3400 | 1.07 × 10^-7^ | 0.04 [0.01; 0.12] | 2.69 × 10^-5^ | 0.06 [0.02; 0.2] | 4.02 × 10^-8^ | 1.75 × 10^-2^ [3.84 × 10^-3^; 7.04 × 10^-2^] |
| 761.9746@6.997951 | LysoPC (16:0) | 7.00 | 762.9819 | 1.37 × 10^-10^ | 0.02 [0.01; 0.07] | 5.46 × 10^-7^ | 0.04 [0.01; 0.13] | 7.33 × 10^-11^ | 1.01 × 10^-2^ [2.16 × 10^-3^; 4.10 × 10^-2^] |
| 762.4779@6.998174 | LysoPC (16:0) | 7.00 | 763.4852 | 5.92 × 10^-11^ | 0.02 [0.01; 0.07] | 3.44 × 10^-7^ | 0.04 [0.01; 0.13] | 4.31 × 10^-11^ | 9.75 × 10^-3^ [2.08 × 10^-3^; 3.93 × 10^-2^] |
| 506.3187@6.996779 | LysoPC (16:0) | 7.00 | 507.3260 | 1.21 × 10^-7^ | 0.03 [0.01; 0.1] | 4.63 × 10^-5^ | 0.06 [0.02; 0.19] | 4.16 × 10^-8^ | 1.54 × 10^-2^ [3.34 × 10^-3^; 6.35 × 10^-2^] |
| 521.7916@6.9972916 | LysoPC (16:0) | 7.00 | 522.7989 | 3.65 × 10^-3^ | 0.26 [0.11; 0.58] | 3.34 × 10^-2^ | 0.33 [0.13; 0.78] | 1.78 × 10^-3^ | 0.2 [0.07; 0.49] |
| 753.9826@6.998699 | LysoPC (16:0) | 7.00 | 754.9899 | 1.25 × 10^-9^ | 1.56 × 10^-9^ [4.00 × 10^-3^; 5.53 × 10^-2^] | 2.49 × 10^-6^ | 0.03 [0.01; 0.11] | 5.49 × 10^-10^ | 5.48 × 10^-3^ [9.00 × 10^-4^; 2.82 × 10^-2^] |
| 771.4615@6.9971037 | LysoPC (16:0) | 7.00 | 772.4688 | 3.10 × 10^-8^ | 0.05 [0.02; 0.13] | 1.01 × 10^-5^ | 0.07 [0.02; 0.21] | 2.03 × 10^-8^ | 0.03 [0.01; 0.09] |
| 770.9597@6.9973145 | LysoPC (16:0) | 7.00 | 771.9670 | 1.21 × 10^-7^ | 0.07 [0.03; 0.17] | 3.31 × 10^-5^ | 0.1 [0.04; 0.27] | 6.38 × 10^-8^ | 0.04 [0.01; 0.12] |
| 754.4815@6.9985743 | LysoPC (16:0) | 7.00 | 755.4888 | 8.16 × 10^-10^ | 1.59 × 10^-2^ [4.22 × 10^-3^; 5.54 × 10^-2^] | 1.74 × 10^-6^ | 0.03 [0.01; 0.11] | 5.49 × 10^-10^ | 5.98 × 10^-3^ [9.91 × 10^-4^; 3.02 × 10^-2^] |
| 517.3147@6.996175 | LysoPC (16:0) | 7.00 | 518.3220 | 1.88 × 10^-4^ | 0.08 [0.03; 0.27] | 4.79 × 10^-3^ | 0.13 [0.04; 0.45] | 3.63 × 10^-5^ | 0.04 [0.01; 0.15] |
| 990.6655@6.9949603 | LysoPC (16:0) | 6.99 | 991.6728 | 6.62 × 10^-9^ | 0.2 [0.12; 0.34] | 4.00 × 10^-6^ | 0.25 [0.14; 0.42] | 4.96 × 10^-9^ | 0.15 [0.08; 0.28] |
| [506.8181@6.9990873](mailto:506.8181@6.9990873) | LysoPC (16:0) | 7.00 | 507.8254 | 1.16 × 10^-12^ | 1.63 × 10^-3^ [2.59 × 10^-4^; 9.11 × 10^-3^] | 7.15 × 10^-9^ | 3.76 × 10^-3^ [5.52 × 10^-4^; 2.22 × 10^-2^] | 5.52 × 10^-12^ | 5.10 × 10^-4^ [4.35 × 10^-5^; 4.60 × 10^-3^] |
| 514.3091@6.999144 | LysoPC (16:0) | 7.00 | 515.3164 | 1.22 × 10^-11^ | 2.26 × 10^-3^ [3.66 × 10^-4^; 1.24 × 10^-2^] | 8.43 × 10^-8^ | 5.34 × 10^-3^ [8.05 × 10^-4^; 3.10 × 10^-2^] | 2.70 × 10^-11^ | 7.74 × 10^-4^ [7.23 × 10^-5^; 6.52 × 10^-3^] |
| 514.811@6.998849 | LysoPC (16:0) | 7.00 | 515.8183 | 1.70 × 10^-11^ | 2.46 × 10^-3^ [4.05 × 10^-3^; 1.33 × 10^-2^] | 1.14 × 10^-7^ | 5.76 × 10^-3^ [8.72 × 10^-4^; 3.32 × 10^-2^] | 3.20 × 10^-11^ | 8.22 × 10^-4^ [7.81 × 10^-5^; 6.85 × 10^-3^] |
| 770.4601@6.9978433 | LysoPC (16:0) | 7.00 | 771.4674 | 2.06 × 10^-5^ | 0.14 [0.06; 0.32] | 8.06 × 10^-4^ | 0.19 [0.07; 0.45] | 1.45 × 10^-5^ | 0.1 [0.04; 0.27] |
| 769.9578@6.997808 | LysoPC (16:0) | 7.00 | 770.9651 | 3.30 × 10^-5^ | 0.16 [0.07; 0.35] | 1.20 × 10^-3^ | 0.21 [0.09; 0.48] | 2.18 × 10^-5^ | 0.12 [0.04; 0.29] |
| 523.7971@6.996559 | LysoPC (16:0) | 7.00 | 524.8044 | 3.76 × 10^-9^ | 1.29 × 10^-2^ [3.03 × 10^-3^; 5.07 × 10^-2^] | 2.82 × 10^-6^ | 2.22 × 10^-2^ [4.73 × 10^-3^; 9.41 × 10^-2^] | 4.02 × 10^-9^ | 6.10 × 10^-3^ [9.72 × 10^-4^; 3.25 × 10^-2^] |
| 523.295@6.9967065 | LysoPC (16:0) | 7.00 | 524.3023 | 2.04 × 10^-8^ | 1.77 × 10^-2^ [4.36 × 10^-3^; 6.63 × 10^-2^] | 6.30 × 10^-6^ | 0.03 [0.01; 0.12] | 2.44 × 10^-8^ | 9.50 × 10^-3^ [1.65 × 10^-3^; 4.67 × 10^-2^] |
| 522.2955@6.9974113 | LysoPC (16:0) | 7.00 | 523.3028 | 6.68 × 10^-5^ | 0.1 [0.03; 0.28] | 1.26 × 10^-3^ | 0.13 [0.04; 0.39] | 1.15 × 10^-4^ | 0.08 [0.02; 0.27] |
| 522.7951@6.997073 | LysoPC (16:0) | 7.00 | 523.8024 | 2.56 × 10^-5^ | 0.08 [0.03; 0.24] | 7.32 × 10^-4^ | 0.11 [0.04; 0.35] | 3.30 × 10^-5^ | 0.06 [0.02; 0.21] |
| 769.4553@6.9980416 | LysoPC (16:0) | 7.00 | 770.4626 | 2.51 × 10^-4^ | 0.21 [0.1; 0.45] | 3.43 × 10^-3^ | 0.25 [0.11; 0.56] | 2.63 × 10^-4^ | 0.17 [0.07; 0.4] |
| 259.1522@6.9989886 | LysoPC (16:0) | 7.00 | 260.1595 | 1.15 × 10^-2^ | 0.26 [0.1; 0.64] | 4.38 × 10^-2^ | 0.27 [0.09; 0.78] | 4.77 × 10^-3^ | 0.16 [0.05; 0.49] |
| 765.4824@7.002013 | LysoPC (16:0) | 7.00 | 766.4897 | 1.02 × 10^-5^ | 0.04 [0.01; 0.14] | 5.72 × 10^-4^ | 0.06 [0.01; 0.25] | 5.15 × 10^-6^ | 1.97 × 10^-2^ [3.69 × 10^-3^; 9.49 × 10^-2^] |
| 764.9815@7.003498 | LysoPC (16:0) | 7.00 | 765.9888 | 3.06 × 10^-6^ | 0.03 [0.01; 0.13] | 1.63 × 10^-4^ | 0.05 [0.01; 0.21] | 3.64 × 10^-6^ | 2.10 × 10^-2^ [4.11 × 10^-3^; 9.69 × 10^-2^] |
| 275.1662@6.999173 | LysoPC (16:0) | 7.00 | 276.1735 | 6.92 × 10^-2^ | 0.4 [0.18; 0.91] | 1.56 × 10^-1^ | 0.43 [0.16; 1.11] | 3.16 × 10^-2^ | 0.26 [0.09; 0.74] |
| 515.297@6.826061 | LysoPC (16:1) | 6.83 | 516.3043 | 8.67 × 10^-5^ | 0.34 [0.2; 0.56] | 2.19 × 10^-3^ | 0.4 [0.23; 0.67] | 2.28 × 10^-5^ | 0.28 [0.15; 0.48] |
| 493.317@6.8211875 | **LysoPC (16:1)** | 6.82 | 494.3243 | 3.06 × 10^-5^ | 0.32 [0.19; 0.52] | 6.90 × 10^-4^ | 0.35 [0.2; 0.6] | 1.47 × 10^-5^ | 0.27 [0.15; 0.47] |
| 266.1349@6.820541 | LysoPC (16:1) | 6.82 | 267.1422 | 3.15 × 10^-4^ | 0.3 [0.17; 0.55] | 2.67 × 10^-3^ | 0.32 [0.16; 0.62] | 2.12 × 10^-4^ | 0.26 [0.13; 0.5] |
| 258.1452@6.8205676 | LysoPC (16:1) | 6.82 | 259.1525 | 2.15 × 10^-3^ | 0.23 [0.09; 0.53] | 1.28 × 10^-2^ | 0.26 [0.1; 0.65] | 9.38 × 10^-4^ | 0.18 [0.07; 0.45] |
| 509.3466@7.126865 | **LysoPC (17:0)** | 7.13 | 510.3539 | 2.64 × 10^-3^ | 0.4 [0.23; 0.68] | 7.69 × 10^-2^ | 0.53 [0.29; 0.95] | 2.78 × 10^-4^ | 0.28 [0.14; 0.52] |
| 804.0185@7.2454095 | LysoPC (18:0) | 7.25 | 805.0258 | 1.40 × 10^-2^ | 0.41 [0.22; 0.77] | 1.22 × 10^-2^ | 0.36 [0.18; 0.72] | 5.04 × 10^-2^ | 0.47 [0.24; 0.89] |
| 551.3246@7.244477 | LysoPC (18:0) | 7.24 | 552.3319 | 1.38 × 10^-6^ | 0.25 [0.14; 0.42] | 1.34 × 10^-4^ | 0.29 [0.16; 0.51] | 1.11 × 10^-6^ | 0.2 [0.1; 0.37] |
| [523.364@7.2449145](mailto:523.364@7.2449145) | **LysoPC (18:0)** | 7.24 | 524.3713 | 2.89 × 10^-7^ | 0.15 [0.07; 0.29] | 4.55 × 10^-5^ | 0.19 [0.09; 0.39] | 3.29 × 10^-7^ | 0.11 [0.05; 0.25] |
| 534.3471@7.245101 | LysoPC (18:0) | 7.25 | 535.3544 | 3.40 × 10^-7^ | 0.2 [0.11; 0.36] | 5.47 × 10^-5^ | 0.25 [0.13; 0.46] | 2.40 × 10^-7^ | 0.15 [0.07; 0.29] |
| 550.3256@7.2449584 | LysoPC (18:0) | 7.24 | 551.3329 | 5.97 × 10^-6^ | 0.31 [0.19; 0.49] | 3.86 × 10^-4^ | 0.36 [0.21; 0.59] | 4.97 × 10^-6^ | 0.26 [0.15; 0.45] |
| 542.3386@7.2460995 | LysoPC (18:0) | 7.25 | 543.3459 | 4.83 × 10^-8^ | 0.18 [0.1; 0.32] | 1.30 × 10^-5^ | 0.23 [0.12; 0.42] | 2.64 × 10^-8^ | 0.13 [0.06; 0.26] |
| 534.8484@7.2451415 | LysoPC (18:0) | 7.25 | 535.8557 | 2.03 × 10^-6^ | 0.21 [0.11; 0.38] | 1.34 × 10^-4^ | 0.26 [0.13; 0.48] | 1.20 × 10^-6^ | 0.16 [0.07; 0.32] |
| 550.825@7.245036 | LysoPC (18:0) | 7.25 | 551.8323 | 2.20 × 10^-5^ | 0.32 [0.2; 0.52] | 7.39 × 10^-4^ | 0.37 [0.22; 0.62] | 1.47 × 10^-5^ | 0.27 [0.15; 0.47] |
| 542.8413@7.246016 | LysoPC (18:0) | 7.25 | 543.8486 | 3.78 × 10^-8^ | 0.2 [0.12; 0.35] | 9.14 × 10^-6^ | 0.25 [0.14; 0.43] | 4.16 × 10^-8^ | 0.16 [0.08; 0.3] |
| 103.0998@7.2424936 | LysoPC (18:0) | 7.24 | 104.1071 | 9.78 × 10^-4^ | 0.22 [0.1; 0.49] | 1.55 × 10^-2^ | 0.3 [0.13; 0.69] | 3.08 × 10^-4^ | 0.14 [0.05; 0.37] |
| 525.3711@7.244797 | LysoPC (18:0) | 7.24 | 526.3784 | 3.75 × 10^-6^ | 0.19 [0.1; 0.37] | 2.08 × 10^-4^ | 0.23 [0.11; 0.46] | 3.25 × 10^-6^ | 0.15 [0.06; 0.31] |
| 281.1584@7.24184 | LysoPC (18:0) | 7.24 | 282.1657 | 2.48 × 10^-2^ | 0.42 [0.22; 0.8] | 1.42 × 10^-1^ | 0.52 [0.25; 1.06] | 6.21 × 10^-3^ | 0.3 [0.13; 0.64] |
| 289.1815@7.2417345 | LysoPC (18:0) | 7.24 | 290.1888 | 2.31 × 10^-2^ | 0.37 [0.18; 0.77] | 6.40 × 10^-2^ | 0.39 [0.16; 0.89] | 2.18 × 10^-2^ | 0.34 [0.15; 0.75] |
| 549.8097@7.0650086 | LysoPC (18:1) | 7.07 | 550.8170 | 3.89 × 10^-3^ | 0.32 [0.16; 0.64] | 3.79 × 10^-2^ | 0.39 [0.18; 0.82] | 1.39 × 10^-3^ | 0.25 [0.12; 0.54] |
| 521.3484@7.0633354 | **LysoPC (18:1)** | 7.06 | 522.3557 | 1.62 × 10^-2^ | 0.34 [0.16; 0.73] | 1.25 × 10^-1^ | 0.46 [0.2; 1.03] | 4.09 × 10^-3^ | 0.24 [0.1; 0.57] |
| 800.992@7.064609 | LysoPC (18:1) | 7.06 | 801.9993 | 2.07 × 10^-2^ | 0.43 [0.22; 0.8] | 7.51 × 10^-2^ | 0.48 [0.24; 0.93] | 1.57 × 10^-2^ | 0.38 [0.18; 0.75] |
| 532.3307@7.066129 | LysoPC (18:1) | 7.07 | 533.3380 | 1.69 × 10^-3^ | 0.27 [0.13; 0.56] | 3.56 × 10^-2^ | 0.37 [0.17; 0.81] | 3.62 × 10^-4^ | 0.19 [0.08; 0.43] |
| 549.3086@7.0653367 | LysoPC (18:1) | 7.07 | 550.3159 | 2.57 × 10^-2^ | 0.4 [0.2; 0.8] | 1.55 × 10^-1^ | 0.52 [0.25; 1.08] | 6.97 × 10^-3^ | 0.3 [0.14; 0.65] |
| 548.308@7.066111 | LysoPC (18:1) | 7.07 | 549.3153 | 4.05 × 10^-2^ | 0.41 [0.2; 0.85] | 1.51 × 10^-1^ | 0.5 [0.23; 1.08] | 2.09 × 10^-2^ | 0.35 [0.15; 0.75] |
| 532.8329@7.066005 | LysoPC (18:1) | 7.07 | 533.8402 | 9.71 × 10^-3^ | 0.33 [0.16; 0.69] | 6.05 × 10^-2^ | 0.4 [0.18; 0.89] | 4.03 × 10^-3^ | 0.25 [0.11; 0.58] |
| 540.8247@7.0657663 | LysoPC (18:1) | 7.07 | 541.8320 | 7.38 × 10^-3^ | 0.36 [0.19; 0.69] | 6.69 × 10^-2^ | 0.45 [0.22; 0.91] | 2.00 × 10^-3^ | 0.27 [0.12; 0.57] |
| 540.3222@7.065848 | LysoPC (18:1) | 7.07 | 541.3295 | 5.37 × 10^-3^ | 0.35 [0.18; 0.67] | 5.46 × 10^-2^ | 0.44 [0.21; 0.88] | 1.36 × 10^-3^ | 0.25 [0.12; 0.54] |
| 548.8092@7.0652876 | LysoPC (18:1) | 7.07 | 549.8165 | 6.62 × 10^-3^ | 0.33 [0.16; 0.67] | 7.54 × 10^-2^ | 0.44 [0.2; 0.93] | 1.76 × 10^-3^ | 0.25 [0.11; 0.55] |
| 543.3299@7.0667906 | LysoPC (18:1) | 7.07 | 544.3372 | 1.75 × 10^-2^ | 0.29 [0.12; 0.7] | 1.42 × 10^-1^ | 0.43 [0.17; 1.07] | 2.64 × 10^-3^ | 0.16 [0.06; 0.47] |
| 808.9788@7.064414 | LysoPC (18:1) | 7.06 | 809.9861 | 1.65 × 10^-1^ | 0.59 [0.32; 1.08] | 4.50 × 10^-1^ | 0.72 [0.38; 1.36] | 4.70 × 10^-2^ | 0.45 [0.22; 0.87] |
| 571.2916@6.896005 | LysoPC (20:4) | 6.90 | 572.2989 | 1.05 × 10^-3^ | 0.22 [0.1; 0.49] | 1.07 × 10^-2^ | 0.26 [0.1; 0.63] | 8.79 × 10^-4^ | 0.18 [0.07; 0.44] |
| 559.2956@6.9031916 | LysoPC (20:4) | 6.90 | 560.3029 | 7.13 × 10^-3^ | 0.25 [0.1; 0.6] | 4.65 × 10^-2^ | 0.3 [0.11; 0.81] | 4.13 × 10^-3^ | 0.18 [0.06; 0.51] |
| 559.7944@6.902563 | LysoPC (20:4) | 6.90 | 560.8017 | 2.55 × 10^-3^ | 0.19 [0.07; 0.49] | 1.10 × 10^-2^ | 0.2 [0.07; 0.58] | 3.29 × 10^-3^ | 0.16 [0.05; 0.47] |
| 550.3057@6.903302 | LysoPC (20:4) | 6.90 | 551.3130 | 5.25 × 10^-3^ | 0.32 [0.16; 0.65] | 3.31 × 10^-2^ | 0.37 [0.17; 0.8] | 3.15 × 10^-3^ | 0.25 [0.11; 0.57] |
| 542.8182@6.902509 | LysoPC (20:4) | 6.90 | 543.8255 | 1.64 × 10^-3^ | 0.14 [0.04; 0.41] | 1.91 × 10^-2^ | 0.2 [0.06; 0.63] | 4.40 × 10^-4^ | 0.06 [0.01; 0.26] |
| 550.8063@6.9031024 | LysoPC (20:4) | 6.90 | 551.8136 | 8.10 × 10^-4^ | 0.19 [0.08; 0.45] | 4.87 × 10^-3^ | 0.2 [0.07; 0.54] | 9.70 × 10^-4^ | 0.15 [0.05; 0.41] |
| 543.3329@6.89792 | **LysoPC (20:4)** | 6.90 | 544.3402 | 1.09 × 10^-4^ | 0.22 [0.11; 0.44] | 6.70 × 10^-4^ | 0.22 [0.1; 0.48] | 2.18 × 10^-4^ | 0.19 [0.08; 0.42] |
| 570.2903@6.8958497 | LysoPC (20:4) | 6.90 | 571.2976 | 3.27 × 10^-3^ | 0.29 [0.13; 0.6] | 1.61 × 10^-2^ | 0.31 [0.13; 0.7] | 3.34 × 10^-3^ | 0.25 [0.1; 0.57] |
| 558.2915@6.9029274 | LysoPC (20:4) | 6.90 | 559.2988 | 2.90 × 10^-2^ | 0.35 [0.15; 0.78] | 1.00 × 10^-1^ | 0.4 [0.16; 0.98] | 2.28 × 10^-2^ | 0.29 [0.11; 0.72] |
| 558.7932@6.902813 | LysoPC (20:4) | 6.90 | 559.8005 | 1.90 × 10^-2^ | 0.31 [0.13; 0.73] | 6.58 × 10^-2^ | 0.35 [0.14; 0.88] | 2.11 × 10^-2^ | 0.28 [0.11; 0.71] |
| 569.3465@6.967688 | **LysoPC (22:5)** | 6.97 | 570.3538 | 1.15 × 10^-2^ | 0.35 [0.17; 0.71] | 6.90 × 10^-2^ | 0.42 [0.19; 0.91] | 3.58 × 10^-3^ | 0.25 [0.11; 0.58] |
| 567.3317@6.8900876 | **LysoPC (22:6)** | 6.89 | 568.3390 | 4.92 × 10^-2^ | 0.48 [0.25; 0.89] | 1.10 × 10^-1^ | 0.51 [0.25; 1] | 4.84 × 10^-2^ | 0.43 [0.2; 0.87] |
| 479.3402@7.1128163 | **LysoPC (P-16:0)** | 7.11 | 480.3475 | 6.50 × 10^-5^ | 0.23 [0.12; 0.45] | 3.76 × 10^-3^ | 0.32 [0.16; 0.63] | 1.48 × 10^-5^ | 0.15 [0.07; 0.34] |
| 501.3191@7.11705 | LysoPC (P-16:0) | 7.12 | 502.3264 | 5.32 × 10^-5^ | 0.21 [0.1; 0.42] | 1.40 × 10^-3^ | 0.26 [0.12; 0.54] | 3.54 × 10^-5^ | 0.16 [0.07; 0.36] |
| 301.225@4.6254926 | **Nonanoylcarnitine (C9:0)** | 4.63 | 302.2323 | 1.62 × 10^-1^ | 1.32 [0.96; 1.82] | 1.47 × 10^-2^ | 1.7 [1.18; 2.48] | 8.09 × 10^-1^ | 1.07 [0.75; 1.53] |
| 287.2104@4.416547 | **Octanoylcarnitine (C8:0)** | 4.42 | 288.2177 | 9.86 × 10^-4^ | 0.46 [0.3; 0.7] | 3.51 × 10^-3^ | 0.47 [0.29; 0.74] | 3.08 × 10^-3^ | 0.46 [0.28; 0.73] |
| 804.0459@8.644213 | PC (36:4) | 8.64 | 805.0532 | 1.37 × 10^-2^ | 0.23 [0.08; 0.63] | 1.47 × 10^-2^ | 0.18 [0.05; 0.59] | 6.12 × 10^-2^ | 0.27 [0.09; 0.85] |
| 401.7651@8.650028 | PC (36:4) | 8.65 | 402.7724 | 1.26 × 10^-2^ | 0.22 [0.08; 0.62] | 6.86 × 10^-3^ | 0.16 [0.05; 0.51] | 8.34 × 10^-2^ | 0.31 [0.1; 0.93] |
| 808.5272@8.650579 | PC (36:4) | 8.65 | 809.5345 | 4.33 × 10^-2^ | 0.46 [0.24; 0.87] | 3.08 × 10^-2^ | 0.4 [0.19; 0.8] | 1.76 × 10^-1^ | 0.56 [0.28; 1.09] |
| 809.028@8.650744 | PC (36:4) | 8.65 | 810.0353 | 3.44 × 10^-2^ | 0.37 [0.17; 0.82] | 3.00 × 10^-2^ | 0.32 [0.13; 0.76] | 1.51 × 10^-1^ | 0.47 [0.2; 1.07] |
| 813.0396@8.636547 | PC (36:4) | 8.64 | 814.0469 | 4.80 × 10^-2^ | 0.3 [0.11; 0.83] | 4.65 × 10^-2^ | 0.26 [0.08; 0.79] | 1.51 × 10^-1^ | 0.37 [0.12; 1.1] |
| 812.5362@8.636105 | PC (36:4) | 8.64 | 813.5435 | 2.98 × 10^-2^ | 0.27 [0.1; 0.75] | 4.11 × 10^-2^ | 0.25 [0.08; 0.76] | 9.47 × 10^-2^ | 0.33 [0.11; 0.96] |
| 409.7539@8.648979 | PC (36:4) | 8.65 | 410.7612 | 3.37 × 10^-3^ | 0.12 [0.03; 0.42] | 3.29 × 10^-3^ | 0.09 [0.02; 0.37] | 2.42 × 10^-2^ | 0.16 [0.04; 0.63] |
| 410.256@8.647972 | PC (36:4) | 8.65 | 411.2633 | 5.80 × 10^-3^ | 0.12 [0.03; 0.45] | 5.79 × 10^-3^ | 0.09 [0.02; 0.41] | 3.32 × 10^-2^ | 0.16 [0.04; 0.68] |
| 402.2672@8.650017 | PC (36:4) | 8.65 | 403.2745 | 1.26 × 10^-2^ | 0.19 [0.06; 0.6] | 1.65 × 10^-2^ | 0.17 [0.05; 0.58] | 4.35 × 10^-2^ | 0.23 [0.07; 0.77] |
| 801.0365@8.651964 | PC (36:4) | 8.65 | 802.0438 | 1.61 × 10^-3^ | 0.18 [0.07; 0.47] | 7.41 × 10^-4^ | 0.12 [0.04; 0.36] | 2.80 × 10^-2^ | 0.27 [0.09; 0.73] |
| 800.5363@8.65209 | PC (36:4) | 8.65 | 801.5436 | 4.34 × 10^-3^ | 0.19 [0.07; 0.52] | 4.51 × 10^-3^ | 0.15 [0.04; 0.47] | 3.16 × 10^-2^ | 0.25 [0.08; 0.73] |
| 792.5527@8.653272 | PC (36:4) | 8.65 | 793.5600 | 9.53 × 10^-3^ | 0.22 [0.08; 0.6] | 1.00 × 10^-2^ | 0.18 [0.05; 0.56] | 4.97 × 10^-2^ | 0.28 [0.09; 0.81] |
| 793.0533@8.652217 | PC (36:4) | 8.65 | 794.0606 | 6.86 × 10^-4^ | 0.17 [0.07; 0.43] | 7.68 × 10^-4^ | 0.14 [0.05; 0.39] | 6.85 × 10^-3^ | 0.2 [0.07; 0.56] |
| 781.5655@8.652343 | **PC (36:4)** | 8.65 | 782.5728 | 5.87 × 10^-3^ | 0.3 [0.14; 0.64] | 4.35 × 10^-3^ | 0.24 [0.1; 0.57] | 4.18 × 10^-2^ | 0.37 [0.16; 0.83] |
| 809.5957@9.208709 | **PC (38:4)** | 9.21 | 810.6030 | 3.04 × 10^-2^ | 0.45 [0.24; 0.84] | 1.23 × 10^-2^ | 0.35 [0.17; 0.71] | 1.57 × 10^-1^ | 0.56 [0.28; 1.06] |
| 831.5749@9.208659 | PC (38:4) | 9.21 | 832.5822 | 1.06 × 10^-2^ | 0.34 [0.16; 0.7] | 3.85 × 10^-3^ | 0.24 [0.1; 0.57] | 6.45 × 10^-2^ | 0.41 [0.18; 0.9] |
| 423.7682@9.210264 | PC (38:4) | 9.21 | 424.7755 | 1.40 × 10^-3^ | 0.16 [0.05; 0.44] | 6.17 × 10^-4^ | 0.11 [0.03; 0.34] | 3.01 × 10^-2^ | 0.25 [0.08; 0.73] |
| 264.1117@3.1115255 | **Phenylacetylglutamine** | 3.11 | 265.1190 | 3.15 × 10^-24^ | 3.51 [2.71; 4.67] | 9.01 × 10^-18^ | 3.3 [2.47; 4.55] | 2.25 × 10^-20^ | 3.73 [2.74; 5.26] |
| 69.0582@0.6856087 | Proline | 0.69 | 70.0655 | 3.99 × 10^-3^ | 3.72 [1.69; 8.46] | 6.33 × 10^-3^ | 4 [1.67; 10.08] | 1.16 × 10^-2^ | 3.86 [1.54; 10.32] |
| 115.0639@0.6856974 | **Proline** | 0.69 | 116.0712 | 5.02 × 10^-3^ | 3.87 [1.69; 9.12] | 4.70 × 10^-3^ | 4.35 [1.78; 11.32] | 2.48 × 10^-2^ | 3.5 [1.37; 9.48] |
| 217.1309@1.3207631 | **Propionylcarnitine (C3:0)** | 1.32 | 218.1382 | 2.15 × 10^-5^ | 5.14 [2.56; 10.68] | 8.16 × 10^-5^ | 5.27 [2.5; 11.67] | 4.60 × 10^-4^ | 4.37 [2.06; 9.77] |
| 125.0146@0.62882745 | **Taurine** | 0.63 | 126.0219 | 6.10 × 10^-13^ | 16.17 [7.81; 35.24] | 4.63 × 10^-9^ | 11.03 [5.19; 25.08] | 2.70 × 10^-11^ | 20.87 [8.55; 56.16] |
| 367.2726@5.618519 | **Tetradecadienoylcarnitine (C14:2)** | 5.62 | 368.2799 | 5.02 × 10^-5^ | 0.39 [0.25; 0.59] | 1.05 × 10^-4^ | 0.37 [0.23; 0.58] | 1.39 × 10^-3^ | 0.43 [0.26; 0.68] |
| 371.3036@5.993787 | **Tetradecanoylcarnitine (C14:0)** | 5.99 | 372.3109 | 1.16 × 10^-4^ | 0.25 [0.13; 0.48] | 4.42 × 10^-4^ | 0.24 [0.12; 0.49] | 1.15 × 10^-3^ | 0.26 [0.12; 0.54] |
| 369.2886@5.826272 | **Tetradecenoylcarnitine (C14:1)** | 5.83 | 370.2959 | 1.46 × 10^-5^ | 0.38 [0.25; 0.57] | 5.34 × 10^-5^ | 0.37 [0.23; 0.57] | 3.25 × 10^-4^ | 0.4 [0.25; 0.63] |
| 393.2876@5.827678 | Tetradecenoylcarnitine (C14:1) | 5.83 | 394.2949 | 1.45 × 10^-7^ | 0.14 [0.07; 0.27] | 9.95 × 10^-7^ | 0.13 [0.06; 0.27] | 1.10 × 10^-5^ | 0.15 [0.06; 0.33] |
| 180.0648@2.3754191 | **Theobromine** | 2.38 | 181.0721 | 8.42 × 10^-3^ | 1.46 [1.14; 1.89] | 1.78 × 10^-2^ | 1.46 [1.12; 1.93] | 1.71 × 10^-2^ | 1.5 [1.12; 2.02] |
| 180.065@2.8075275 | **Theophylline** | 2.81 | 181.0723 | 4.20 × 10^-2^ | 1.33 [1.05; 1.71] | 1.06 × 10^-1^ | 1.29 [1; 1.67] | 3.35 × 10^-2^ | 1.44 [1.08; 1.96] |
| 117.0793@0.80347276 | **Valine** | 0.80 | 118.0866 | 2.89 × 10^-2^ | 0.21 [0.06; 0.69] | 5.58 × 10^-2^ | 0.22 [0.06; 0.8] | 6.03 × 10^-2^ | 0.21 [0.05; 0.82] |
| 71.0739@0.80347854 | Valine | 0.80 | 72.0812 | 2.73 × 10^-2^ | 0.24 [0.08; 0.71] | 4.19 × 10^-2^ | 0.24 [0.07; 0.76] | 6.96 × 10^-2^ | 0.27 [0.08; 0.87] |
| 139.0612@0.80180687 | Valine | 0.80 | 140.0685 | 1.39 × 10^-4^ | 0.2 [0.09; 0.42] | 5.75 × 10^-4^ | 0.2 [0.08; 0.45] | 1.17 × 10^-3^ | 0.2 [0.08; 0.48] |
| 264.1357@5.265392 | **γ-carbo×yethyl hydro×ychroman** | 5.27 | 265.1430 | 3.25 × 10^-2^ | 2.49 [1.22; 5.1] | 4.72 × 10^-2^ | 2.56 [1.18; 5.7] | 5.61 × 10^-2^ | 2.67 [1.15; 6.35] |
| [246.9905@0.87735534](mailto:246.9905@0.87735534) |  | 0.88 | 247.9978 | 1.76 × 10^-2^ | 0.64 [0.47; 0.88] | 1.03 × 10^-2^ | 0.6 [0.43; 0.84] | 1.72 × 10^-1^ | 0.73 [0.51; 1.05] |
| 218.0372@0.87743944 |  | 0.88 | 219.0445 | 9.50 × 10^-3^ | 0.57 [0.39; 0.82] | 8.70 × 10^-3^ | 0.54 [0.36; 0.81] | 9.41 × 10^-2^ | 0.64 [0.42; 0.98] |
| 243.9906@0.8904056 |  | 0.89 | 244.9979 | 3.25 × 10^-2^ | 0.67 [0.48; 0.92] | 1.91 × 10^-2^ | 0.62 [0.44; 0.87] | 2.36 × 10^-1^ | 0.75 [0.52; 1.08] |
| 227.9972@0.90200853 |  | 0.90 | 229.0045 | 2.19 × 10^-2^ | 0.58 [0.38; 0.87] | 3.06 × 10^-3^ | 0.46 [0.29; 0.72] | 2.91 × 10^-1^ | 0.73 [0.46; 1.14] |
| 200.9487@1.2711828 |  | 1.27 | 201.9560 | 4.80 × 10^-2^ | 0.71 [0.53; 0.95] | 1.07 × 10^-2^ | 0.62 [0.45; 0.85] | 4.07 × 10^-1^ | 0.83 [0.61; 1.15] |
| 185.9828@1.2703432 |  | 1.27 | 186.9901 | 2.91 × 10^-3^ | 0.58 [0.42; 0.8] | 1.37 × 10^-3^ | 0.53 [0.37; 0.75] | 7.35 × 10^-2^ | 0.68 [0.48; 0.97] |
| 233.0113@2.1010334 |  | 2.10 | 234.0186 | 1.74 × 10^-2^ | 0.24 [0.08; 0.67] | 1.62 × 10^-2^ | 0.21 [0.07; 0.62] | 9.24 × 10^-2^ | 0.3 [0.09; 0.95] |
| 427.3656@6.4610605 |  | 6.46 | 428.3729 | 2.50 × 10^-2^ | 0.34 [0.15; 0.77] | 4.25 × 10^-2^ | 0.33 [0.13; 0.81] | 5.97 × 10^-2^ | 0.36 [0.14; 0.88] |
| 574.3029@6.8899956 |  | 6.89 | 575.3102 | 3.39 × 10^-2^ | 0.27 [0.09; 0.76] | 4.90 × 10^-2^ | 0.25 [0.08; 0.79] | 7.69 × 10^-2^ | 0.31 [0.1; 0.91] |
| 215.0097@1.0514679 |  | 1.05 | 216.0170 | 4.80 × 10^-2^ | 0.45 [0.22; 0.88] | 2.73 × 10^-2^ | 0.35 [0.16; 0.77] | 1.99 × 10^-1^ | 0.54 [0.26; 1.13] |
| 204.0206@1.2712277 |  | 1.27 | 205.0279 | 4.61 × 10^-2^ | 0.66 [0.46; 0.93] | 1.63 × 10^-2^ | 0.58 [0.39; 0.85] | 3.11 × 10^-1^ | 0.77 [0.52; 1.13] |
| 124.0137@1.2705848 |  | 1.27 | 125.0210 | 1.47 × 10^-2^ | 0.68 [0.52; 0.89] | 7.68 × 10^-4^ | 0.55 [0.4; 0.75] | 3.40 × 10^-1^ | 0.83 [0.62; 1.11] |
| 358.1602@7.2488236 |  | 7.25 | 359.1675 | 1.47 × 10^-2^ | 0.48 [0.29; 0.8] | 1.66 × 10^-2^ | 0.45 [0.25; 0.79] | 8.20 × 10^-2^ | 0.55 [0.31; 0.96] |
| 805.0531@8.634231 |  | 8.63 | 806.0604 | 1.30 × 10^-2^ | 0.3 [0.13; 0.69] | 8.35 × 10^-3^ | 0.26 [0.1; 0.62] | 6.04 × 10^-2^ | 0.33 [0.12; 0.87] |
| 641.855@0.54527247 |  | 0.55 | 642.8623 | 4.80 × 10^-2^ | 0.42 [0.2; 0.87] | 3.08 × 10^-2^ | 0.35 [0.15; 0.78] | 1.38 × 10^-1^ | 0.48 [0.22; 1.05] |
| 811.6072@9.529143 |  | 9.53 | 812.6145 | 3.92 × 10^-2^ | 0.45 [0.23; 0.86] | 2.05 × 10^-2^ | 0.37 [0.17; 0.76] | 1.62 × 10^-1^ | 0.55 [0.27; 1.08] |
| 367.272@5.760921 |  | 5.76 | 368.2793 | 4.31 × 10^-2^ | 0.37 [0.16; 0.84] | 4.32 × 10^-2^ | 0.34 [0.13; 0.82] | 9.94 × 10^-2^ | 0.41 [0.16; 0.98] |
| 248.1002@0.6071271 |  | 0.61 | 249.1075 | 4.33 × 10^-2^ | 0.34 [0.14; 0.83] | 9.23 × 10^-2^ | 0.4 [0.16; 0.96] | 7.94 × 10^-2^ | 0.34 [0.12; 0.92] |
| 157.1107@0.8298541 |  | 0.83 | 158.1180 | 3.60 × 10^-2^ | 0.62 [0.42; 0.91] | 5.47 × 10^-2^ | 0.62 [0.41; 0.93] | 6.96 × 10^-2^ | 0.62 [0.4; 0.95] |
| 422.7614@8.752114 |  | 8.75 | 423.7687 | 3.59 × 10^-2^ | 0.48 [0.27; 0.86] | 6.40 × 10^-2^ | 0.48 [0.25; 0.91] | 6.96 × 10^-2^ | 0.5 [0.26; 0.93] |
| 430.7503@8.751049 |  | 8.75 | 431.7576 | 3.14 × 10^-2^ | 0.33 [0.14; 0.79] | 6.44 × 10^-2^ | 0.35 [0.13; 0.88] | 6.97 × 10^-2^ | 0.36 [0.14; 0.9] |
| 318.1477@6.8379445 |  | 6.84 | 319.1550 | 4.30 × 10^-2^ | 0.5 [0.28; 0.89] | 9.11 × 10^-2^ | 0.54 [0.29; 0.97] | 7.68 × 10^-2^ | 0.48 [0.24; 0.94] |
| 247.1429@0.85507876 |  | 0.86 | 248.1502 | 7.82 × 10^-2^ | 0.7 [0.49; 0.98] | 1.66 × 10^-2^ | 0.6 [0.41; 0.86] | 4.98 × 10^-1^ | 0.83 [0.56; 1.22] |
| 229.9752@0.86571616 |  | 0.87 | 230.9825 | 1.05 × 10^-1^ | 1.91 [1; 3.74] | 4.19 × 10^-2^ | 2.43 [1.19; 5.14] | 4.18 × 10^-1^ | 1.5 [0.73; 3.14] |
| 188.0004@0.8802064 |  | 0.88 | 189.0077 | 1.24 × 10^-1^ | 0.54 [0.28; 1.03] | 3.52 × 10^-2^ | 0.4 [0.19; 0.82] | 6.55 × 10^-1^ | 0.79 [0.39; 1.59] |
| 103.0192@0.8879074 |  | 0.89 | 104.0265 | 1.25 × 10^-1^ | 0.71 [0.5; 1.02] | 3.79 × 10^-2^ | 0.62 [0.42; 0.9] | 6.14 × 10^-1^ | 0.86 [0.58; 1.29] |
| 230.0112@0.8899939 |  | 0.89 | 231.0185 | 7.62 × 10^-2^ | 0.68 [0.47; 0.97] | 2.74 × 10^-2^ | 0.59 [0.39; 0.87] | 4.07 × 10^-1^ | 0.79 [0.53; 1.19] |
| 196.9783@0.899427 |  | 0.90 | 197.9856 | 5.50 × 10^-2^ | 0.65 [0.44; 0.94] | 1.03 × 10^-2^ | 0.54 [0.35; 0.81] | 4.57 × 10^-1^ | 0.81 [0.53; 1.22] |
| 202.9642@1.500623 |  | 1.50 | 203.9715 | 1.30 × 10^-1^ | 0.71 [0.49; 1.02] | 3.94 × 10^-2^ | 0.6 [0.4; 0.9] | 6.47 × 10^-1^ | 0.87 [0.57; 1.32] |
| 188.0003@1.7456483 |  | 1.75 | 189.0076 | 1.15 × 10^-1^ | 0.59 [0.34; 1.01] | 3.77 × 10^-2^ | 0.47 [0.26; 0.85] | 5.00 × 10^-1^ | 0.75 [0.41; 1.37] |
| 317.1134@0.8575037 |  | 0.86 | 318.1207 | 1.14 × 10^-1^ | 1.58 [0.99; 2.58] | 1.76 × 10^-2^ | 2.11 [1.24; 3.69] | 4.61 × 10^-1^ | 1.31 [0.78; 2.23] |
| 203.9708@0.88541883 |  | 0.89 | 204.9781 | 1.00 × 10^-1^ | 0.61 [0.38; 0.99] | 3.31 × 10^-2^ | 0.52 [0.31; 0.86] | 5.53 × 10^-1^ | 0.79 [0.46; 1.36] |
| 233.962@0.89373153 |  | 0.89 | 234.9693 | 1.06 × 10^-1^ | 0.6 [0.35; 1] | 3.91 × 10^-2^ | 0.5 [0.28; 0.87] | 5.41 × 10^-1^ | 0.77 [0.43; 1.38] |
| 187.9999@1.5010325 |  | 1.50 | 189.0072 | 1.03 × 10^-1^ | 0.66 [0.44; 1] | 3.53 × 10^-2^ | 0.57 [0.36; 0.88] | 5.38 × 10^-1^ | 0.81 [0.51; 1.3] |
| 342.1865@7.248144 |  | 7.25 | 343.1938 | 6.09 × 10^-2^ | 0.52 [0.29; 0.92] | 4.26 × 10^-2^ | 0.45 [0.23; 0.86] | 2.64 × 10^-1^ | 0.62 [0.33; 1.18] |
| 205.9733@1.5004629 |  | 1.50 | 206.9806 | 1.17 × 10^-1^ | 0.68 [0.45; 1.01] | 4.26 × 10^-2^ | 0.58 [0.37; 0.9] | 5.53 × 10^-1^ | 0.82 [0.52; 1.3] |
| 188.9841@1.5000874 |  | 1.50 | 189.9914 | 6.19 × 10^-2^ | 0.5 [0.27; 0.92] | 2.48 × 10^-2^ | 0.41 [0.2; 0.79] | 3.57 × 10^-1^ | 0.65 [0.33; 1.29] |
| 210.124@5.7608237 |  | 5.76 | 211.1313 | 6.09 × 10^-2^ | 0.42 [0.19; 0.9] | 3.08 × 10^-2^ | 0.35 [0.15; 0.78] | 3.11 × 10^-1^ | 0.55 [0.23; 1.32] |
| 204.0208@1.0509856 |  | 1.05 | 205.0281 | 7.82 × 10^-2^ | 0.59 [0.36; 0.96] | 2.87 × 10^-2^ | 0.49 [0.28; 0.84] | 3.59 × 10^-1^ | 0.72 [0.42; 1.22] |
| 701.8565@0.55352145 |  | 0.55 | 702.8638 | 4.07 × 10^-6^ | 0.37 [0.25; 0.54] | 1.12 × 10^-4^ | 0.39 [0.25; 0.6] | 4.56 × 10^-6^ | 0.3 [0.18; 0.49] |
| 633.8705@0.5587821 |  | 0.56 | 634.8778 | 5.77 × 10^-6^ | 0.35 [0.23; 0.53] | 1.35 × 10^-4^ | 0.37 [0.23; 0.58] | 6.62 × 10^-6^ | 0.28 [0.16; 0.47] |
| 853.8052@0.5577894 |  | 0.56 | 854.8125 | 1.45 × 10^-5^ | 0.28 [0.16; 0.47] | 2.96 × 10^-4^ | 0.3 [0.16; 0.54] | 1.23 × 10^-5^ | 0.2 [0.1; 0.4] |
| 785.8198@0.5599011 |  | 0.56 | 786.8271 | 1.63 × 10^-5^ | 0.26 [0.14; 0.45] | 3.32 × 10^-4^ | 0.28 [0.14; 0.52] | 1.41 × 10^-5^ | 0.18 [0.08; 0.37] |
| 717.8281@0.5620177 |  | 0.56 | 718.8354 | 1.76 × 10^-4^ | 0.29 [0.16; 0.52] | 3.50 × 10^-3^ | 0.34 [0.17; 0.64] | 2.21 × 10^-5^ | 0.16 [0.06; 0.35] |
| 227.949@0.5615746 |  | 0.56 | 228.9563 | 3.78 × 10^-5^ | 0.15 [0.07; 0.34] | 5.75 × 10^-4^ | 0.18 [0.07; 0.42] | 4.16 × 10^-5^ | 0.11 [0.04; 0.29] |
| 225.9446@0.5617323 |  | 0.56 | 226.9519 | 2.05 × 10^-5^ | 0.19 [0.09; 0.39] | 5.97 × 10^-4^ | 0.23 [0.1; 0.49] | 7.88 × 10^-6^ | 0.11 [0.04; 0.28] |
| 293.9334@0.5608217 |  | 0.56 | 294.9407 | 4.20 × 10^-5^ | 0.25 [0.13; 0.46] | 1.15 × 10^-3^ | 0.3 [0.15; 0.58] | 1.48 × 10^-5^ | 0.15 [0.06; 0.35] |
| 565.8813@0.56211406 |  | 0.56 | 566.8886 | 7.21 × 10^-6^ | 0.33 [0.21; 0.51] | 1.64 × 10^-4^ | 0.36 [0.22; 0.58] | 7.36 × 10^-6^ | 0.26 [0.15; 0.46] |
| 937.7642@0.5612744 |  | 0.56 | 938.7715 | 9.40 × 10^-5^ | 0.17 [0.08; 0.38] | 1.24 × 10^-3^ | 0.19 [0.07; 0.47] | 7.98 × 10^-5^ | 0.1 [0.03; 0.3] |
| 497.8932@0.56349546 |  | 0.56 | 498.9005 | 5.30 × 10^-5^ | 0.36 [0.22; 0.56] | 1.24 × 10^-3^ | 0.4 [0.24; 0.66] | 1.01 × 10^-5^ | 0.24 [0.12; 0.43] |
| [107.9415@0.56314164](mailto:107.9415@0.56314164) |  | 0.56 | 108.9488 | 1.99 × 10^-3^ | 3.12 × 10^+2^ [11.7; 9.72 × 10^+3^] | 6.69 × 10^-3^ | 2.19 × 10^+2^ [71.7; 8.31 × 10^+3^] | 4.64 × 10^-3^ | 331 [92.1; 1.50 × 10^+4^] |
| 649.8467@0.5635613 |  | 0.56 | 650.8540 | 8.39 × 10^-5^ | 0.26 [0.13; 0.49] | 1.90 × 10^-3^ | 0.31 [0.15; 0.6] | 2.06 × 10^-5^ | 0.14 [0.05; 0.34] |
| 173.932@0.56242305 |  | 0.56 | 174.9393 | 1.49 × 10^-2^ | 96.8 [38.1; 2.87 × 10^+3^] | 3.77 × 10^-2^ | 71.18 [2.39; 2712.09] | 3.39 × 10^-2^ | 63.8 [2.38 × 10; 2.09 × 10^+3^] |
| 105.9438@0.5637788 |  | 0.56 | 106.9511 | 3.27 × 10^-3^ | 3.57 × 10+^2^ [10.7; 1.39 × 10^+4^] | 1.22 × 10^-2^ | 2.31 × 10^+2^ [57.8; 1.17 × 10^+4^] | 7.97 × 10^-3^ | 300 [72.8; 1.55 × 10^+4^] |
| 361.9207@0.562846 |  | 0.56 | 362.9280 | 2.06 × 10^-5^ | 0.29 [0.17; 0.49] | 6.76 × 10^-4^ | 0.34 [0.18; 0.59] | 6.12 × 10^-6^ | 0.18 [0.08; 0.37] |
| 429.9061@0.56308544 |  | 0.56 | 430.9134 | 1.53 × 10^-5^ | 0.33 [0.2; 0.52] | 5.02 × 10^-4^ | 0.37 [0.21; 0.61] | 4.85 × 10^-6^ | 0.22 [0.11; 0.41] |
| 767.7976@0.5638253 |  | 0.56 | 768.8049 | 3.06 × 10^-6^ | 0.04 [0.01; 0.14] | 2.40 × 10^-4^ | 0.07 [0.02; 0.25] | 7.24 × 10^-7^ | 1.20 × 10^-2^ [1.81 × 10^-3^; 6.72 × 10^-2^] |
| 121.9172@0.5644108 |  | 0.56 | 122.9245 | 1.75 × 10^-7^ | 8.3 [4; 17.76] | 1.61 × 10^-5^ | 6.98 [3.15; 16.22] | 2.44 × 10^-7^ | 12.26 [5.01; 32.06] |
| 123.916@0.5647095 |  | 0.56 | 124.9233 | 1.64 × 10^-7^ | 7.91 [3.88; 16.61] | 1.41 × 10^-5^ | 6.73 [3.1; 15.31] | 2.57 × 10^-7^ | 11.32 [4.75; 28.72] |
| 953.7372@0.56570476 |  | 0.57 | 954.7445 | 1.99 × 10^-3^ | 11.92 [2.85; 54.28] | 3.90 × 10^-3^ | 11.81 [2.63; 59.69] | 8.05 × 10^-3^ | 9.68 [2.2; 46.18] |
| 581.8576@0.56446147 |  | 0.56 | 582.8649 | 3.20 × 10^-4^ | 0.22 [0.1; 0.47] | 5.40 × 10^-3^ | 0.28 [0.12; 0.62] | 8.54 × 10^-5^ | 0.11 [0.03; 0.31] |
| 189.9046@0.5650671 |  | 0.57 | 190.9119 | 1.73 × 10^-7^ | 7.97 [3.89; 16.76] | 1.01 × 10^-5^ | 7.17 [3.26; 16.43] | 4.18 × 10^-7^ | 10.24 [4.4; 25.14] |
| 445.8808@0.56483775 |  | 0.56 | 446.8881 | 4.32 × 10^-3^ | 0.17 [0.05; 0.51] | 3.61 × 10^-2^ | 0.24 [0.07; 0.74] | 1.64 × 10^-3^ | 0.07 [0.01; 0.33] |
| 707.7968@0.5660511 |  | 0.57 | 708.8041 | 2.28 × 10^-2^ | 7.26 [1.65; 34.44] | 4.44 × 10^-2^ | 6.86 [1.41; 36.95] | 4.35 × 10^-2^ | 6.11 [1.38; 29.44] |
| 639.8105@0.56600755 |  | 0.57 | 640.8178 | 1.76 × 10^-4^ | 9.73 [3.27; 30.35] | 4.88 × 10^-4^ | 10.75 [3.24; 38.29] | 9.61 × 10^-4^ | 8.53 [2.73; 28.08] |
| 513.8702@0.5649609 |  | 0.56 | 514.8775 | 1.41 × 10^-3^ | 0.2 [0.07; 0.49] | 1.58 × 10^-2^ | 0.26 [0.09; 0.67] | 5.31 × 10^-4^ | 0.09 [0.02; 0.33] |
| 205.8797@0.5664434 |  | 0.57 | 206.8870 | 2.45 × 10^-7^ | 3.2 [2.13; 4.89] | 1.87 × 10^-5^ | 2.95 [1.88; 4.74] | 3.47 × 10^-7^ | 4 [2.42; 6.87] |
| 783.77@0.5668032 |  | 0.57 | 784.7773 | 1.79 × 10^-6^ | 11.67 [4.53; 31.78] | 2.10 × 10^-5^ | 11.31 [4.08; 33.51] | 1.01 × 10^-5^ | 11.41 [4.16; 33.41] |
| 885.7544@0.56644404 |  | 0.57 | 886.7617 | 4.67 × 10^-4^ | 8.18 [2.78; 25.4] | 2.22 × 10^-3^ | 7.2 [2.31; 24.05] | 1.78 × 10^-3^ | 7.36 [2.37; 24.41] |
| 325.8792@0.56608695 |  | 0.57 | 326.8865 | 3.87 × 10^-7^ | 15.1 [5.7; 41.55] | 9.32 × 10^-6^ | 15.3 [5.15; 48.15] | 2.08 × 10^-6^ | 16.68 [5.65; 52.27] |
| 749.7804@0.5672262 |  | 0.57 | 750.7877 | 7.88 × 10^-7^ | 5.34 [2.87; 10.23] | 1.85 × 10^-5^ | 5.04 [2.58; 10.2] | 3.36 × 10^-6^ | 5.69 [2.87; 11.71] |
| 257.8939@0.56608593 |  | 0.57 | 258.9012 | 1.64 × 10^-7^ | 23.03 [7.77; 71.14] | 8.28 × 10^-6^ | 20.61 [6.24; 72.6] | 5.47 × 10^-7^ | 29.21 [8.48; 108.19] |
| 817.765@0.56681293 |  | 0.57 | 818.7723 | 5.77 × 10^-6^ | 5.97 [2.91; 12.75] | 7.06 × 10^-5^ | 5.6 [2.6; 12.61] | 2.28 × 10^-5^ | 6.07 [2.79; 13.86] |
| 597.8276@0.56628895 |  | 0.57 | 598.8349 | 7.00 × 10^-3^ | 12.53 [2.44; 69.26] | 1.69 × 10^-2^ | 11.3 [2; 72.84] | 2.05 × 10^-2^ | 9.59 [1.82; 56.04] |
| 461.8577@0.5663497 |  | 0.57 | 462.8650 | 9.22 × 10^-6^ | 32.3 [7.83; 141.35] | 9.66 × 10^-5^ | 30.88 [6.54; 159.36] | 4.32 × 10^-5^ | 30.9 [6.76; 153.15] |
| 673.8016@0.56608343 |  | 0.57 | 674.8089 | 1.64 × 10^-3^ | 9.31 [2.64; 34.83] | 2.66 × 10^-3^ | 10.22 [2.62; 43.86] | 6.97 × 10^-3^ | 7.75 [2.09; 30.37] |
| 715.7829@0.5670139 |  | 0.57 | 716.7902 | 6.99 × 10^-6^ | 6.21 [2.97; 13.47] | 1.05 × 10^-4^ | 5.75 [2.6; 13.26] | 2.08 × 10^-5^ | 6.47 [2.91; 15.01] |
| 341.8549@0.56718135 |  | 0.57 | 342.8622 | 1.92 × 10^-7^ | 3.84 [2.41; 6.23] | 1.19 × 10^-5^ | 3.57 [2.14; 6.12] | 3.80 × 10^-7^ | 4.65 [2.67; 8.42] |
| 529.8447@0.5663316 |  | 0.57 | 530.8520 | 6.87 × 10^-5^ | 24.12 [5.69; 108.42] | 4.49 × 10^-4^ | 23.06 [4.77; 122.41] | 3.61 × 10^-4^ | 20.81 [4.55; 102.88] |
| 393.8684@0.56622857 |  | 0.57 | 394.8757 | 2.33 × 10^-6^ | 25.38 [7.34; 92.19] | 2.89 × 10^-5^ | 26.31 [6.59; 113.37] | 1.19 × 10^-5^ | 25.72 [6.69; 106.06] |
| 613.802@0.567406 |  | 0.57 | 614.8093 | 2.16 × 10^-7^ | 4.36 [2.59; 7.51] | 9.16 × 10^-6^ | 4.11 [2.34; 7.43] | 7.29 × 10^-7^ | 4.86 [2.7; 9.04] |
| 757.7649@0.5674464 |  | 0.57 | 758.7722 | 1.27 × 10^-7^ | 4.53 [2.68; 7.87] | 5.44 × 10^-6^ | 4.33 [2.45; 7.9] | 6.28 × 10^-7^ | 4.91 [2.72; 9.18] |
| 851.7575@0.56655306 |  | 0.57 | 852.7648 | 4.04 × 10^-5^ | 13.15 [4.19; 44.07] | 3.04 × 10^-4^ | 12.09 [3.58; 44.09] | 1.53 × 10^-4^ | 12.73 [3.81; 45.73] |
| 697.7639@0.56797194 |  | 0.57 | 698.7712 | 4.60 × 10^-8^ | 2.97 [2.08; 4.33] | 4.65 × 10^-6^ | 2.81 [1.89; 4.27] | 7.88 × 10^-8^ | 3.52 [2.28; 5.61] |
| 273.8662@0.5672655 |  | 0.57 | 274.8735 | 1.95 × 10^-7^ | 4.05 [2.49; 6.71] | 1.43 × 10^-5^ | 3.67 [2.16; 6.41] | 3.51 × 10^-7^ | 4.99 [2.79; 9.26] |
| 545.8144@0.5674677 |  | 0.57 | 546.8217 | 1.58 × 10^-7^ | 4.9 [2.83; 8.67] | 9.14 × 10^-6^ | 4.51 [2.48; 8.47] | 4.08 × 10^-7^ | 5.74 [3.05; 11.24] |
| 969.7138@0.56719375 |  | 0.57 | 970.7211 | 5.43 × 10^-7^ | 4.37 [2.54; 7.69] | 1.71 × 10^-5^ | 4.06 [2.28; 7.47] | 2.84 × 10^-6^ | 4.69 [2.57; 8.89] |
| 901.7256@0.56754655 |  | 0.57 | 902.7329 | 4.91 × 10^-8^ | 3.47 [2.29; 5.35] | 2.22 × 10^-6^ | 3.36 [2.14; 5.42] | 2.62 × 10^-7^ | 3.84 [2.38; 6.37] |
| 409.8424@0.5672096 |  | 0.57 | 410.8497 | 1.80 × 10^-7^ | 4.13 [2.52; 6.87] | 1.01 × 10^-5^ | 3.84 [2.24; 6.75] | 4.18 × 10^-7^ | 4.89 [2.75; 9.02] |
| 765.7502@0.56798613 |  | 0.57 | 766.7575 | 1.07 × 10^-7^ | 2.91 [2.02; 4.23] | 6.94 × 10^-6^ | 2.75 [1.85; 4.16] | 2.20 × 10^-7^ | 3.38 [2.2; 5.35] |
| 477.8272@0.5672992 |  | 0.57 | 478.8345 | 1.86 × 10^-7^ | 4.43 [2.64; 7.58] | 1.01 × 10^-5^ | 4.09 [2.33; 7.39] | 4.67 × 10^-7^ | 5.2 [2.85; 9.83] |
| 681.7886@0.5672514 |  | 0.57 | 682.7959 | 2.33 × 10^-7^ | 4.88 [2.79; 8.73] | 1.01 × 10^-5^ | 4.58 [2.49; 8.66] | 7.42 × 10^-7^ | 5.45 [2.91; 10.56] |
| 723.7711@0.567607 |  | 0.57 | 724.7784 | 1.49 × 10^-7^ | 4.15 [2.53; 6.98] | 5.59 × 10^-6^ | 3.98 [2.33; 7.01] | 7.39 × 10^-7^ | 4.51 [2.58; 8.19] |
| 629.778@0.5679885 |  | 0.57 | 630.7853 | 1.27 × 10^-7^ | 2.65 [1.9; 3.73] | 6.30 × 10^-6^ | 2.55 [1.77; 3.75] | 3.51 × 10^-7^ | 3.06 [2.04; 4.72] |
| 833.7379@0.56776094 |  | 0.57 | 834.7452 | 3.93 × 10^-8^ | 3.19 [2.18; 4.74] | 2.55 × 10^-6^ | 3.04 [2.01; 4.7] | 1.35 × 10^-7^ | 3.6 [2.31; 5.78] |
| 289.8404@0.56782484 |  | 0.57 | 290.8477 | 1.02 × 10^-7^ | 2.47 [1.82; 3.39] | 9.16 × 10^-6^ | 2.32 [1.66; 3.32] | 1.05 × 10^-7^ | 2.97 [2.03; 4.47] |
| 561.7893@0.5679026 |  | 0.57 | 562.7966 | 7.19 × 10^-8^ | 2.73 [1.95; 3.87] | 5.98 × 10^-6^ | 2.57 [1.78; 3.79] | 1.48 × 10^-7^ | 3.15 [2.11; 4.85] |
| 493.8011@0.56768376 |  | 0.57 | 494.8084 | 2.19 × 10^-7^ | 2.45 [1.79; 3.38] | 1.55 × 10^-5^ | 2.3 [1.64; 3.3] | 3.68 × 10^-7^ | 2.83 [1.94; 4.23] |
| 425.8159@0.56779784 |  | 0.57 | 426.8232 | 2.19 × 10^-7^ | 2.45 [1.79; 3.38] | 1.55 × 10^-5^ | 2.31 [1.64; 3.31] | 4.74 × 10^-7^ | 2.8 [1.92; 4.2] |
| 151.0147@0.59636676 |  | 0.60 | 152.0220 | 1.10 × 10^-5^ | 51.8 [10.32; 275.52] | 2.22 × 10^-4^ | 42.52 [7.14; 281.35] | 1.03 × 10^-5^ | 94.11 [14.27; 705.85] |
| 135.0417@0.59815395 |  | 0.60 | 136.0490 | 1.43 × 10^-2^ | 0.16 [0.05; 0.57] | 4.32 × 10^-2^ | 0.2 [0.05; 0.74] | 2.69 × 10^-2^ | 0.16 [0.04; 0.64] |
| 218.0206@0.6400447 |  | 0.64 | 219.0279 | 1.86 × 10^-3^ | 5.8 [2.16; 15.94] | 3.76 × 10^-3^ | 5.99 [2.06; 18.24] | 8.00 × 10^-3^ | 5.44 [1.81; 17.08] |
| 137.0478@0.6713371 |  | 0.67 | 138.0551 | 2.25 × 10^-6^ | 1.91 [1.49; 2.45] | 5.47 × 10^-5^ | 1.83 [1.41; 2.41] | 1.80 × 10^-5^ | 1.92 [1.46; 2.58] |
| 186.0516@0.7111482 |  | 0.71 | 187.0589 | 9.27 × 10^-5^ | 0.32 [0.19; 0.54] | 3.72 × 10^-4^ | 0.31 [0.17; 0.55] | 3.36 × 10^-4^ | 0.3 [0.16; 0.54] |
| 181.0957@0.7162762 |  | 0.72 | 182.1030 | 8.74 × 10^-4^ | 0.33 [0.18; 0.59] | 1.38 × 10^-3^ | 0.31 [0.16; 0.58] | 4.36 × 10^-3^ | 0.33 [0.17; 0.65] |
| 203.1157@0.7192015 |  | 0.72 | 204.1230 | 2.28 × 10^-8^ | 0.1 [0.04; 0.2] | 3.44 × 10^-7^ | 0.09 [0.04; 0.21] | 9.29 × 10^-7^ | 0.09 [0.04; 0.23] |
| 190.0061@0.83479875 |  | 0.83 | 191.0134 | 1.07 × 10^-7^ | 0.08 [0.03; 0.19] | 1.97 × 10^-6^ | 0.08 [0.03; 0.2] | 4.32 × 10^-6^ | 0.08 [0.03; 0.23] |
| 112.0265@0.869753 |  | 0.87 | 113.0338 | 8.16 × 10^-10^ | 0.08 [0.03; 0.17] | 3.44 × 10^-7^ | 0.09 [0.04; 0.22] | 2.44 × 10^-8^ | 0.07 [0.03; 0.17] |
| 217.1316@0.8589906 |  | 0.86 | 218.1389 | 1.36 × 10^-5^ | 5.31 [2.66; 10.96] | 4.03 × 10^-5^ | 5.65 [2.68; 12.55] | 3.85 × 10^-4^ | 4.45 [2.1; 9.9] |
| 270.0847@0.8593351 |  | 0.86 | 271.0920 | 4.38 × 10^-4^ | 4.14 [2.03; 8.66] | 1.79 × 10^-3^ | 3.7 [1.79; 7.96] | 1.79 × 10^-3^ | 4.46 [1.93; 10.94] |
| 166.0494@0.8817869 |  | 0.88 | 167.0567 | 5.15 × 10^-3^ | 1.65 [1.21; 2.25] | 1.58 × 10^-2^ | 1.63 [1.16; 2.32] | 4.03 × 10^-3^ | 1.78 [1.26; 2.57] |
| 218.9948@1.2715683 |  | 1.27 | 220.0021 | 2.41 × 10^-3^ | 0.59 [0.44; 0.8] | 1.24 × 10^-3^ | 0.55 [0.39; 0.76] | 4.74 × 10^-2^ | 0.67 [0.48; 0.94] |
| 112.0268@1.3183482 |  | 1.32 | 113.0341 | 2.92 × 10^-11^ | 0.03 [0.01; 0.09] | 3.44 × 10^-7^ | 0.06 [0.02; 0.16] | 4.28 × 10^-11^ | 0.02 [0.01; 0.06] |
| 316.1113@3.31617 |  | 3.32 | 317.1186 | 4.34 × 10^-3^ | 0.3 [0.14; 0.63] | 2.88 × 10^-3^ | 0.26 [0.11; 0.57] | 4.05 × 10^-2^ | 0.37 [0.16; 0.83] |
| 214.0175@3.3190541 |  | 3.32 | 215.0248 | 4.95 × 10^-4^ | 0.2 [0.09; 0.45] | 8.79 × 10^-4^ | 0.18 [0.07; 0.44] | 3.76 × 10^-3^ | 0.2 [0.08; 0.53] |
| 200.0362@3.319126 |  | 3.32 | 201.0435 | 1.06 × 10^-4^ | 0.2 [0.09; 0.42] | 3.32 × 10^-4^ | 0.19 [0.08; 0.43] | 1.17 × 10^-3^ | 0.19 [0.08; 0.47] |
| 282.0348@3.3188689 |  | 3.32 | 283.0421 | 8.52 × 10^-4^ | 0.19 [0.08; 0.46] | 1.37 × 10^-3^ | 0.18 [0.07; 0.45] | 6.85 × 10^-3^ | 0.21 [0.07; 0.57] |
| 231.9885@3.3191657 |  | 3.32 | 232.9958 | 6.19 × 10^-4^ | 0.2 [0.09; 0.46] | 1.79 × 10^-3^ | 0.21 [0.08; 0.5] | 3.44 × 10^-3^ | 0.2 [0.07; 0.52] |
| 247.0286@3.3190794 |  | 3.32 | 248.0359 | 2.99 × 10^-4^ | 0.22 [0.1; 0.46] | 9.87 × 10^-4^ | 0.23 [0.1; 0.49] | 2.00 × 10^-3^ | 0.23 [0.09; 0.53] |
| 246.0421@3.3193731 |  | 3.32 | 247.0494 | 1.25 × 10^-3^ | 0.24 [0.11; 0.52] | 1.90 × 10^-3^ | 0.22 [0.09; 0.51] | 1.29 × 10^-2^ | 0.28 [0.12; 0.67] |
| 232.0558@3.3190646 |  | 3.32 | 233.0631 | 1.43 × 10^-4^ | 0.18 [0.08; 0.4] | 5.85 × 10^-4^ | 0.18 [0.07; 0.43] | 1.05 × 10^-3^ | 0.17 [0.06; 0.44] |
| 303.2049@3.5854893 |  | 3.59 | 304.2122 | 7.12 × 10^-4^ | 0.4 [0.25; 0.64] | 1.67 × 10^-3^ | 0.38 [0.22; 0.65] | 3.76 × 10^-3^ | 0.42 [0.25; 0.71] |
| 303.205@3.7314339 |  | 3.73 | 304.2123 | 3.46 × 10^-5^ | 0.3 [0.18; 0.51] | 1.66 × 10^-4^ | 0.29 [0.16; 0.52] | 2.19 × 10^-4^ | 0.3 [0.17; 0.54] |
| 285.1934@4.155865 |  | 4.16 | 286.2007 | 7.00 × 10^-3^ | 0.4 [0.23; 0.72] | 1.06 × 10^-2^ | 0.38 [0.19; 0.72] | 1.50 × 10^-2^ | 0.4 [0.21; 0.76] |
| 331.2357@4.595119 |  | 4.60 | 332.2430 | 3.73 × 10^-5^ | 0.35 [0.22; 0.55] | 1.35 × 10^-4^ | 0.33 [0.19; 0.55] | 3.85 × 10^-4^ | 0.37 [0.22; 0.61] |
| 331.2365@4.688282 |  | 4.69 | 332.2438 | 7.12 × 10^-4^ | 0.33 [0.18; 0.59] | 9.10 × 10^-4^ | 0.29 [0.14; 0.55] | 4.36 × 10^-3^ | 0.36 [0.19; 0.67] |
| 273.1763@5.228854 |  | 5.23 | 274.1836 | 5.39 × 10^-5^ | 0.26 [0.14; 0.47] | 4.49 × 10^-4^ | 0.27 [0.13; 0.52] | 1.68 × 10^-4^ | 0.24 [0.12; 0.48] |
| 339.2406@5.2408757 |  | 5.24 | 340.2479 | 1.05 × 10^-2^ | 0.4 [0.21; 0.74] | 1.42 × 10^-2^ | 0.37 [0.19; 0.73] | 3.16 × 10^-2^ | 0.42 [0.21; 0.83] |
| 341.2571@5.4095244 |  | 5.41 | 342.2644 | 4.40 × 10^-5^ | 0.44 [0.3; 0.63] | 1.12 × 10^-4^ | 0.42 [0.28; 0.63] | 9.61 × 10^-4^ | 0.47 [0.3; 0.7] |
| 301.2068@5.67005 |  | 5.67 | 302.2141 | 2.57 × 10^-4^ | 0.3 [0.17; 0.54] | 5.31 × 10^-3^ | 0.36 [0.19; 0.67] | 1.47 × 10^-4^ | 0.23 [0.11; 0.46] |
| 423.335@6.1844697 |  | 6.18 | 424.3423 | 1.79 × 10^-3^ | 0.32 [0.17; 0.61] | 2.23 × 10^-3^ | 0.31 [0.15; 0.61] | 1.92 × 10^-2^ | 0.36 [0.16; 0.75] |
| 399.3354@6.2586904 |  | 6.26 | 400.3427 | 2.24 × 10^-3^ | 0.25 [0.11; 0.55] | 4.87 × 10^-3^ | 0.24 [0.1; 0.58] | 1.16 × 10^-2^ | 0.27 [0.11; 0.66] |
| 425.3515@6.320815 |  | 6.32 | 426.3588 | 4.67 × 10^-4^ | 0.26 [0.13; 0.51] | 8.71 × 10^-4^ | 0.25 [0.12; 0.52] | 6.97 × 10^-3^ | 0.31 [0.14; 0.65] |
| 562.8041@6.8966804 |  | 6.90 | 563.8114 | 6.70 × 10^-4^ | 0.2 [0.08; 0.46] | 3.19 × 10^-3^ | 0.21 [0.08; 0.52] | 1.05 × 10^-3^ | 0.17 [0.06; 0.44] |
| 477.2846@6.904713 |  | 6.90 | 478.2919 | 5.53 × 10^-5^ | 3.48 [2; 6.17] | 3.04 × 10^-4^ | 3.4 [1.88; 6.34] | 5.16 × 10^-4^ | 3.34 [1.81; 6.38] |
| 535.2936@6.929498 |  | 6.93 | 536.3009 | 8.72 × 10^-4^ | 0.32 [0.17; 0.59] | 7.20 × 10^-3^ | 0.37 [0.19; 0.7] | 1.29 × 10^-3^ | 0.3 [0.15; 0.58] |
| 526.807@6.930444 |  | 6.93 | 527.8143 | 1.12 × 10^-2^ | 0.46 [0.27; 0.78] | 3.55 × 10^-2^ | 0.49 [0.27; 0.85] | 1.25 × 10^-2^ | 0.42 [0.23; 0.76] |
| 534.2887@6.92965 |  | 6.93 | 535.2960 | 8.55 × 10^-3^ | 0.46 [0.27; 0.76] | 4.90 × 10^-2^ | 0.51 [0.29; 0.89] | 6.85 × 10^-3^ | 0.41 [0.23; 0.72] |
| 276.1294@6.995517 |  | 7.00 | 277.1367 | 3.73 × 10^-3^ | 0.25 [0.11; 0.57] | 2.61 × 10^-2^ | 0.29 [0.11; 0.72] | 1.02 × 10^-3^ | 0.14 [0.04; 0.4] |
| 527.8139@7.053293 |  | 7.05 | 528.8212 | 9.46 × 10^-7^ | 0.09 [0.04; 0.22] | 1.31 × 10^-4^ | 0.13 [0.05; 0.33] | 5.28 × 10^-7^ | 0.06 [0.02; 0.16] |
| 479.3001@7.0532403 |  | 7.05 | 480.3074 | 2.01 × 10^-5^ | 3.6 [2.1; 6.34] | 1.65 × 10^-4^ | 3.35 [1.9; 6.13] | 1.60 × 10^-4^ | 3.64 [1.97; 7] |
| 376.2597@7.0961695 |  | 7.10 | 377.2670 | 1.89 × 10^-4^ | 0.54 [0.39; 0.73] | 1.95 × 10^-3^ | 0.58 [0.41; 0.79] | 2.81 × 10^-4^ | 0.51 [0.35; 0.72] |
| 618.9192@7.1828594 |  | 7.18 | 619.9265 | 3.69 × 10^-4^ | 0.26 [0.13; 0.5] | 4.70 × 10^-3^ | 0.3 [0.14; 0.62] | 3.37 × 10^-4^ | 0.21 [0.1; 0.46] |
| 602.9413@7.184656 |  | 7.18 | 603.9486 | 3.02 × 10-6 | 0.11 [0.04; 0.25] | 1.56 × 10^-4^ | 0.13 [0.05; 0.34] | 2.76 × 10^-6^ | 0.07 [0.02; 0.21] |
| 596.9183@7.185249 |  | 7.19 | 597.9256 | 7.19 × 10-8 | 1.63 × 10^-2^ [3.72 × 10^-3^; 6.61 × 10^-2^] | 5.98 × 10^-6^ | 2.21 × 10^-2^ [4.49 × 10^-3^; 9.84 × 10^-2^] | 2.86 × 10^-7^ | 1.14 × 10^-2^ [1.99 × 10^-3^; 5.75 × 10^-2^] |
| 597.4187@7.1853304 |  | 7.19 | 598.4260 | 7.10 × 10-7 | 0.03 [0.01; 0.11] | 2.89 × 10^-5^ | 0.04 [0.01; 0.15] | 3.87 × 10^-6^ | 2.15 × 10^-2^ [4.12 × 10^-3^; 1.00 × 10^-1^] |
| 610.9343@7.1841197 |  | 7.18 | 611.9416 | 4.49 × 10-7 | 0.12 [0.05; 0.25] | 4.63 × 10^-5^ | 0.15 [0.06; 0.34] | 6.85 × 10^-7^ | 0.09 [0.03; 0.22] |
| 611.4357@7.1842604 |  | 7.18 | 612.4430 | 6.52 × 10-7 | 0.12 [0.05; 0.26] | 4.91 × 10^-5^ | 0.15 [0.06; 0.34] | 1.20 × 10^-6^ | 0.09 [0.03; 0.23] |
| 437.8026@7.225684 |  | 7.23 | 438.8099 | 1.88 × 10-5 | 0.12 [0.05; 0.29] | 6.17 × 10^-4^ | 0.15 [0.06; 0.39] | 1.41 × 10^-5^ | 0.08 [0.03; 0.24] |
| 445.7941@7.2278624 |  | 7.23 | 446.8014 | 2.04 × 10-5 | 0.17 [0.08; 0.35] | 8.11 × 10^-4^ | 0.21 [0.09; 0.47] | 7.86 × 10^-6^ | 0.11 [0.04; 0.27] |
| 304.24@7.24821 |  | 7.25 | 305.2473 | 1.08 × 10-4 | 0.28 [0.15; 0.51] | 1.15 × 10^-4^ | 0.24 [0.12; 0.46] | 4.95 × 10^-3^ | 0.35 [0.18; 0.67] |
| 545.3463@7.2604136 |  | 7.26 | 546.3536 | 2.16 × 10-7 | 0.19 [0.1; 0.34] | 2.69 × 10^-5^ | 0.23 [0.12; 0.42] | 4.66 × 10^-7^ | 0.14 [0.06; 0.29] |
| 458.7995@7.2591653 |  | 7.26 | 459.8068 | 1.25 × 10-2 | 0.46 [0.26; 0.78] | 4.70 × 10^-2^ | 0.51 [0.28; 0.89] | 1.17 × 10^-2^ | 0.42 [0.22; 0.76] |
| 420.7772@7.27109 |  | 7.27 | 421.7845 | 5.32 × 10-6 | 0.2 [0.1; 0.37] | 4.34 × 10^-5^ | 0.18 [0.09; 0.38] | 4.84 × 10^-5^ | 0.19 [0.09; 0.4] |
| 638.9663@7.4274282 |  | 7.43 | 639.9736 | 5.43 × 10-7 | 0.07 [0.03; 0.2] | 4.52 × 10^-5^ | 0.1 [0.03; 0.28] | 8.21 × 10^-7^ | 0.05 [0.01; 0.16] |
| 639.4673@7.427779 |  | 7.43 | 640.4746 | 3.83 × 10-6 | 0.08 [0.03; 0.22] | 1.21 × 10^-4^ | 0.1 [0.03; 0.29] | 4.17 × 10^-6^ | 0.05 [0.01; 0.17] |
| 807.5765@8.747084 |  | 8.75 | 808.5838 | 6.70 × 10-4 | 0.27 [0.13; 0.53] | 3.69 × 10^-3^ | 0.28 [0.13; 0.6] | 1.46 × 10^-3^ | 0.25 [0.11; 0.54] |
| 829.5577@8.758253 |  | 8.76 | 830.5650 | 3.73 × 10-5 | 0.14 [0.06; 0.33] | 2.03 × 10^-4^ | 0.13 [0.05; 0.35] | 1.95 × 10^-4^ | 0.14 [0.05; 0.35] |
| 837.8286@0.5463693 |  | 0.55 | 838.8359 | 1.83 × 10-6 | 0.33 [0.21; 0.5] | 1.37 × 10^-4^ | 0.38 [0.24; 0.59] | 4.66 × 10^-7^ | 0.24 [0.13; 0.4] |
| 157.9575@0.5464849 |  | 0.55 | 158.9648 | 3.41 × 10-5 | 0.11 [0.04; 0.29] | 8.09 × 10^-4^ | 0.14 [0.04; 0.39] | 1.17 × 10^-5^ | 0.05 [0.01; 0.18] |
| 615.8459@0.557057 |  | 0.56 | 616.8532 | 3.26 × 10-3 | 0.3 [0.15; 0.6] | 4.93 × 10^-3^ | 0.29 [0.13; 0.61] | 4.41 × 10^-3^ | 0.27 [0.12; 0.6] |
| 793.8023@0.5585004 |  | 0.56 | 794.8096 | 9.94 × 10-6 | 0.21 [0.11; 0.39] | 2.46 × 10^-4^ | 0.24 [0.12; 0.47] | 2.88 × 10^-6^ | 0.14 [0.06; 0.3] |
| 683.8347@0.55725366 |  | 0.56 | 684.8420 | 6.33 × 10-3 | 0.41 [0.24; 0.71] | 1.04 × 10^-2^ | 0.4 [0.21; 0.73] | 5.51 × 10^-3^ | 0.37 [0.2; 0.68] |
| 921.7903@0.5543865 |  | 0.55 | 922.7976 | 1.03 × 10-5 | 0.26 [0.15; 0.44] | 1.47 × 10^-4^ | 0.27 [0.14; 0.49] | 1.01 × 10^-5^ | 0.2 [0.1; 0.39] |
| 827.7969@0.5564553 |  | 0.56 | 828.8042 | 2.49 × 10-6 | 0.2 [0.11; 0.37] | 5.16 × 10^-5^ | 0.21 [0.11; 0.41] | 2.29 × 10^-6^ | 0.15 [0.07; 0.31] |
| 589.842@0.56204206 |  | 0.56 | 590.8493 | 2.28 × 10-3 | 0.19 [0.07; 0.49] | 1.98 × 10^-2^ | 0.25 [0.09; 0.68] | 9.35 × 10^-4^ | 0.11 [0.03; 0.36] |
| 691.8233@0.559928 |  | 0.56 | 692.8306 | 1.70 × 10-5 | 0.2 [0.1; 0.39] | 6.21 × 10^-4^ | 0.25 [0.12; 0.5] | 3.36 × 10^-6^ | 0.11 [0.05; 0.27] |
| 657.827@0.5602297 |  | 0.56 | 658.8343 | 4.20 × 10-5 | 0.22 [0.11; 0.42] | 4.09 × 10^-4^ | 0.22 [0.1; 0.46] | 5.72 × 10^-5^ | 0.17 [0.07; 0.37] |
| 725.8148@0.5592785 |  | 0.56 | 726.8221 | 3.52 × 10-5 | 0.23 [0.12; 0.43] | 1.00 × 10^-3^ | 0.28 [0.14; 0.55] | 4.24 × 10^-6^ | 0.13 [0.06; 0.29] |
| 759.8117@0.5585613 |  | 0.56 | 760.8190 | 1.38 × 10-5 | 0.22 [0.12; 0.41] | 2.03 × 10^-4^ | 0.24 [0.12; 0.47] | 7.08 × 10^-6^ | 0.16 [0.07; 0.33] |
| 623.8351@0.56067204 |  | 0.56 | 624.8424 | 1.23 × 10-4 | 0.21 [0.1; 0.43] | 1.42 × 10^-3^ | 0.23 [0.1; 0.51] | 9.69 × 10^-5^ | 0.14 [0.05; 0.35] |
| 903.7694@0.5610597 |  | 0.56 | 904.7767 | 3.72 × 10-5 | 0.1 [0.04; 0.27] | 1.05 × 10^-4^ | 0.08 [0.02; 0.26] | 9.29 × 10^-5^ | 0.08 [0.02; 0.25] |
| 835.783@0.563167 |  | 0.56 | 836.7903 | 7.86 × 10-4 | 0.15 [0.06; 0.4] | 2.07 × 10^-3^ | 0.12 [0.03; 0.4] | 1.78 × 10^-3^ | 0.13 [0.04; 0.41] |
| 183.0887@0.5907283 |  | 0.59 | 184.0960 | 1.19 × 10-4 | 0.15 [0.06; 0.36] | 2.09 × 10^-3^ | 0.18 [0.07; 0.47] | 2.15 × 10^-4^ | 0.13 [0.05; 0.35] |
| 105.079@0.6018597 |  | 0.60 | 106.0863 | 1.07 × 10-7 | 8.18 [3.95; 17.83] | 1.40 × 10^-5^ | 6.53 [2.99; 15.3] | 4.67 × 10^-7^ | 9.85 [4.16; 25.52] |
| 264.0757@0.6024589 |  | 0.60 | 265.0830 | 5.75 × 10-4 | 10.63 [3.15; 37.4] | 4.47 × 10^-3^ | 8.75 [2.36; 34.55] | 4.17 × 10^-4^ | 15.34 [3.87; 65.54] |
| 232.9755@0.63711 |  | 0.64 | 233.9828 | 1.04 × 10-2 | 0.07 [0.01; 0.41] | 2.98 × 10^-2^ | 0.07 [0.01; 0.53] | 1.16 × 10^-2^ | 0.05 [0.01; 0.4] |
| 85.0527@0.88575214 |  | 0.89 | 86.0600 | 4.43 × 10-3 | 3.59 [1.65; 8.18] | 1.78 × 10^-2^ | 3.38 [1.42; 8.84] | 5.11 × 10^-3^ | 4.58 [1.76; 13.28] |
| 99.0684@0.85990757 |  | 0.86 | 100.0757 | 8.63 × 10-5 | 2.3 [1.58; 3.38] | 4.47 × 10^-3^ | 1.93 [1.3; 2.91] | 2.07 × 10^-5^ | 2.94 [1.85; 4.86] |
| 154.0742@0.8602932 |  | 0.86 | 155.0815 | 2.31 × 10-3 | 2.76 [1.55; 4.95] | 1.91 × 10^-2^ | 2.4 [1.29; 4.54] | 2.64 × 10^-3^ | 3.12 [1.61; 6.25] |
| 131.059@0.8711087 |  | 0.87 | 132.0663 | 6.86 × 10-4 | 8.81 [2.83; 28.58] | 4.93 × 10^-3^ | 7.16 [2.14; 25.5] | 2.64 × 10^-3^ | 8.42 [2.44; 31.29] |
| 154.0741@1.0227113 |  | 1.02 | 155.0814 | 1.45 × 10-2 | 2.4 [1.30; 4.54] | 2.99 × 10^-2^ | 2.31 [1.22; 4.51] | 4.39 × 10^-2^ | 2.32 [1.16; 4.81] |
| 481.3525@7.167896 |  | 7.17 | 482.3598 | 1.46 × 10-3 | 0.39 [0.23; 0.66] | 1.69 × 10^-2^ | 0.46 [0.26; 0.79] | 8.67 × 10^-4^ | 0.32 [0.17; 0.58] |
| 229.9911@3.319397 |  | 3.32 | 230.9984 | 5.49 × 10-4 | 0.31 [0.17; 0.56] | 8.11 × 10^-4^ | 0.28 [0.14; 0.55] | 5.51 × 10^-3^ | 0.34 [0.17; 0.67] |
| 261.0059@3.3191736 |  | 3.32 | 262.0132 | 2.32 × 10-3 | 0.3 [0.15; 0.6] | 6.22 × 10^-3^ | 0.31 [0.15; 0.65] | 1.01 × 10^-2^ | 0.31 [0.14; 0.68] |
| 250.0351@3.3192677 |  | 3.32 | 251.0424 | 1.77 × 10-4 | 0.15 [0.06; 0.37] | 6.17 × 10^-4^ | 0.15 [0.06; 0.39] | 1.33 × 10^-3^ | 0.15 [0.05; 0.43] |
| 264.0146@3.3188934 |  | 3.32 | 265.0219 | 7.53 × 10-4 | 0.23 [0.11; 0.5] | 1.21 × 10^-3^ | 0.21 [0.09; 0.49] | 7.65 × 10^-3^ | 0.27 [0.11; 0.63] |
| 304.9786@3.6761088 |  | 3.68 | 305.9859 | 2.92 × 10-18 | 3.46 [2.58; 4.79] | 8.32 × 10^-13^ | 3.16 [2.3; 4.52] | 3.66 × 10^-17^ | 4.02 [2.82; 6.01] |
| 565.3125@6.89252 |  | 6.89 | 566.3198 | 7.40 × 10-5 | 0.17 [0.07; 0.38] | 3.48 × 10^-4^ | 0.16 [0.06; 0.39] | 2.05 × 10^-4^ | 0.15 [0.05; 0.37] |
| 299.1912@5.4630613 |  | 5.46 | 300.1985 | 7.29 × 10-5 | 0.23 [0.11; 0.44] | 7.32 × 10^-4^ | 0.24 [0.11; 0.5] | 2.30 × 10^-4^ | 0.21 [0.09; 0.45] |
| 523.2692@6.8943877 |  | 6.89 | 524.2765 | 8.10 × 10-4 | 5.62 [2.24; 14.73] | 8.09 × 10^-3^ | 4.76 [1.73; 13.84] | 7.66 × 10^-4^ | 6.48 [2.42; 18.5] |
| 526.3025@6.92981 |  | 6.93 | 527.3098 | 1.02 × 10-2 | 0.41 [0.22; 0.74] | 4.35 × 10^-2^ | 0.47 [0.25; 0.87] | 1.16 × 10^-2^ | 0.37 [0.19; 0.73] |
| 534.7938@6.9291778 |  | 6.93 | 535.8011 | 1.11 × 10-2 | 0.43 [0.24; 0.76] | 4.92 × 10^-2^ | 0.48 [0.26; 0.88] | 8.22 × 10^-3^ | 0.39 [0.2; 0.72] |
| 535.2999@7.050965 |  | 7.05 | 536.3072 | 6.62 × 10-3 | 0.31 [0.15; 0.65] | 4.74 × 10^-2^ | 0.39 [0.18; 0.85] | 2.73 × 10^-3^ | 0.22 [0.09; 0.53] |
| 527.3117@7.052803 |  | 7.05 | 528.3190 | 3.87 × 10-7 | 0.08 [0.03; 0.2] | 7.69 × 10^-5^ | 0.12 [0.04; 0.31] | 1.84 × 10^-7^ | 0.05 [0.01; 0.14] |
| 535.8008@7.052626 |  | 7.05 | 536.8081 | 1.01 × 10-4 | 0.14 [0.06; 0.35] | 3.95 × 10^-3^ | 0.2 [0.08; 0.52] | 4.12 × 10^-5^ | 0.1 [0.03; 0.27] |
| 535.2997@7.0516157 |  | 7.05 | 536.3070 | 3.65 × 10-4 | 0.25 [0.12; 0.5] | 7.46 × 10^-3^ | 0.31 [0.15; 0.66] | 1.03 × 10^-4^ | 0.17 [0.07; 0.39] |
| 371.3032@7.0961027 |  | 7.10 | 372.3105 | 4.69 × 10-4 | 0.54 [0.38; 0.75] | 2.95 × 10^-3^ | 0.57 [0.39; 0.79] | 7.63 × 10^-4^ | 0.52 [0.35; 0.74] |
| 278.2245@7.1351433 |  | 7.14 | 279.2318 | 6.22 × 10-3 | 0.48 [0.3; 0.76] | 3.39 × 10^-2^ | 0.53 [0.32; 0.87] | 8.02 × 10^-3^ | 0.42 [0.23; 0.74] |
| 300.195@7.135452 |  | 7.14 | 301.2023 | 3.73 × 10-3 | 0.42 [0.25; 0.71] | 5.79 × 10^-3^ | 0.41 [0.23; 0.71] | 2.40 × 10^-2^ | 0.46 [0.25; 0.82] |
| 619.4193@7.182775 |  | 7.18 | 620.4266 | 7.12 × 10-4 | 0.24 [0.11; 0.51] | 6.78 × 10^-3^ | 0.28 [0.12; 0.63] | 9.20 × 10^-4^ | 0.21 [0.09; 0.48] |
| 446.2942@7.2276435 |  | 7.23 | 447.3015 | 1.90 × 10-4 | 0.2 [0.1; 0.43] | 2.09 × 10^-3^ | 0.22 [0.09; 0.51] | 1.36 × 10^-4^ | 0.15 [0.06; 0.37] |
| 432.7758@7.2483377 |  | 7.25 | 433.7831 | 1.10 × 10-7 | 0.1 [0.05; 0.22] | 3.45 × 10^-6^ | 0.09 [0.04; 0.23] | 2.06 × 10^-6^ | 0.1 [0.04; 0.25] |
| 428.7617@7.270025 |  | 7.27 | 429.7690 | 2.64 × 10-5 | 0.22 [0.11; 0.42] | 2.71 × 10^-4^ | 0.23 [0.11; 0.46] | 1.91 × 10^-4^ | 0.22 [0.1; 0.46] |
| 421.2792@7.2709484 |  | 7.27 | 422.2865 | 4.15 × 10-6 | 0.18 [0.09; 0.35] | 8.31 × 10^-5^ | 0.19 [0.09; 0.4] | 1.48 × 10^-5^ | 0.15 [0.07; 0.34] |
| 412.7854@7.270736 |  | 7.27 | 413.7927 | 3.09 × 10-5 | 0.17 [0.08; 0.36] | 1.62 × 10^-4^ | 0.15 [0.06; 0.36] | 1.77 × 10^-4^ | 0.15 [0.06; 0.37] |
| 368.203@7.317135 |  | 7.32 | 369.2103 | 1.65 × 10-3 | 0.32 [0.16; 0.6] | 5.09 × 10^-3^ | 0.33 [0.16; 0.65] | 5.30 × 10^-3^ | 0.3 [0.14; 0.63] |
| 423.2628@8.7521 |  | 8.75 | 424.2701 | 1.47 × 10-2 | 0.35 [0.17; 0.72] | 3.78 × 10^-2^ | 0.35 [0.15; 0.8] | 2.10 × 10^-2^ | 0.32 [0.14; 0.74] |
| 89.9699@0.5400047 |  | 0.54 | 90.9772 | 9.70 × 10-6 | 0.06 [0.02; 0.2] | 3.54 × 10^-4^ | 0.09 [0.02; 0.29] | 3.31 × 10^-6^ | 2.48 × 10^-2^ [4.82 × 10^-3^; 1.11 × 10^-1^] |
| 769.8461@0.5492603 |  | 0.55 | 770.8534 | 1.56 × 10-5 | 0.37 [0.24; 0.56] | 3.18 × 10^-4^ | 0.4 [0.25; 0.62] | 7.09 × 10^-6^ | 0.29 [0.17; 0.48] |
| 175.9297@0.5621122 |  | 0.56 | 176.9370 | 5.67 × 10-3 | 1.77 × 10^+2^ [6.76 × 10; 5.39 × 10^+3^] | 1.50 × 10^-2^ | 1.31 × 10^+2^ [43.9; 4.88 × 10^+3^] | 1.53 × 10^-2^ | 122 [41.0; 4.45 × 10^+3^] |
| 143.0166@3.3189692 |  | 3.32 | 144.0239 | 4.21 × 10-4 | 0.17 [0.07; 0.42] | 2.08 × 10^-3^ | 0.19 [0.07; 0.48] | 1.39 × 10^-3^ | 0.15 [0.05; 0.43] |
| 365.2562@5.3964787 |  | 5.40 | 366.2635 | 4.04 × 10-5 | 0.3 [0.17; 0.51] | 6.74 × 10^-4^ | 0.33 [0.18; 0.58] | 5.96 × 10^-5^ | 0.26 [0.13; 0.47] |
| 391.2719@5.63853 |  | 5.64 | 392.2792 | 6.47 × 10-3 | 0.31 [0.14; 0.65] | 2.31 × 10^-2^ | 0.33 [0.14; 0.74] | 8.24 × 10^-3^ | 0.29 [0.13; 0.65] |
| 387.2983@5.7734385 |  | 5.77 | 388.3056 | 1.37 × 10-3 | 0.23 [0.1; 0.51] | 2.59 × 10^-3^ | 0.22 [0.09; 0.53] | 8.37 × 10^-3^ | 0.25 [0.1; 0.62] |
| 360.1599@7.2490993 |  | 7.25 | 361.1672 | 1.38 × 10-3 | 0.37 [0.22; 0.64] | 1.95 × 10^-3^ | 0.34 [0.18; 0.62] | 1.38 × 10^-2^ | 0.42 [0.23; 0.77] |
| 529.3506@7.2459974 |  | 7.25 | 530.3579 | 1.92 × 10-3 | 0.3 [0.15; 0.59] | 7.45 × 10^-3^ | 0.32 [0.15; 0.66] | 7.46 × 10^-3^ | 0.29 [0.12; 0.64] |
| 647.7965@0.5671946 |  | 0.57 | 648.8038 | 4.34 × 10-7 | 5.34 [2.91; 10.09] | 8.28 × 10^-6^ | 5.42 [2.77; 11.03] | 3.94 × 10^-6^ | 5.37 [2.76; 10.87] |
| 791.7583@0.56744844 |  | 0.57 | 792.7656 | 1.55 × 10-7 | 4.96 [2.83; 8.95] | 4.70 × 10^-6^ | 4.78 [2.62; 9.08] | 9.81 × 10^-7^ | 5.29 [2.83; 10.29] |
| 689.7772@0.56761014 |  | 0.57 | 690.7845 | 3.69 × 10-6 | 3.86 [2.27; 6.74] | 4.52 × 10^-5^ | 3.78 [2.12; 6.98] | 1.89 × 10^-5^ | 4.22 [2.28; 8.13] |
| 75.0685@0.59734464 |  | 0.60 | 76.0758 | 5.92 × 10-11 | 3.25 [2.31; 4.7] | 1.48 × 10^-7^ | 2.89 [2; 4.36] | 1.42 × 10^-10^ | 4.06 [2.63; 6.59] |
| 139.0611@0.63959837 |  | 0.64 | 140.0684 | 4.04 × 10-5 | 0.06 [0.02; 0.2] | 9.43 × 10^-5^ | 0.04 [0.01; 0.18] | 9.22 × 10^-4^ | 0.08 [0.02; 0.31] |
| 154.0732@1.4966037 |  | 1.50 | 155.0805 | 1.16 × 10-2 | 2.02 [1.25; 3.33] | 3.24 × 10^-2^ | 1.94 [1.16; 3.33] | 1.17 × 10^-2^ | 2.15 [1.27; 3.74] |
| 145.1102@2.52348 |  | 2.52 | 146.1175 | 9.23 × 10-4 | 2.89 [1.65; 5.11] | 1.49 × 10^-2^ | 2.48 [1.33; 4.71] | 2.05 × 10^-4^ | 3.78 [2; 7.38] |
| 277.1261@6.9963264 |  | 7.00 | 278.1334 | 7.75 × 10-3 | 0.28 [0.13; 0.63] | 3.39 × 10^-2^ | 0.3 [0.11; 0.76] | 2.51 × 10^-3^ | 0.17 [0.06; 0.48] |
| 413.2879@7.2710147 |  | 7.27 | 414.2952 | 4.39 × 10-5 | 0.09 [0.03; 0.25] | 3.25 × 10^-4^ | 0.09 [0.03; 0.3] | 3.61 × 10^-4^ | 0.09 [0.03; 0.3] |
| 452.8007@7.3034782 |  | 7.30 | 453.8080 | 3.06 × 10-5 | 0.07 [0.02; 0.22] | 7.37 × 10^-4^ | 0.09 [0.03; 0.32] | 1.56 × 10^-5^ | 0.04 [0.01; 0.16] |
| 861.7912@0.5556589 |  | 0.56 | 862.7985 | 3.87 × 10-7 | 0.16 [0.08; 0.31] | 6.94 × 10^-6^ | 0.16 [0.08; 0.33] | 2.26 × 10^-6^ | 0.15 [0.07; 0.31] |
| 989.7773@0.55061495 |  | 0.55 | 990.7846 | 5.87 × 10-5 | 0.21 [0.1; 0.42] | 2.08 × 10^-4^ | 0.2 [0.09; 0.42] | 1.63 × 10^-4^ | 0.19 [0.08; 0.41] |
| 190.0329@0.62505335 |  | 0.63 | 191.0402 | 1.06 × 10-2 | 0.27 [0.11; 0.65] | 3.08 × 10^-2^ | 0.28 [0.1; 0.74] | 1.59 × 10^-2^ | 0.23 [0.08; 0.65] |
| 619.9191@7.183109 |  | 7.18 | 620.9264 | 2.77 × 10-5 | 0.15 [0.06; 0.34] | 6.70 × 10^-4^ | 0.18 [0.07; 0.43] | 3.12 × 10^-5^ | 0.12 [0.04; 0.3] |
| 338.1757@7.477618 |  | 7.48 | 339.1830 | 8.55 × 10-4 | 0.33 [0.18; 0.6] | 2.95 × 10^-3^ | 0.35 [0.18; 0.64] | 4.09 × 10^-3^ | 0.33 [0.16; 0.65] |
| 283.153@6.8945346 |  | 6.89 | 284.1603 | 1.92 × 10-3 | 0.23 [0.1; 0.53] | 4.87 × 10^-3^ | 0.22 [0.09; 0.55] | 2.86 × 10^-3^ | 0.19 [0.07; 0.5] |
| 298.1252@6.8941784 |  | 6.89 | 299.1325 | 1.79 × 10-2 | 0.38 [0.19; 0.76] | 4.28 × 10^-2^ | 0.39 [0.18; 0.84] | 2.80 × 10^-2^ | 0.36 [0.16; 0.78] |
| 499.2678@6.90488 |  | 6.90 | 500.2751 | 1.39 × 10-4 | 4.45 [2.21; 9.17] | 1.48 × 10^-3^ | 3.9 [1.85; 8.51] | 5.80 × 10^-4^ | 4.5 [2.08; 10.11] |
| 535.7933@6.928881 |  | 6.93 | 536.8006 | 2.32 × 10-3 | 0.35 [0.19; 0.64] | 2.48 × 10^-2^ | 0.43 [0.22; 0.8] | 9.92 × 10^-4^ | 0.29 [0.14; 0.56] |
| 589.9252@7.185035 |  | 7.19 | 590.9325 | 1.12 × 10-2 | 0.25 [0.09; 0.64] | 3.38 × 10^-2^ | 0.27 [0.09; 0.74] | 1.89 × 10^-2^ | 0.23 [0.08; 0.67] |
| 524.3693@7.239511 |  | 7.24 | 525.3766 | 1.10 × 10-4 | 0.49 [0.36; 0.68] | 2.07 × 10^-3^ | 0.52 [0.36; 0.75] | 6.27 × 10^-5^ | 0.42 [0.28; 0.62] |
| 245.0312@3.3192885 |  | 3.32 | 246.0385 | 6.06 × 10-4 | 0.23 [0.11; 0.49] | 3.29 × 10^-3^ | 0.25 [0.11; 0.56] | 1.02 × 10^-3^ | 0.2 [0.08; 0.47] |
| 147.0357@0.85333717 |  | 0.85 | 148.0430 | 7.62 × 10-4 | 2.07 [1.42; 3.04] | 2.66 × 10^-4^ | 2.41 [1.58; 3.74] | 2.09 × 10^-2^ | 1.76 [1.17; 2.69] |
| 603.4426@7.1844187 |  | 7.18 | 604.4499 | 9.68 × 10-6 | 0.11 [0.04; 0.27] | 4.55 × 10^-4^ | 0.15 [0.06; 0.39] | 5.42 × 10^-6^ | 0.07 [0.02; 0.21] |
| 895.7838@0.55388147 |  | 0.55 | 896.7911 | 1.06 × 10-4 | 0.21 [0.1; 0.42] | 3.29 × 10^-4^ | 0.2 [0.09; 0.43] | 2.63 × 10^-4^ | 0.18 [0.08; 0.42] |
| 432.9725@0.79971904 |  | 0.80 | 433.9798 | 3.52 × 10-3 | 0.15 [0.05; 0.46] | 3.79 × 10^-2^ | 0.21 [0.06; 0.72] | 1.25 × 10^-3^ | 0.07 [0.02; 0.31] |
| 905.8161@0.5458809 |  | 0.55 | 906.8234 | 3.81 × 10-4 | 0.27 [0.14; 0.52] | 7.06 × 10^-4^ | 0.25 [0.12; 0.51] | 1.21 × 10^-3^ | 0.27 [0.13; 0.55] |
| 197.1782@5.755559 |  | 5.76 | 198.1855 | 1.92 × 10-3 | 0.1 [0.02; 0.36] | 1.00 × 10^-3^ | 0.07 [0.01; 0.28] | 1.83 × 10^-2^ | 0.13 [0.03; 0.57] |
| 384.1751@7.315471 |  | 7.32 | 385.1824 | 1.29 × 10-3 | 0.36 [0.2; 0.63] | 9.15 × 10^-3^ | 0.41 [0.23; 0.74] | 2.53 × 10^-3^ | 0.32 [0.16; 0.62] |
| 585.2973@7.028549 |  | 7.03 | 586.3046 | 1.83 × 10-2 | 2.47 [1.29; 4.82] | 1.88 × 10^-2^ | 2.76 [1.34; 5.87] | 3.90 × 10^-2^ | 2.54 [1.2; 5.58] |
| 155.0348@0.5332084 |  | 0.53 | 156.0421 | 1.87 × 10-2 | 5.66 × 10^+1^ [3.06 × 10; 1.15 × 10^+3^] | 6.02 × 10^-2^ | 34.73 [1.62; 866.14] | 1.31 × 10^-2^ | 153 [4.79 × 10; 5.69 × 10^+3^] |
| 869.7819@0.56351733 |  | 0.56 | 870.7892 | 1.36 × 10-2 | 0.25 [0.09; 0.65] | 8.58 × 10^-2^ | 0.34 [0.11; 0.94] | 1.76 × 10^-3^ | 0.09 [0.02; 0.35] |
| 801.7892@0.56466484 |  | 0.56 | 802.7965 | 3.90 × 10-2 | 0.29 [0.1; 0.79] | 1.55 × 10^-1^ | 0.4 [0.12; 1.11] | 2.35 × 10^-2^ | 0.15 [0.03; 0.65] |
| 377.8942@0.5645591 |  | 0.56 | 378.9015 | 1.78 × 10-2 | 0.17 [0.04; 0.6] | 8.88 × 10^-2^ | 0.25 [0.05; 0.93] | 1.09 × 10^-2^ | 0.08 [0.01; 0.46] |
| 202.0453@0.64058465 |  | 0.64 | 203.0526 | 1.93 × 10-2 | 0.21 [0.07; 0.65] | 1.16 × 10^-1^ | 0.32 [0.09; 1.02] | 8.50 × 10^-3^ | 0.13 [0.03; 0.5] |
| 382.1102@0.64193404 |  | 0.64 | 383.1175 | 3.80 × 10-2 | 0.38 [0.17; 0.83] | 1.65 × 10^-1^ | 0.5 [0.22; 1.1] | 2.05 × 10^-2^ | 0.29 [0.11; 0.72] |
| 60.0327@0.65709114 |  | 0.66 | 61.0400 | 3.46 × 10-2 | 3.42 [1.29; 9.45] | 2.08 × 10^-1^ | 2.27 [0.82; 6.54] | 4.36 × 10^-3^ | 6.54 [2.07; 22.08] |
| 246.0841@0.8334142 |  | 0.83 | 247.0914 | 3.03 × 10-2 | 3.15 [1.29; 8.05] | 1.13 × 10^-1^ | 2.51 [0.99; 6.78] | 1.21 × 10^-2^ | 4.91 [1.65; 15.93] |
| 99.0686@2.1264505 |  | 2.13 | 100.0759 | 4.30 × 10-2 | 1.43 [1.07; 1.91] | 2.68 × 10^-1^ | 1.24 [0.92; 1.69] | 9.69 × 10^-3^ | 1.65 [1.18; 2.34] |
| 184.122@2.2564888 |  | 2.26 | 185.1293 | 2.45 × 10-2 | 2.1 [1.2; 3.73] | 1.14 × 10^-1^ | 1.75 [0.99; 3.16] | 1.35 × 10^-2^ | 2.55 [1.34; 5.03] |
| 184.1217@2.470015 |  | 2.47 | 185.1290 | 1.62 × 10-2 | 2.16 [1.25; 3.78] | 9.36 × 10^-2^ | 1.79 [1.03; 3.18] | 8.02 × 10^-3^ | 2.65 [1.41; 5.16] |
| 387.2463@3.4327416 |  | 3.43 | 388.2536 | 1.45 × 10-2 | 0.41 [0.2; 0.78] | 3.11 × 10^-1^ | 0.62 [0.28; 1.26] | 1.57 × 10^-3^ | 0.32 [0.15; 0.62] |
| 155.0954@3.5814083 |  | 3.58 | 156.1027 | 1.53 × 10-2 | 1.48 [1.13; 1.95] | 1.25 × 10^-1^ | 1.34 [0.99; 1.82] | 2.27 × 10^-3^ | 1.72 [1.26; 2.37] |
| 536.3018@7.0521617 |  | 7.05 | 537.3091 | 1.83 × 10-2 | 0.38 [0.19; 0.76] | 7.51 × 10^-2^ | 0.43 [0.2; 0.92] | 4.85 × 10^-3^ | 0.25 [0.1; 0.58] |
| 507.3695@7.228767 |  | 7.23 | 508.3768 | 4.60 × 10-2 | 0.46 [0.24; 0.88] | 1.77 × 10^-1^ | 0.56 [0.28; 1.1] | 2.21 × 10^-2^ | 0.34 [0.15; 0.75] |
| 390.2672@8.70742 |  | 8.71 | 391.2745 | 3.91 × 10-2 | 4.29 [1.32; 14.58] | 1.12 × 10^-1^ | 3.63 [1; 13.8] | 4.51 × 10^-2^ | 4.68 [1.31; 17.65] |
| 397.7542@8.709816 |  | 8.71 | 398.7615 | 3.75 × 10-2 | 5.33 [1.38; 21.97] | 6.97 × 10^-2^ | 5.27 [1.19; 24.77] | 4.97 × 10^-2^ | 5.67 [1.32; 26.35] |
| 247.1612@4.882458 |  | 4.88 | 248.1685 | 2.45 × 10-2 | 0.35 [0.15; 0.77] | 6.90 × 10^-2^ | 0.38 [0.16; 0.9] | 2.62 × 10^-2^ | 0.31 [0.12; 0.75] |
| 275.1925@5.4239974 |  | 5.42 | 276.1998 | 1.72 × 10-2 | 0.26 [0.09; 0.68] | 7.69 × 10^-2^ | 0.33 [0.11; 0.91] | 8.37 × 10^-3^ | 0.19 [0.06; 0.57] |
| 299.1308@6.894106 |  | 6.89 | 300.1381 | 1.47 × 10-2 | 0.38 [0.19; 0.74] | 7.63 × 10^-2^ | 0.45 [0.21; 0.93] | 1.22 × 10^-2^ | 0.31 [0.14; 0.69] |
| 423.1937@6.941953 |  | 6.94 | 424.2010 | 1.15 × 10-2 | 0.35 [0.17; 0.71] | 6.05 × 10^-2^ | 0.4 [0.18; 0.89] | 6.97 × 10^-3^ | 0.29 [0.13; 0.64] |
| 453.7809@7.2253165 |  | 7.23 | 454.7882 | 1.06 × 10-2 | 0.38 [0.19; 0.73] | 5.19 × 10^-2^ | 0.43 [0.21; 0.87] | 8.53 × 10^-3^ | 0.34 [0.16; 0.69] |
| 208.0486@0.8375868 |  | 0.84 | 209.0559 | 4.77 × 10-2 | 3.63 [1.23; 11.45] | 2.37 × 10^-1^ | 2.32 [0.77; 7.55] | 7.13 × 10^-3^ | 8.84 [2.16; 40.51] |
| 180.065@2.0888093 |  | 2.09 | 181.0723 | 3.25 × 10-2 | 1.38 [1.07; 1.78] | 5.57 × 10^-2^ | 1.38 [1.05; 1.82] | 3.21 × 10^-2^ | 1.46 [1.09; 1.98] |
| 318.1217@4.5192366 |  | 4.52 | 319.1290 | 1.25 × 10-2 | 0.32 [0.14; 0.7] | 1.48 × 10^-1^ | 0.47 [0.19; 1.08] | 3.39 × 10^-3^ | 0.25 [0.1; 0.57] |
| 359.2673@5.3253756 |  | 5.33 | 360.2746 | 1.93 × 10-2 | 0.26 [0.09; 0.7] | 6.54 × 10^-2^ | 0.31 [0.1; 0.87] | 1.20 × 10^-2^ | 0.2 [0.06; 0.6] |
| 609.9216@7.1840405 |  | 7.18 | 610.9289 | 1.93 × 10-2 | 0.2 [0.06; 0.64] | 9.97 × 10^-2^ | 0.27 [0.07; 0.97] | 1.15 × 10^-2^ | 0.15 [0.04; 0.53] |
| 342.9925@0.84931105 |  | 0.85 | 343.9998 | 3.47 × 10-2 | 2.98 [1.25; 7.17] | 8.18 × 10^-2^ | 2.71 [1.08; 7] | 2.30 × 10^-2^ | 3.85 [1.42; 10.97] |
| 143.1674@2.2895062 |  | 2.29 | 144.1747 | 1.88 × 10-2 | 0.48 [0.27; 0.82] | 9.87 × 10^-2^ | 0.57 [0.31; 0.98] | 4.31 × 10^-3^ | 0.39 [0.2; 0.7] |
| 160.1212@0.5786255 |  | 0.58 | 161.1285 | 1.12 × 10-2 | 0.46 [0.22; 0.8] | 1.18 × 10^-1^ | 0.56 [0.26; 1.01] | 2.15 × 10^-3^ | 0.39 [0.18; 0.7] |
| 103.0454@0.8523692 |  | 0.85 | 104.0527 | 1.84 × 10-1 | 0.41 [0.14; 1.2] | 6.86 × 10^-1^ | 0.71 [0.22; 2.38] | 3.73 × 10^-2^ | 0.2 [0.05; 0.72] |
| 132.0255@0.8537359 |  | 0.85 | 133.0328 | 2.37 × 10-1 | 0.41 [0.13; 1.38] | 8.07 × 10^-1^ | 0.79 [0.24; 3.19] | 1.90 × 10^-2^ | 0.09 [0.01; 0.51] |
| 168.0891@1.7759738 |  | 1.78 | 169.0964 | 9.39 × 10-2 | 1.73 [1.02; 2.96] | 3.21 × 10^-1^ | 1.46 [0.82; 2.63] | 4.63 × 10^-2^ | 2.02 [1.13; 3.67] |
| 145.0527@3.9742987 |  | 3.97 | 146.0600 | 5.48 × 10-2 | 1.95 [1.1; 3.51] | 1.71 × 10^-1^ | 1.69 [0.92; 3.15] | 2.05 × 10^-2^ | 2.48 [1.28; 4.94] |
| 454.2838@7.2246428 |  | 7.22 | 455.2911 | 5.21 × 10-2 | 0.39 [0.18; 0.87] | 1.81 × 10^-1^ | 0.48 [0.2; 1.14] | 3.74 × 10^-2^ | 0.33 [0.13; 0.8] |
| 444.7802@7.228973 |  | 7.23 | 445.7875 | 6.19 × 10-2 | 0.43 [0.2; 0.91] | 1.91 × 10^-1^ | 0.5 [0.21; 1.15] | 2.34 × 10^-2^ | 0.33 [0.14; 0.75] |
| 265.0036@0.6355312 |  | 0.64 | 266.0109 | 5.52 × 10-2 | 0.16 [0.03; 0.78] | 1.55 × 10^-1^ | 0.22 [0.04; 1.19] | 4.28 × 10^-2^ | 0.12 [0.02; 0.68] |
| 343.2202@3.1561654 |  | 3.16 | 344.2275 | 2.29 × 10-1 | 0.53 [0.2; 1.2] | 8.76 × 10^-1^ | 1.14 [0.39; 3.14] | 2.22 × 10^-2^ | 0.3 [0.1; 0.75] |
| 809.9669@6.9071665 |  | 6.91 | 810.9742 | 6.69 × 10-2 | 0.44 [0.21; 0.92] | 2.18 × 10^-1^ | 0.54 [0.24; 1.18] | 2.83 × 10^-2^ | 0.33 [0.14; 0.77] |
| 61.035@0.65672415 |  | 0.66 | 62.0423 | 8.95 × 10-2 | 2.77 [1.04; 7.65] | 3.11 × 10^-1^ | 1.98 [0.71; 5.77] | 2.30 × 10^-2^ | 4.54 [1.47; 14.94] |
| 131.0374@2.8586442 |  | 2.86 | 132.0447 | 6.52 × 10-21 | 5.48 [3.76; 8.25] | 1.39 × 10^-15^ | 5 [3.31; 7.93] | 2.77 × 10^-18^ | 6.03 [3.88; 9.91] |
| 281.9999@3.7437904 |  | 3.74 | 283.0072 | 4.62 × 10-2 | 0.45 [0.23; 0.88] | 1.03 × 10^-1^ | 0.48 [0.23; 0.99] | 5.15 × 10^-2^ | 0.41 [0.19; 0.87] |
| 114.0423@0.84433424 |  | 0.84 | 115.0496 | 4.99 × 10-2 | 0.31 [0.1; 0.84] | 1.57 × 10^-1^ | 0.38 [0.12; 1.12] | 3.93 × 10^-2^ | 0.28 [0.09; 0.78] |

**^a^** RT: retention time in minutes.

^b^ m/z: mass-to-charge ratio.

^c^ q-value: FDR-corrected p-value. Marked in red are statistically not significant q-values > 0.05.

^d^ OR: Odds ratio, with a one standard deviation change in metabolite intensity representing relative changes in CRC risk. Positive or inverse associations with CRC are characterized by an OR > 1 or < 1, respectively.
