# Supplementary material for: Untargeted Metabolomics Reveals Major Differences in the Plasma Metabolome between Colorectal Cancer and Colorectal Adenomas
Source: Metabolites. 2021 Feb 19;11(2):119. doi: 10.3390/metabo11020119 (PMC7922413; doi:10.3390/metabo11020119)
Supplement: Supplementary file 1 [file metabolites-11-00119-s001.zip › metabolites-1056311-supple-for conversion/Supplementary Table S2 revised _ proof.docx]

**Supplementary Table S2.** List of outcome-specific metabolic features.

| **CRC vs HR + LR** | | | | **CRC vs HR** | | | | **CRC vs LR** | | | |
| --- | --- | --- | --- | --- | --- | --- | --- | --- | --- | --- | --- |
| **Tentative identity** | **RT ^a^** | **m/z ^b^** | **q-value ^c^** | **Tentative identity** | **RT ^a^** | **m/z ^b^** | **q-value ^c^** | **Tentative identity** | **RT ^a^** | **m/z ^b^** | **q-value ^c^** |
| Choline | 4.7 × 10^-2^ | 104.1077 | 0.58 | 247.1429@0.85507876 | 1.66 × 10^-2^ | 248.1502 | 0.86 | 103.0454@0.8523692 | 3.73 × 10^-2^ | 104.0527 | 0.85 |
| 248.1002@0.6071271 | 4.3 × 10^-2^ | 249.1075 | 0.61 | 229.9752@0.86571616 | 4.19 × 10^-2^ | 230.9825 | 0.87 | 132.0255@0.8537359 | 1.90 × 10^-2^ | 133.0328 | 0.85 |
| Valine | 2.9 × 10^-2^ | 118.0866 | 0.80 | 188.0004@0.8802064 | 3.52 × 10^-2^ | 189.0077 | 0.88 | 168.0891@1.7759738 | 4.63 × 10^-2^ | 169.0964 | 1.78 |
| 157.1107@0.8298541 | 3.6 × 10^-2^ | 158.1180 | 0.83 | 103.0192@0.8879074 | 3.79 × 10^-2^ | 104.0265 | 0.89 | LysoPC (14:0) (isomer 1) | 1.61 × 10^-2^ | 468.3090 | 6.65 |
| 281.9999@3.7437904 | 4.6 × 10^-2^ | 283.0072 | 3.74 | 230.0112@0.8899939 | 2.74 × 10^-2^ | 231.0185 | 0.89 | LysoPC (14:0) (isomer 2) | 2.82 × 10^-2^ | 490.2906 | 6.73 |
| 422.7614@8.752114 | 3.6 × 10^-2^ | 423.7687 | 8.75 | 196.9783@0.899427 | 1.03 × 10^-2^ | 197.9856 | 0.90 | Lyso PC (20:5) | 2.05 × 10^-2^ | 542.3229 | 6.77 |
| [430.7503@8.751049](mailto:157.1107@0.8298541) | 3.1 × 10^-2^ | 431.7576 | 8.75 | 202.9642@1.500623 | 3.94 × 10^-2^ | 203.9715 | 1.50 | LysoPC (16:0) | 3.16 × 10^-2^ | 276.1735 | 7.00 |
| Bilirubin isomer (2) | 4.3 × 10^-2^ | 616.2522 | 5.12 | 188.0003@1.7456483 | 3.77 × 10^-2^ | 189.0076 | 1.75 | 145.0527@3.9742987 | 2.05 × 10^-2^ | 146.0600 | 3.97 |
| 318.1477@6.8379445 | 4.3 × 10^-2^ | 319.1550 | 6.84 | 317.1134@0.8575037 | 1.76 × 10^-2^ | 318.1207 | 0.86 | LysoPC (18:1) | 4.70 × 10^-2^ | 809.9861 | 7.06 |
|  |  |  |  | 203.9708@0.88541883 | 3.31 × 10^-2^ | 204.9781 | 0.89 | 454.2838@7.2246428 | 3.74 × 10^-2^ | 455.2911 | 7.22 |
|  |  |  |  | 233.962@0.89373153 | 3.91 × 10^-2^ | 234.9693 | 0.89 | 444.7802@7.228973 | 2.34 × 10^-2^ | 445.7875 | 7.23 |
|  |  |  |  | 187.9999@1.5010325 | 3.53 × 10^-2^ | 189.0072 | 1.50 | 265.0036@0.6355312 | 4.28 × 10^-2^ | 266.0109 | 0.64 |
|  |  |  |  | 342.1865@7.248144 | 4.26 × 10^-2^ | 343.1938 | 7.25 | 343.2202@3.1561654 | 2.22 × 10^-2^ | 344.2275 | 3.16 |
|  |  |  |  | 205.9733@1.5004629 | 4.26 × 10^-2^ | 206.9806 | 1.50 | 809.9669@6.9071665 | 2.83 × 10^-2^ | 810.9742 | 6.91 |
|  |  |  |  | 188.9841@1.5000874 | 2.48 × 10^-2^ | 189.9914 | 1.50 | 61.035@0.65672415 | 2.30 × 10^-2^ | 62.0423 | 0.66 |
|  |  |  |  | Nonanoylcarnitine (C9:0) | 1.47 × 10^-2^ | 302.2323 | 4.63 |  |  |  |  |
|  |  |  |  | 210.124@5.7608237 | 3.08 × 10^-2^ | 211.1313 | 5.76 |  |  |  |  |
|  |  |  |  | 204.0208@1.0509856 | 2.87 × 10^-2^ | 205.0281 | 1.05 |  |  |  |  |

^a^ RT: retention time in minutes.

^b^ m/z: mass-to-charge ratio.

^C^ q-value: FDR-corrected p-value.
